# Structure–activity relationship of ipglycermide binding to phosphoglycerate mutases

**DOI:** 10.1016/j.jbc.2021.100628

**Published:** 2021-04-01

**Authors:** Mareike Wiedmann, Patricia K. Dranchak, Mahesh Aitha, Bryan Queme, Christopher D. Collmus, Maithri M. Kashipathy, Liza Kanter, Laurence Lamy, Joseph M. Rogers, Dingyin Tao, Kevin P. Battaile, Ganesha Rai, Scott Lovell, Hiroaki Suga, James Inglese

**Affiliations:** 1 Department of Chemistry, Graduate School of Sciences, The University of Tokyo, Tokyo, Japan; 2 National Center for Advancing Translational Sciences, National Institutes of Health, Rockville, Maryland, USA; 3 Protein Structure Laboratory, Structural Biology Center, University of Kansas, Lawrence, Kansas, USA; 4 IMCA-CAT Advanced Photon Source, Argonne National Laboratory, Argonne, Illinois, USA; 5 National Human Genome Research Institute, National Institutes of Health, Bethesda, Maryland, USA

**Keywords:** affinity selection, binding kinetics, crystallography, cyclic peptides, glycolysis, nematode, protein dynamics, infectious disease, solid phase peptide synthesis, inhibitor, DMSO, dimethyl sulfoxide, dPGM, cofactor-dependent phosphoglycerate mutase, iPGM, cofactor-independent phosphoglycerate mutase, PDB, Protein Data Bank, PGM, phosphoglycerate mutase, RaPID, Random Non-standard Peptide Integrated Discovery, SPPS, solid-phase peptide synthesis, SPR, surface plasmon resonance

## Abstract

Catalysis of human phosphoglycerate mutase is dependent on a 2,3-bisphosphoglycerate cofactor (dPGM), whereas the nonhomologous isozyme in many parasitic species is cofactor independent (iPGM). This mechanistic and phylogenetic diversity offers an opportunity for selective pharmacologic targeting of glycolysis in disease-causing organisms. We previously discovered ipglycermide, a potent inhibitor of iPGM, from a large combinatorial cyclic peptide library. To fully delineate the ipglycermide pharmacophore, herein we construct a detailed structure–activity relationship using 280 substituted ipglycermide analogs. Binding affinities of these analogs to immobilized *Caenorhabditis elegans* iPGM, measured as fold enrichment relative to the index residue by deep sequencing of an mRNA display library, illuminated the significance of each amino acid to the pharmacophore. Using cocrystal structures and binding kinetics, we show that the high affinity of ipglycermide for iPGM orthologs, from *Brugia malayi*, *Onchocerca volvulus*, *Dirofilaria immitis*, and *Escherichia coli*, is achieved by a codependence between (1) the off-rate mediated by the macrocycle Cys14 thiolate coordination to an active-site Zn^2+^ in the iPGM phosphatase domain and (2) shape complementarity surrounding the macrocyclic core at the phosphotransferase–phosphatase domain interface. Our results show that the high-affinity binding of ipglycermide to iPGMs freezes these structurally dynamic enzymes into an inactive, stable complex.

The essential role of glycolysis to the viability of many parasites predisposes the pathway’s enzymes as anti-infective drug candidates (1, 2, 3). However, the evolutionarily conserved nature of most glycolytic enzymes creates a significant possibility of mechanism-based toxicity in human or animal hosts (4). Therefore, cases where the glycolytic activity is encoded by catalytically divergent isozymes greatly favors species selectivity. Such a situation distinguishes phosphoglycerate mutase (PGM) in nematodes (5), trypanosomes (6), *Mycoplasma* (7), and several gram-positive pathogenic bacteria from the mammalian enzyme (8, 9). In all vertebrates, cofactor-dependent phosphoglycerate mutase (dPGM) is a 2, 3-bisphophoglycerate cofactor–utilizing homodimer consisting of 23-kDa subunits, while the parasitic enzyme is a 56-kDa monomer cofactor-independent enzyme consisting of a phosphatase domain connected by a flexible hinge to the phosphotransferase domain. The catalytic mechanism utilized by these isozymes is also remarkably distinct. In dPGM, an active site phosphohistidine (H10) equilibrates the phosphate between the 2 and 3 positions of glyceric acid, while in cofactor-independent phosphoglycerate mutase (iPGM), a transition metal ion–activated serine (S86) mediates the phosphate transfer (10).

RNA interference experiments designed to silence iPGM in *Caenorhabditis elegans* demonstrated embryonic and larval lethality in addition to morphological effects (5). This functional target validation study spurred high-throughput screening for small-molecule iPGM inhibitors as candidates for a drug development program. In that work, either *C. elegans* or *B. malayi* iPGM activity was coupled through enolase and pyruvate kinase to an lactate dehydrogenase-mediated NADH absorbance assay, which was then used to screen small-molecule library collections totaling ∼380,000 compounds (11). Unfortunately, with little more than several low potency (∼10 μM) metal-ion chelators, the outcome suggests small-molecule inhibitors of iPGM may be particularly challenging to develop.

Interestingly, the catalytic mechanism of iPGM involves a dynamically formed interdomain active site that sequesters phosphoglycerate substrate electrostatically bound at the transferase domain surface (12). This catalytic process evolved to isomerize phosphoglycerate, a small, charged hydrophilic molecule, without the ligand-binding pockets often found in enzymes such as G protein-coupled receptors and kinases that are often exploited in drug discovery (13, 14). Taken together, the existing data support and explain both the refractory nature of iPGM toward inhibition by Lipinski-like small molecules and the enzyme sensitivity to nonspecific metal-ion chelators (15, 16). We therefore explored a different strategy to search for iPGM inhibitors based on vastly larger chemical space (>10^12^ structures), molecular rigidity, and complexity encompassed by nucleic acid–encoded cyclic peptide libraries (17). Using affinity selection to isolate and enrich mRNA-encoded macrocyclic peptides tightly bound to immobilized *C. elegans* iPGM, we discovered ipglycermide, a highly potent macrocyclic chemotype inhibitor of the enzyme and its nematode species orthologs (18).

Ipglycermide is a 14-amino acid peptide containing an 8-membered macrocycle formed through a thioether bridging the D-Tyr_1_ α-acetamide and Cys8 sulfhydryl (Fig. 1*A*).† At the carboxyl terminus of ipglycermide, we proposed that the Cys14 thiol coordinates with a transition ion adjacent to the catalytic Zn^2+^-coordinated Ser_86_ of the enzyme based on the requirement of Cys14 for subnanomolar inhibition of *C. elegans* iPGM and modeling from the cocrystal structure of a truncated ipglycermide analog, Ce-2d (Fig. 1*B*), bound to *C. elegans* iPGM (18).

**Figure 1:**
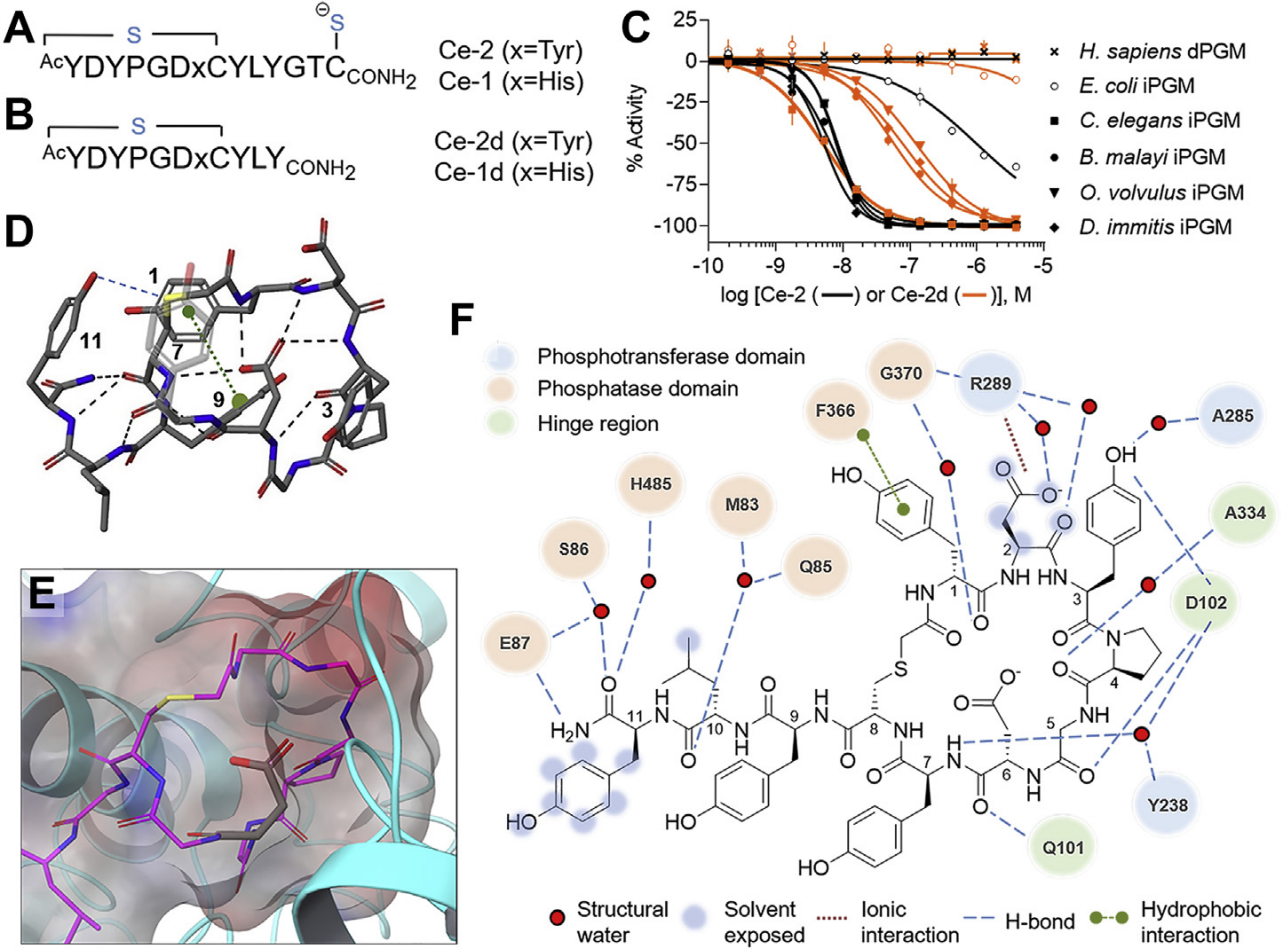
**Ipglycermide architecture and properties.** Sequence and motif organization of peptide sequences (*A*) Ce-1 and Ce-2 discovered in RaPID affinity selection and (*B*) core sequence, residues 1 to 11. *C*, concentration response curves demonstrating limits of the functional enzyme assay on measuring the inhibition of ipglycermide *versus* the ipglycermide core on iPGM ortholog activity. *D*, Ce-2d intramacrocyclic H-bonding mediated by the Asp6 carboxylate and Tyr9-Leu10-Tyr11 α-chain amides to macrocycle amide backbone hydrogens (*black dashed lines*) from cocrystal structures (tyrosine side chains are numbered and the Tyr7 side chain is transparent for clarity). *E*, electrostatic surface contour of ipglycermide macrocycle surrounding Asp6 side chain and α-chain backbone. Panels *D* and *E* are determined from PDB dataset 5KGN. *F*, Two-dimensional Ce-2d protein and structural water pharmacophore interactions. iPGM, cofactor-independent phosphoglycerate mutase; PDB, Protein Data Bank; RaPID, Random Non-standard Peptide Integrated Discovery.

In the present study, we extend and refine our structural and mechanistic understanding of ipglycermide with affinity selection-based saturation mutagenesis, surface plasmon resonance (SPR) determination of binding kinetics, and new cocrystallographic insights from metal-ion–coordinated ipglycermides. These findings will aid in the development of analogs with broader pathogen iPGM selectivity and as chemical probes to study the biochemistry and regulation of iPGM in microbial glycolysis. Furthermore, we anticipate ipglycermide will serve as a useful macrocyclic peptide for the study of cell-permeable analogs and formulations having potential therapeutic applications.

## Results and discussion

### Ipglycermide selectivity and core structure

Recombinant expression of PGMs from human, nematode, and bacterial species previously allowed us to show (18) absolute specificity of ipglycermide for iPGM over dPGM isozymes and correlate Ce-2d selectivity among iPGM orthologs from *C. elegans*, *B. malayi*, *Onchocerca volvulus*, and *Dirofilaria immitis* (Fig. 1*C*). Although nematode ortholog iPGM concentrations (≥1 nM) needed for robust enzymatic activity measurements are above the *K*_*D*_ of the parent ipglycermide Ce-2 for nematode orthologs, apparent from the characteristic steep concentration responses (Fig. 1*C*, *black curves* (19)), the lower-affinity Ce-2d ipglycermide core reveals an IC_50_ dependence on sequence differences among the orthologs (compare orange *versus* back curves in Fig. 1*C*). Furthermore, the low specific catalytic activity of *Escherichia coli* iPGM having 45% identity with *C. elegans* iPGM limits the ability to measure IC_50_ values below 50 nM for this enzyme, with Ce-2d showing no significant inhibition up to 30 μM.§ *E. coli* iPGM appears to demarcate the point in sequence similarity sensitive to the Ce-2d ipglycermide, whereas the enzyme activity of nematode orthologs tested (% sequence identity is between 71 and 75% of *C. elegans*) was stoichiometrically inhibited by Ce-2 and potently inhibited by Ce-2d (Fig. 1*C*).

From the cocrystal structure of the ipglycermide Ce-2d analog bound to *C. elegans* iPGM, we observe that the ipglycermide core (residues 1–11) is a compact structure with an internal H-bond network strongly mediated by Asp6 (Fig. 1*D* and Table S1). Although Asp6 is largely shielded from contact with iPGM (Fig. 1*E*), the remaining ipglycermide residues engage the enzyme interdomain cavity primarily along the phosphatase surface through water-mediated hydrogen bonding with minimal hydrophobic interactions (Fig. 1*F*).

### Ipglycermide metal-ion coordination

**Figure 2:**
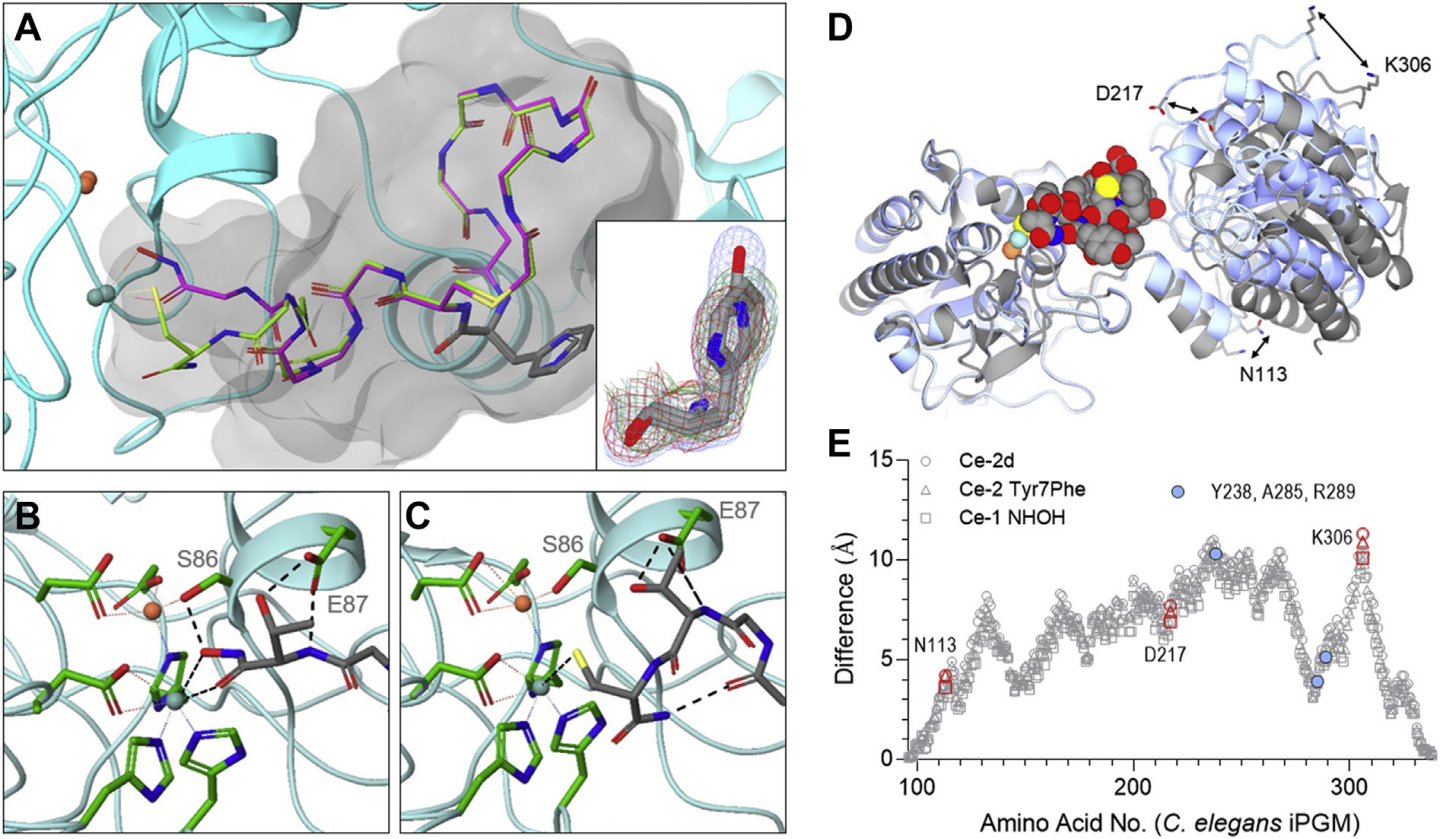
**Cocrystal structures of iPGM-bound ipglycermides elucidating the metal ion coordination and induced domain complementarity.***A*, superposition of the iPGM⋅Ce-2 Tyr7Phe (*magenta*) and the iPGM⋅Ce-1 NHOH (*green*) structures. Coordination of Cys14 thiolate of Ce-2 Tyr7Phe and hydroxamic acid of Ce-1 NHOH to the Zn ions (*sea-green* and *coral spheres*) is indicated by the *dotted lines*. Only main chain atoms are shown for clarity except for the metal ion–coordinating side chains, Asp6 and position 7 side chains, Phe and His. Fo-Fc at 5 sigma electron density overlays (*bottom right conner inset*) from Tyr7 (*blue mesh*), Phe7 (*green mesh*), and His7 (*red mesh*) cocrystal structures 5KGN, 7KNG, and 7KNF, respectively. *B*, bidentate hydroxamic acid metal ion coordination and Thr13–E87 interactions. *C*, details of Cys14 thiolate metal ion coordination and Thr13–E87 interactions and the carboxamide–Tyr11 carbonyl H-bond. *D*, superposition of the phosphatase regions of *Caenorhabditis elegans* iPGM in the ipglycermide-bound form and apo-form. Apo-iPGM (5KGM) is *gray* and ipglycermide-bound iPGM (7KNG) is *ice blue*. iPGM secondary structures are represented as *ribbons*. Ce-2 (Tyr7Phe) is represented in a space-filling model. The crystal structures were superimposed over the range from amino acids 27 to 95 and 339 to 538. *E*, distance differentials in the transferase domain spanning amino acids 96 to 196 and 198 to 338 determined from the co-crystal structures PDB ID 5KGN, 7KNF, and 7KNG. *Blue circles* refer to Y238, A285, and R289 in Figure 1*F*. Locations of N113, D217, and K306 (*red symbols*) are indicated in panel *D*. iPGM, cofactor-independent phosphoglycerate mutase.

To gain structural insight into the molecular interactions of the C-terminus of ipglycermides with iPGM, we established co-crystal structures using analogs, Ce-2 Tyr7Phe and Ce-1 Cys14NHOH (Ce-1 NHOH), respectively, retaining Cys14 or replacing it with NHOH, an oxidation-insensitive and solid-phase peptide synthesis (SPPS)-accessible metal-ion–coordinating ligand (20, 21, 22). Using our established crystallization conditions, supplemented with 2-mM tris(2-carboxyethyl)phosphine for Ce-2 Tyr7Phe–containing complexes, we were able to obtain crystals diffracting to 2.1 Å and 1.80 Å for Ce-2 Tyr7Phe and Ce-1 NHOH, respectively, bound to *C. elegans* iPGM (Table S2). The electron density maps for these complexes were clearly defined, and all atoms for the ipglycermides could be modeled except for the Ce-1 NHOH solvent-exposed Tyr11 side chain (Fig. S2). The “core” region of the peptide (Tyr1-Tyr11) forms hydrogen-bond interactions with N85, Q101, D102, and R289 like Ce-2d (Fig. S3 and Fig. 1*F*). Comparable hydrogen-bond interactions that were observed in iPGM⋅Ce-1 NHOH are present in the iPGM⋅Ce-2 Tyr7Phe structure (Fig. S4 and Table S3). The iPGM domain residues are nearly identical, and superposition yielded an RMSD of 0.24 Å between Cα atoms (Fig. 2*A*, 504 residues). Likewise, the Ce-1 NHOH and Ce-2 Tyr7Phe peptides adopt a similar binding mode (Fig. S4) in which residues 1 to 11 are positioned in nearly identical orientations to those for the *C. elegans* iPGM–Ce-2d complex (Protein Data Bank [PDB] ID: 5KGN) including water-mediated H-bonding (Fig. 1*F* and Table S3) and a coplanar orientation of the aromatic residue 7 substitutions, Tyr, Phe, and His, respectively, in Ce-2d, Ce-2 Tyr7Phe, and Ce-1 NHOH (Fig. 2*A* *insert* and Fig. S4).

However, residues 12 to 14 of Ce-2 Tyr7Phe and Ce-1 NHOH display nonoverlapping conformations resulting from differences in Zn^2+^ coordination. The C-terminal Thr13-hydroxamate residue side chain forms an interaction with E87, which appears to constrain this portion of the peptide and permits a bidentate interaction from the carbonyl (2.15 Å) and hydroxyl (2.30 Å) to a Zn^2+^. In addition, the Thr13 hydroxyl forms a hydrogen bond with the active site S86 that is coordinated to the second Zn^2+^ (Fig. 2*B*). In the iPGM–Ce-2 Tyr7Phe complex, the Cys14-NH_2_ residue forms a monodentate interaction with the Zn^2+^ through the S atom (2.28 Å) as suggested by prior modeling using the *C. elegans* iPGM–Ce-2d complex (Fig. 2*C*) (18). In addition, the Cys14-terminal amide NH_2_ group forms an intramolecular hydrogen bond with Tyr11 (Fig. 2*C*), possibly serving to brace and position the Cys14 thiolate. From this binding orientation, it can be envisioned how the nucleic acid attached to the C terminus of the original RNA-encoded ligand could be accommodated in the complex (Fig. S3*B*). The main change in the common sequence is observed for residue Thr13 that is displaced from the Zn^2+^ in iPGM–Ce-2 Tyr7Phe to accommodate the binding of Cys14. Notably, the sulfur atom of Cys14 is positioned in a nearly identical region as the Thr13/hydroxamate carbonyl in the iPGM⋅Ce-1 NHOH structure. Despite these conformational displacements, the Thr13 side-chain hydroxyl adopts a similar spatial position in both structures, thereby maintaining the hydrogen-bond interaction with the phosphatase domain E87 γ-carboxylate (Fig. S4).

### Ipglycermide-induced concavity

A comparative analysis of the apo–*C. elegans* iPGM structure (PBD ID: 5KGM) to that of the ipglycermide-bound forms (PDB ID: 5KGN, 7KNF, 7KNG) indicates the enzyme clamps the core by inward movement of the phosphotransferase and phosphatase domains by as much as 10 Å (Fig. 2, *D* and *E*). The movement would be consistent with the structural dynamics described for the catalytic mechanism of the *Trypanosoma brucei* and *Staphylococcus aureus* iPGM orthologs and observed motions of loops in other enzyme systems (12, 23, 24).

### Off-rate controls ipglycermide high-affinity binding

We sought to resolve the composite contribution of the ipglycermide core and metal-coordinating substructures to affinity and ortholog selectivity by determining dissociation constants based on association and dissociation kinetics for Ce-2d *versus* Ce-2 and Ce-1 NHOH. Therefore, we measured binding kinetics and saturation binding using the activity-independent technique of SPR to develop a structure–interaction kinetic relationship among the ipglycermide analogs and iPGM orthologs. For conditional immobilization of the iPGMs to the sensor chip surface, we chose to use reversible biotin capture (25). Consequently, we prepared site-specific biotin-labeled iPGMs through biotin ligase BirA-catalyzed biotin transfer to a 14-amino acid accepter sequence added to the C terminus of *C. elegans*, *B. malayi*, and *E. coli* iPGMs (26).

**Figure 3:**
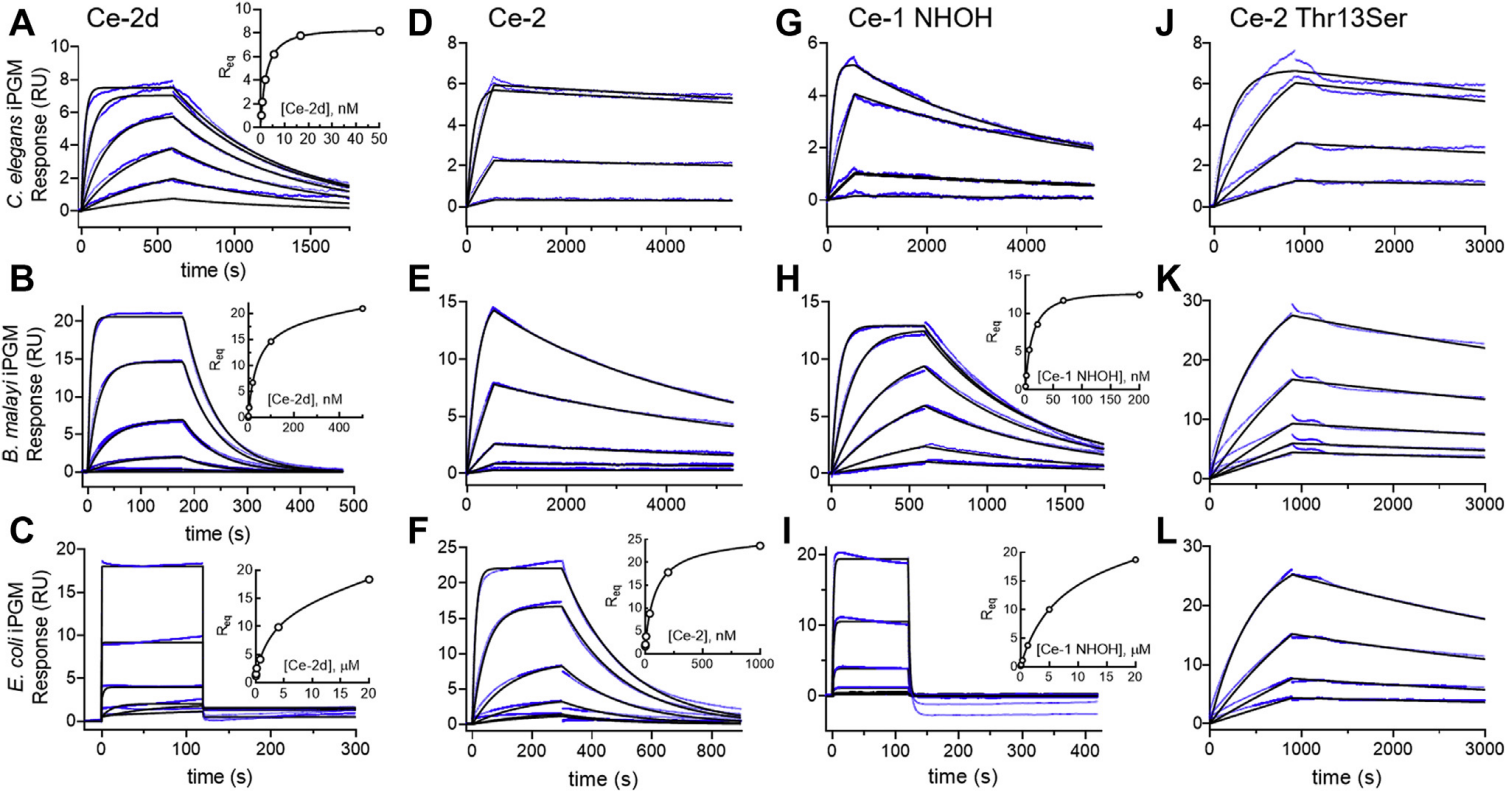
**SPR analysis of ipglycermides binding to iPGM orthologs.** Sensorgrams for (*A*) 0.206 to 50 nM, (*B*) 0.16 to 500 nM, and (*C*) 0.0064 to 20 μM Ce-2d binding; (*D*) 0.0098 to 2.5 nM, (*E*) 0.195 to 12.5 nM, and (*F*) 0.32 to 1000 nM Ce-2 binding; (*G*) 0.08 to 10 nM, (*H*) 0.82 to 200 nM, and (*I*) 0.19 to 20 μM Ce-1 NHOH binding: and (*J*) 0.04 to 2.5 nM, (*K*) 0.01 to 2.5 nM, and (*L*) 0.2 to 12.5 nM Ce-2 Thr13Ser binding to sensor chip-immobilized *Caenorhabditis elegans*, *Brugia malayi*, and *Escherichia coli* iPGM, respectively. The experimental data (*blue*) were globally fit to a 1:1 binding model (*black lines*) using BIAevaluation, as described in Experimental procedures, to determine kinetic rate constants. Corresponding insets show the signal observed at equilibrium, R_eq_, plotted as a function of ipglycermide analog concentration, fit to a hyperbolic, single-site binding equation. Data are representative of N ≥ 2 independent experiments. Site-specific biotin labeling of iPGMs is described in Experimental procedures.

For the sensorgram progress curves reaching the steady state (Fig. 3, *A*–*C*, *F*, *H*, and *I*), we calculated the *K*_D_ from the hyperbolic fit to a single-site binding equation. However, where progress curves did not reach equilibrium during the longest injection, the *K*_D_ was determined from the ratio of the rate constants and *k*_off_/*k*_on_ determined from the experimental data (blue curves) globally fit to a 1:1 binding model (black curves). A qualitative inspection of the resulting sensorgrams in Figure 3 clearly demonstrates the codependence of the metal ion–coordinating and interdomain-binding substructures in modulating the off-rate of ipglycermide from the three iPGM orthologs. Although *k*_on_ values between analogs and orthologs spanned a 450-fold range, *k*_off_ between *C. elegans* iPGM–Ce-2d and *E. coli* iPGM–Ce-2d interaction was >10^6^-fold, illustrating the predominating role of the off-rate on the *K*_D_. For each analog–iPGM binding interaction, a consistent trend in *t*_½_ can be observed, where Ce-2 Thr13Ser > Ce-2 > Ce-1 NHOH >> Ce-2d, respectively, for *C. elegans*, *B. malayi*, and *E. coli* iPGMs encompassing a *K*_D_ range of over 5 orders of magnitude (Table 1). *R*_eq_ binding measured for Ce-2d at the iPGM orthologs gives *K*_D_ values of 2.4 nM, 58 nM, and 6 μM for *C. elegans*, *B. malayi*, and *E. coli* iPGM, respectively, correlating well with the ∼40-fold ΔIC_50_ between *C. elegans* and *B. malayi* iPGM seen in the functional enzyme assays, while allowing us to measure an affinity on *E. coli* iPGM not possible with the limitations in the functional assay (Table 1 *versus* Fig. 1*C*). The *K*_D_ for Ce-2 on *C. elegans* iPGM determined from the *k*_on_ = 1.1 × 10^6^ M^−1^s^−1^ and *k*_off_ = 3.2 × 10^−5^ s^−1^ of 38 pM is within several folds of our prior estimate of 70 pM determined using a zone boundary analysis from a cross-titration of *C. elegans* iPGM enzyme *versus* Ce-2 (18). Regarding the low specific activity of *E. coli* iPGM, the SPR-derived Ce-2 *K*_D_ of ≈70 nM is consistent with an IC_50_ exceeding 100 nM in the functional assay requiring an enzyme concentration of 50 nM (Fig. 1*C*). Overall, *K*_D_ values are well correlated with the IC_50_ values of those analog-iPGM pairs that could be reliably measured by the functional assay. Using SPR, we could obtain affinity values for all the interacting species tested, which for Ce-2 were delimited by the enzyme concentrations required in the functional assay (Fig. 1*C*, *black curves*).

**Table 1:** Rate and dissociation constants of ipglycermide analogs binding to iPGM orthologs

Ipglycermide	iPGM	k_on_ (M^−1^s^−1^)	k_off_ (s^−1^)	K_D_	t_½_
Ce-2d	*C. elegans*	2.1 × 10^6^	2.1 × 10^−3^	2.4 nM	6 min
Ce-2	*C. elegans*	1.1 × 10^6^	3.2 × 10^−5^	38 pM	6.7 h
Ce-1 NHOH	*C. elegans*	1.9 × 10^6^	7.8 × 10^−5^	110 pM	1.5 h
Ce-2 Thr13Ser	*C. elegans*	2.9 × 10^6^	1.7 × 10^−4^	29 pM	2.5 h
Ce-2d	*B. malayi*	5.3 × 10^5^	1.9 × 10^−2^	58 nM	70 s
Ce-2	*B. malayi*	4.8 × 10^5^	2.2 × 10^−4^	0.5 nM	52 min
Ce-1 NHOH	*B. malayi*	2.0 × 10^5^	1.5 × 10^−3^	12 nM	7.9 min
Ce-2 Thr13Ser	*B. malayi*	1.6 × 10^6^	7.8 × 10^−5^	85 pM	2.7 h
Ce-2d	*E. coli*	>LoQ	>LoQ	6 μM	≤1 s
Ce-2	*E. coli*	1.0 × 10^5^	4.8 × 10^−3^	69 nM	2.4 min
Ce-1 NHOH	*E. coli*	>LoQ	>LoQ	5 μM	≤1 s
Ce-2 Thr13Ser	*E. coli*	2.4 × 10^5^	3.3 × 10^−4^	1.3 nM	44 min

iPGM, cofactor-independent phosphoglycerate mutase; LoQ, limit of quantitation.

The SEM and % error are reported in Table S5.

### Substitution mutagenesis elaborates ipglycermide side-chain selectivity at *C. elegans* iPGM

The original *in vitro* selection yielding ipglycermide from trillions of possible cyclic peptides resulted in only two sequences, differing at a single position, Tyr7 (Ce-2) or His7 (Ce-1), as shown in Figure 1*A* (18). Given this restricted structure–activity relationship, possibly resulting from the practical limitations in testing all possible sequence combinations, we elected to broadly investigate side-chain substitutions on ipglycermide using a variation of the original discovery approach (27). The Random Non-standard Peptide Integrated Discovery (RaPID) platform uses a combination of mRNA display coupled to *in vitro* translation, allowing incorporation of noncanonical amino acids *via* genetic code reprograming. Initiating translation with an electrophilic amino acid, *N*-chloroacetyl tyrosine, *in lieu* of methionine, facilitates macrocyclization *via* thiol-mediated nucleophilic substitution by cysteine subsequently incorporated within sterically permissible sequence positions (17). Here, the remaining proteinogenic amino acids and 4-fluorophenylalanine were used to create 280 single-site substitution analogs that evaluated side-chain variation at each of the 14 amino acid positions of the parent ipglycermide, Ce-2. *C. elegans* iPGM immobilized on magnetic beads was used to enrich the library for high-affinity binders. The frequency of positional substitutions after a single cycle of enrichment was determined by deep sequencing using the cognate reverse-transcribed PCR products and yielded the substitution tolerance.

**Figure 4:**
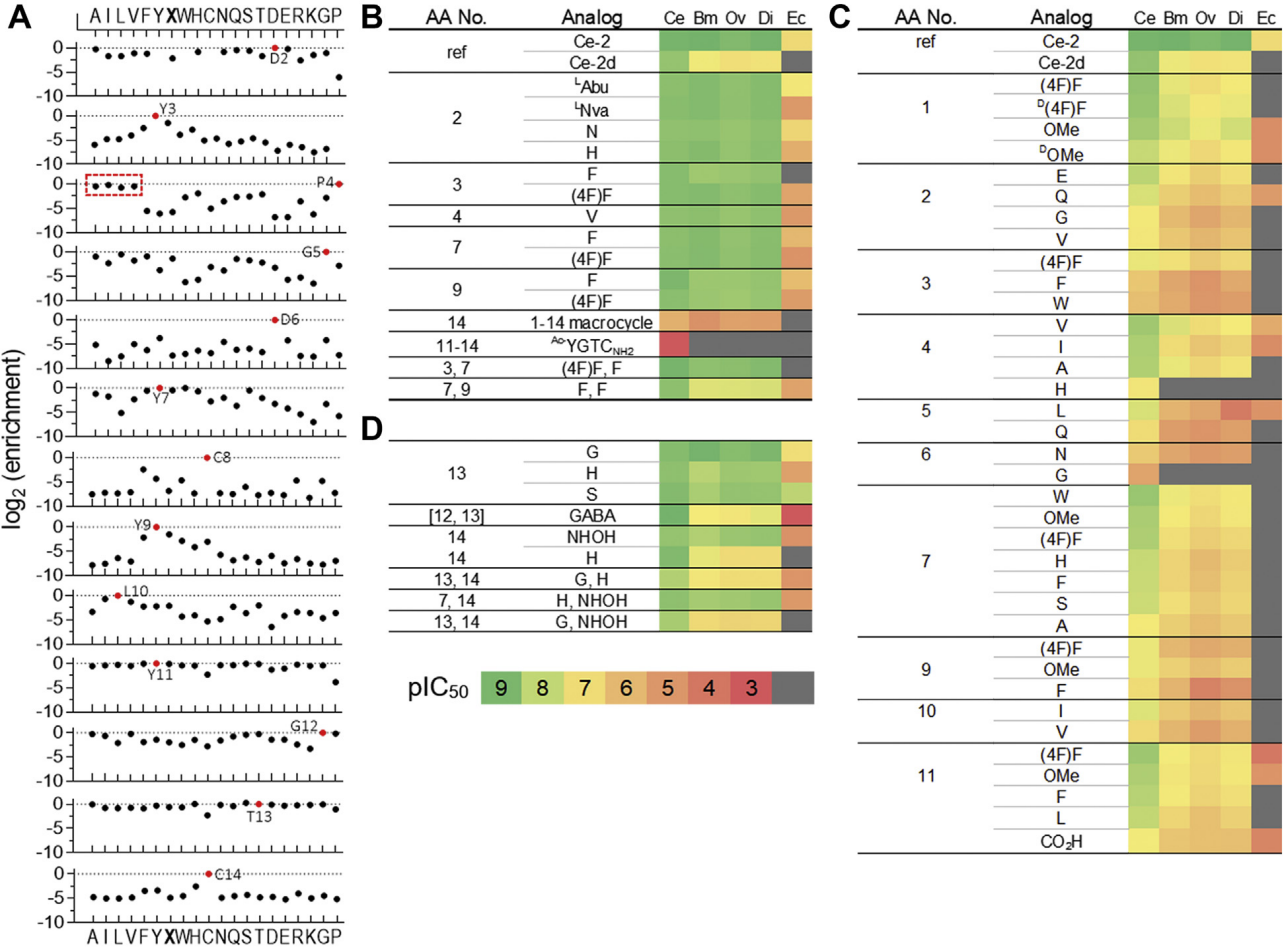
**Affinity enrichment and functional analysis of ipglycermide analogs.***A*, affinity enrichment results from RaPID scanning substitution mutagenesis. Ipglycermide (Ce-2) residues are shown in *red*. *B*, Ce-2 analogs, (*C*) Ce-2d macrocyclic core analogs, and (*D*) Ce-2 metal ion–coordinating analogs tested on *Caenorhabditis elegans*, *Brugia malayi*, *O. volvulus*, *Dirofilaria immitis*, and *Escherichia coli* iPGM. pIC_50_ values are indicated by a *color scale bar*. The deep mutational scanning DNA library contains the WT sequence and single amino acid substitutions. To calculate enrichment scores for each substitution (*black*), the fraction of DNA reads relative to the WT Ce-2 peptide sequence (*red*) was computed. Missing data in Asp2 panel for Y, W, and C indicate insufficient DNA reads were obtained for an enrichment calculation. X = (4F)F, 4-fluoro-phenylanine; [ ], amino acids replaced by indicated moiety; (OMe)F, 4-methoxy-phenylanine; Abu, 2-aminobutanoic acid; GABA, γ-aminobutyric acid; iPGM, cofactor-independent phosphoglycerate mutase; Nva, norvaline; red box, aliphatic amino acids.

To calculate enrichment scores for each mutant (Fig. 4*A*, *black circles*), the fraction of DNA reads relative to the WT Ce-2 peptide sequence (red circles) was computed. Enrichment scores relative to the WT Ce-2 sequence highlight beneficial, neutral, or detrimental single mutations to natural or unnatural (X or (4F)F = 4-fluorophenylalanine) amino acids. The data are plotted as the log2 enrichment *versus* amino acid along the x-axis arranged roughly by hydrophobicity, aromaticity, and polarity/charge. Missing data (Fig. 4*A*, *top panel*: Y, W, C) indicate insufficient DNA reads were obtained for an enrichment calculation. RaPID scanning mutagenesis provided a broad and revealing structure–activity relationship analysis for ipglycermide and was used to guide subsequent SPPS of Ce-2 and Ce-2d analogs for further ortholog selectivity analysis in functional enzyme inhibition assays, binding kinetics, and cocrystal structural determinations.

A salient finding of the RaPID scanning mutagenesis was that no single substitution displayed a major enrichment over Ce-2 for *C. elegans* iPGM, highlighting the power of the RaPID system for identifying high-affinity binders. However, depending on the location, the substitution effect varied significantly at *C. elegans* iPGM, and in cases disproportionately at orthologous iPGMs. To determine this, we prepared analogs by SPPS, referenced to Ce-2 and Ce-2d, to evaluate side-chain structural diversity on the ipglycermide pharmacophore across a panel of iPGM orthologs. In general, SPPS Ce-2 single-position analogs mirror the subtle differences observed in the enrichment data on *C. elegans* iPGM as illustrated in Figure 4*B* and Table S4*A*. However, combining relatively neutral single substitutions into a new analog could diminish functional inhibition, particularly at iPGM orthologs. For example, combining slightly less-favorable phenylalanine substitutions for tyrosine 7 and 9 results in significantly decreased inhibitory activity at the *C. elegans* orthologs (Fig. S5). However, most importantly, significantly decreasing off-rate analogs incorporating a single *C. elegans* iPGM-neutral substitution (*e.g.*, Thr13Ser) enhanced affinity at *E. coli* iPGM (Fig. S5). In general, the results indicate the original discovery of Ce-1/2 likely identified optimal ligands for *C. elegans* iPGM contained in the library and the absence of addition variants, as observed in our focused saturation mutagenesis library, may be a factor related to the sampling size (18).

### Ipglycermide pharmacophore core residues

**Figure 5:**
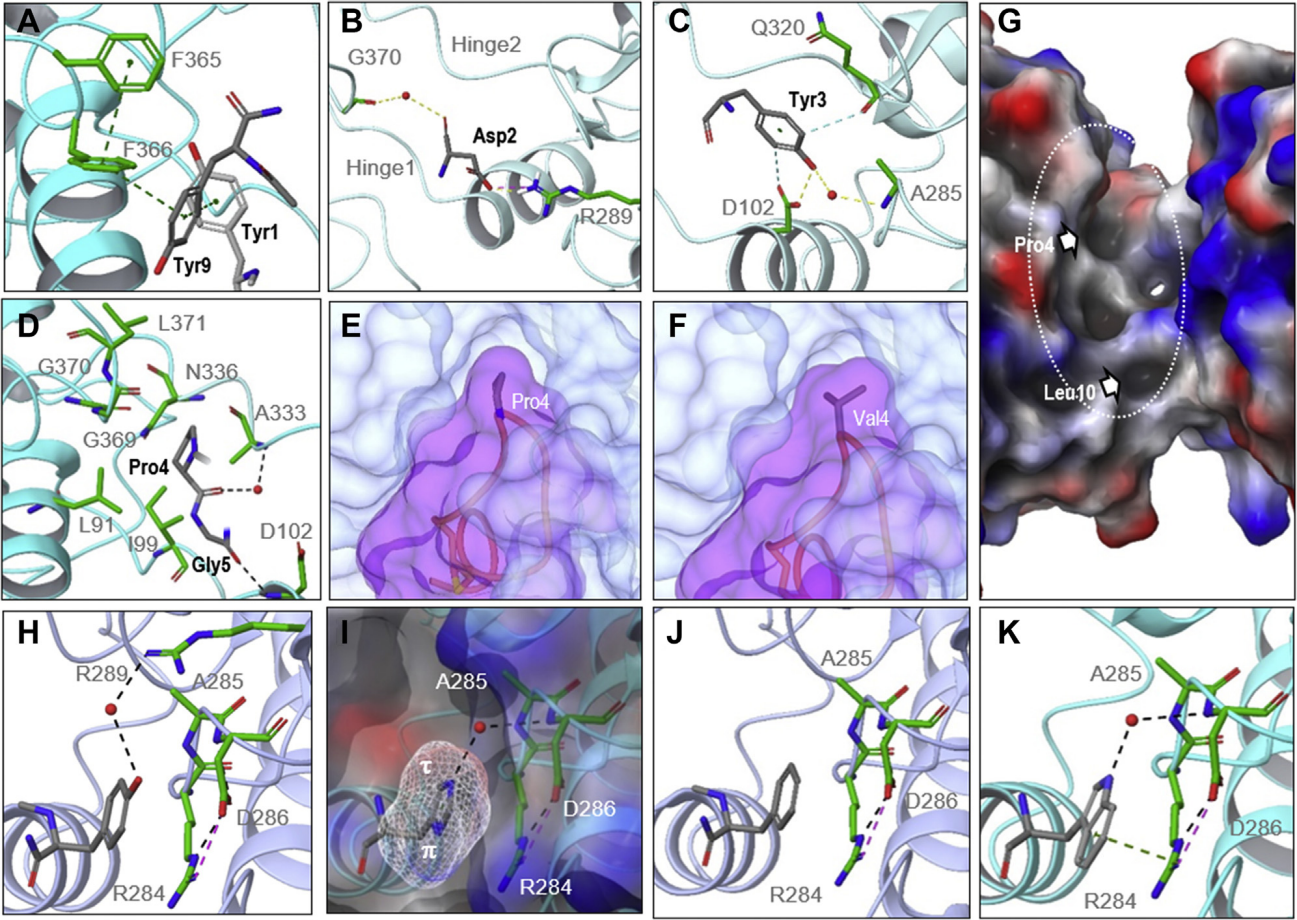
**Ipglycermide core–binding interactions.***A*, aromatic network interaction showing edge-to-face π–π distances (Tyr9–Tyr1, 4.89 Å; Tyr9–F366, 5.01 Å, and F366–F365, 4.72 Å). *B*, Asp2–R289 H-bond and G370–Asp2 α-chain carbonyl water-mediated H-bond. *C*, Tyr3 aromatic interactions with D102 and Q320 carbonyl, and Tyr3 phenolic–A285 amide water-mediated H-bond. *D*, Pro4–Gly5 interactions with hinge1/2 residues and water-mediated α-chain carbonyl–amide H-bond. *E* and *F*, Pro4 binding pocket and modeling of Pro4Val substitution. *G*, Ce iPGM surface topology in the hinge region (*dotted ellipse*). *Arrows* indicted surface depressions accommodating ipglycermide residue 4 and 10 side chains. *H*–*J*, Tyr7, His7, and Phe7 co-crystal structures 5KGN, 7KNF, and 7KNG, respectively. van der Waals mesh shown for His side chain, and the RAD structural motif residues are shown in *green*. *K*, Trp7 modeled using Schrödinger MacroModel and superimposed over the cocrystal structure 7KNF; the water-mediated H-bond (*red sphere*) assumes Trp7 binds similarly as His7 on *Caenorhabditis elegans* iPGM. Ipglycermide amino acids (*gray*) are isolated from the rest of the macrocycle for clarity. The RAD motif is shown as *green* side chains in panels *H*–*K*. *C. elegans* iPGM is represented as *ribbons* colored *ice blue* for the Ce-2d and *cyan* for the Ce-1-hydroxamic acid and Ce-2 Tyr7Phe cocrystal structures, 5KGN, 7KNF, and 7KNG, respectively. iPGM, cofactor-independent phosphoglycerate mutase.

To measure the relative side-chain binding interaction contributions from the ipglycermide core (Fig. 1*B*), we synthesized a series of analogs around Ce-2d rather than the parent ipglycermide (*e.g.*, Ce-1 or Ce-2) to avoid subtle differences from being masked or leveled by the intrinsic high affinity of the parent molecule (Fig. 1*A*). Therefore, using functional enzyme assays (Fig. 4*C*), binding kinetics and crystallographic and modeling data (Fig. 5), we examined the RaPID scanning substitutions in the ipglycermide core framework to explore individual macrocycle side-chain–protein-domain interactions and iPGM ortholog-dependent variations.

#### Cys8 and Asp6

Among the most salient observations from the RaPID scanning mutagenesis are strict dependences on Cys8 and Asp6, central to the structural organization of the ipglycermide macrocyclic. First, macrocycle stability requires the ring-forming thioether linking the Cys8 side chain and Tyr1 *N*-acetyl amide such that analogs devoid of this bond are >10,000-fold less potent in iPGM enzyme assays (18) and formation of the larger 14-member macrocycle through Cys14 results in an inactive analog (Fig. 4*B*, 1–14 macrocycle). Second, in cocrystal structures of ipglycermide–iPGM complexes, Asp6 is buried within the compacted core. Here, Asp6 orchestrates an intramacrocycle H-bonding network principally between the γ-carboxylate and main-chain NH amides of residues 1, 2, 3, and 8 with distances between 1.93 and 2.43 Å (Fig. 1*D* and Table S1). As implied by the RaPID scanning results, Ce-2d analogs replacing Asp6 with glycine or asparagine are essentially inactive (Fig. 4*C*).

#### Tyrosine 1, 3, 7, 9, and 11

Excluding Tyr1, fixed as the N-ClAc-Tyr initiator, RaPID scanning at the ipglycermide Ce-2 tyrosine residues, which comprise >36% of the sequence, displays varying substitution tolerance approximately as follows: Y11 >> Y7 > Y9≈Y3 (Fig. 4*A*). In the functional assays, specific tyrosine analogs, for example, 4-fluoro-Phe or 4-methoxy-Phe (Fig. 4, *B* and *C*), could generally replace either Tyr1, Tyr3, or Tyr9 in Ce-2 (Fig. 4*B*), while differentially impacting the corresponding Ce-2d analog IC_50_ on iPGM orthologs (Fig. 4*C*). However, Tyr7 could be exchanged by all aromatic residues, including histidine found in the only other ipglycermide (Ce-1) selected in the original discovery study (Fig. 4, *B* and *C*). Apart from Cys or Pro, position 11 was indiscriminately substituted consistent with the limited and ill-defined electron density observed around the Tyr11 side chain in the co-crystal structures (Fig. S2). In striking contrast, substitutions at position 7 illustrate highly defined electron density and superposition of Tyr7 (PDB ID: 5KGN), His7 (PDB ID: 7KNG), and Phe7 (PDB ID: 7KNF) as bound to *C. elegans* iPGM (Fig. 2*A* *inset*) juxtaposed to an R284-A285-D286 or RAD motif of the phosphotransferase domain (Fig. 5, *H*–*J*). However, the Ce-2d analogs of His7 and Phe7 displayed lower inhibitory potency on *C. elegans* iPGM than Ce-2d (*i.e.*, Tyr7), with corresponding ∼10-fold decreases in inhibitory potency on the *B. malayi*, *O. volvulus*, and *D. immitis* orthologs. Binding scenarios possibly accounting for these differences include Tyr7 solvation through a water-mediated H-bond network (Fig. S6) absent in the Phe7 structure, and for His7, a potential R284 field clash suggested by residual electropositive surface charge at the *π* imidazole nitrogen (Fig. 5, *H*–*J*). Substitutions of tryptophan or 4-methoxy-tyrosine at Tyr7 displayed similar potency on *C. elegans* and the orthologs (Fig. 4*C* and Table S4*B*). Energy-minimized modeling of the Tyr7Trp side chain indicates the water-mediated H-bond might be replaced or augmented with a *π*-cation interaction involving R284 (Fig. 5*K*).

#### Aspartic acid 2 and glycine 5

In the *C. elegans* iPGM–ipglycermide structures, the Asp2 β-carboxylate participates in a suboptimal backside monodentate H-bond with R289, illustrated in Figure 5*B*, as opposed to an ideal bidentate salt bridge configuration (28) and accounting for Glu enriched in the RaPID scanning analysis (Fig. 4*A*). However, the nonessential nature of this side-chain carboxylate-mediated H-bond is suggested by similar enrichment of Ala at this position (Fig. 4*A*). Based on this observation, Ce-2 analogs incorporating short alkyl side chains (*i.e.*, *L*-α-aminobutyric acid and *L*-norvaline) were prepared and shown to maintain the potency (Fig 4*B* and Fig. S5) and binding kinetics (Table S5) of the cognate Ce-2 ipglycermide inferring Asp2 H-bonding contributes marginally to ipglycermide affinity.

RaPID scanning suggests Gly5 is highly conserved and only leucine approached the baseline enrichment (Fig. 4*A*) value of glycine. However, the Ce-2d Gly5Leu analog (Fig. 4*C*) showed a considerable IC_50_ increase on *C. elegans* iPGM with significant loss of inhibitory potency on the nematode iPGM orthologs, indicating in this case the subtle difference in enrichment equates to substantial binding energy.

#### Hydrophobic interactions and aromatic networks

Interestingly, based on the RaPID scanning results, Pro4 could be substituted for by nonconformationally restrained aliphatic amino acid side chains, confirmed for Pro4Val in a Ce-2 analog (Fig. 4, *A* and *B*). Ce-2d analogs of which Pro4Val retained equivalent Ce-2d nematode ortholog potencies, while Ce-2 Pro4Val displayed a *K*_*D*_ ∼10 lower than Ce-2 on *E. coli* iPGM, due to a significantly slower off-rate (Fig. 4*C* and Tables S4*B* and S5). Pro4 binds in the hinge region of the enzyme. Molecular modeling with valine substituted at this position illustrates how the isopropyl side chain can be accommodated in a hydrophobic depression shaped by hinge two residues A333, A336, hinge one residues I99, L91, and a loop of the phosphatase domain containing G369, G370, and L371 (Fig. 5, *D*–*G*).

The Leu10 amide NH is hydrogen bonded to the amide carbonyl of residue 7 (Fig. 1*D* and Table S1) condensing the proximal extra-macrocyclic residues (Tyr9, Leu10, and Tyr11) to form the ipglycermide core, while the 2-methylpropyl side chain is partially buried in a shallow hydrophobic depression formed by I103, P79 and L82 (Fig. 5*G*). Isoleucine only partly compensated for Leu10 in Ce-2d (25 nM *versus* IC_50_ 2.7 nM, respectively), and was poorly active in the nematode orthologs (Fig. 4*C*).

Tyr1 and Tyr9 of ipglycermide and neighboring enzyme phenylalanine residues illustrate a striking extended aromatic interaction network. Specifically, as seen in Figure 5*A*, Tyr9 bridges aromatic face-edge interactions between Tyr1 and F366 that in turn engages with F365. The aromatic side chain separations fall between 4.72 and 5.01 Å and represent the preferred edge-face conformation observed in protein structures (29).

#### Amino acids linking the ipglycermide core to Cys14

Tyr11, a solvent exposed side chain is readily replaced by most amino acids according to RaPID saturation mutagenesis analysis (Fig. 4*A*) and verified with several Ce-2d substitutions (Fig. 4*C*). This position is therefore well-suited as a site for conjugation to fluorophores, etc. from substitutions such as lysine. Importantly, when residue 11 is not followed by another amino acid, low nanomolar potency is dependent on the Ce-2d carboxamide which orchestrates intra- and intermolecular H-bonds (Fig. 1, *D* and *F*) and without which results in a ∼20-fold *K*_D_ increase on *C. elegans* iPGM and significant loss of iPGM ortholog inhibitory activity (Fig. 4*D*, Ce-2d COOH; Table S4).

From prior Ce-2 truncation studies and a Cys14Ser substitution we concluded the Gly12Thr13 sequence primarily functions to position the Cys14 thiol within coordinating distance of the phosphatase domain transition metal-ion cluster (18). Corroborating these results, RaPID scanning mutagenesis specifies position 12 to be non-aromatic, small neutral amino acids while position 13 has negligible side chain selectivity at *C. elegans* iPGM (Fig. 4*A* and Fig. S5). For example, a Thr13Gly substitution remained equally potent across the iPGM ortholog panel, as did replacement of Gly12Thr13 by a γ-aminobutyric acid linker (Fig. 4*D*). Though importantly, substituting Ser for Thr13 in Ce-2 decreased the *K*_D_ by 10-fold at *E. coli* iPGM through a marked off-rate reduction, demonstrating the context-dependent impact of this position on binding affinity (Fig. 3, *J*–*L*, Table 1). While, in the hydroxamic acid analog framework (Fig. 2*B*), a Thr13Gly substitution substantially reduced inhibitory potency to approximately the Ce-2d iPGM ortholog profile (Fig. 4*D*), indicating a critical function of the beta-hydroxyl to the metal ion coordinating mechanism of the NHOH moiety.

Cys14 is overwhelmingly preferred in the RaPID enrichment (Fig. 4*A*). Notwithstanding we synthesized the Ce-2 Cys14His analog which displayed IC_50_ values on *C. elegans* and *B. malayi* iPGM comparable to Ce-2d (Fig. 4*C*, Table S4). This supports RaPID enrichment results indicating that other proteogenic amino acid side chains normally capable of metal ion coordination cannot, in this circumstance, replace ipglycermide Cys14 in its function (30). Whereas the Ce-1 NHOH analog displays similar pan-ortholog inhibitory potency as Ce-1 and Ce-2 (Fig. 4*B*; Table S4), thus the Thr13-hydroxamic acid moiety acts as rather selective bio-isostere for the Cys14 thiolate of ipglycermide.

## Conclusion

The present work makes advances over our prior study in the following ways, (a) demonstrates through positional substitution mutagenesis that the original affinity selection sampled a small fraction of the theoretical number of high affinity analogous cyclic peptides, (b) elucidates new co-crystal structures defining the Cys14 – transition metal-ion coordination geometry, (c) describes a thiolate isostere using a non-oxidizable hydroxamic acid-beta hydroxyl containing amino acid moiety to replace Cys14, (d) defines the binding kinetics of key ipglycermide analogs across iPGM orthologs, and (e) establishes refined molecular models of the ipglycermide binding mechanism and Asp6-mediated structural compaction of the macrocyclic core.

Featureless or so-called “undruggable” protein surface topologies, for example those lacking clefts and deep pockets typically exploited by small molecule ligands (31), often mediate critical biological functions with therapeutic potential (32, 33). Faced with this scenario we previously used nucleic acid-encoded macrocyclic peptide libraries to identify a complex molecular scaffold with extensive complementarity and high affinity to juxtaposed domain surfaces in iPGM.

Here we elucidated the details underlying a novel binding mechanism at this interface where co-dependent interactions from ipglycermide govern its high affinity for PGMs across a range of nematode species. The conformational dynamics associated with the phosphatase and phosphotransferase iPGM domains facilitate an induced-fit of the rigid macrocyclic core (residues 1–11) primarily through H-bonding with marginal hydrophobic contributions. The imperfect shape complementarity between the ipglycermide core and protein domain surfaces among homologous iPGMs is possibly compensated for by water network interactions (34) analogous to those observed in our *C. elegans* iPGM⋅ipglycermide co-crystal structures (Fig. 1*F* and Table S3). A combination of shape complementarity and H-bonding interactions appears to drive affinity. Interestingly, substitutions removing H-bond D/A and/or increasing hydrophobicity as in Pro4Val, Asp2Nle, Tyr7Phe and Thr13Gly (for Ce-2) remain potent on *C. elegans* iPGM and tend to improve ortholog inhibitory potency, and therefore may possibly be utilized in engineering cellular uptake (35, 36).

We can separate metal ion from core binding elements with the Ce-2d analog. Unaccompanied by the transition metal-ion–coordinating moiety, Ce-2d retains only the bi-domain-spanning macrocyclic core component of ipglycermide. The *K*_D_s for Ce-2d, as determined from SPR biophysical measurements on *C. elegans*, *B. malayi* and *E. coli* iPGMs, span three orders of magnitude. Tethered to the metal ion coordinating Cys14 thiol enhances their respective affinities by ∼100-fold (Table 1). Therefore, metal ion anchoring of the less complementary core binding geometries observed for *B. malayi* and *E. coli* iPGM greatly enhances the overall binding affinity of ipglycermide for these iPGM species. Furthermore, subtle changes at the adjacent amino acid (Thr13 in Ce-2) significantly increases Ce-2 affinity (Ser>Gly>His≈Thr) on *E. coli* iPGM primarily by decreasing *k*_off_ (Table 1 and Table S5).

The resulting *K*_D_ range predicted by binding-energy consolidation (37) from the product of the core *K*_D_ (2–100 nM) and metal-ion–coordinating *K*_D_ (IC_50_ > 500 μM) sequence (^Ac-^YGTC-CONH_2_) is estimated between 10^−14^ and 10^−12^ M. While this theoretical affinity is ∼100-fold higher than the range we observe, the experimental findings are significantly greater than the sum of the interactions and more in line with binding-energy enhancements seen in multisubstrate adduct inhibitors (38). Recently, we have identified purely macrocyclic, nonthiol-containing iPGM inhibitors, suggesting broad potential for inhibitor development (39). However, being devoid of an apparent transition metal-ion–coordinating moiety places the potency of these new cyclic peptides in the range of Ce-2d.

Among all scanned single amino acid mutations, none appeared to appreciably improve binding affinity to *C. elegans* iPGM, over the two sequences originally isolated (18). This finding indicates the most potent of these analogs were among the original library aliquots tested, whereas many equally potent congeners were either not tested or not detected. However, deep mutational scanning experiments extensively filled in structure–activity relationship information absent from the initial discovery selection, delineating pharmacophore positions as invariant (*e.g.*, Asp6) or tolerant of substitution by analogous side chains (*e.g.*, Tyr7), which may be used in future efforts to optimize pharmacokinetic/pharmacodynamic properties. This information is most useful when supported by the crystallographic data. For example, both the solvent exposure and hypervariability of position 11 suggest it would be readily replaceable with linkers, fluorophores, lipids, and so forth, for further analog and probe development.

Cyclic peptides are increasingly leading to the discovery of novel binding surfaces to modulate protein function and elucidate protein dynamics, as recently demonstrated for lysine-specific demethylase one and a missense-mutated K-Ras(G12D)-Raf interaction (40, 41). In the present study, ipglycermide orchestrates a novel bidomain clamp-like property assisted by metal-ion chelation to achieve potent binding affinity across iPGM species orthologs. We anticipate the mechanistic insights detailed here underlying the interaction of ipglycermide with iPGMs will advance our efforts to develop analogs and formulations with bioavailable properties (42).

## Experimental procedures

### Preparation of PGM enzyme constructs

*C. elegans* iPGM, short form (10xHis C-terminal tag), NP_491896.1; *B. malayi* iPGM (10xHis C-terminal tag) AAQ97626.1; *O. volvulus* iPGM (10xHis C-terminal tag), AAV33247.1; *D. immitis* iPGM, (10xHis C-terminal tag) AEA91534.1; *E. coli* iPGM (10xHis C-terminal tag), P37689.1; and *Homo sapiens* dPGM (10xHis C-terminal tag), NP_002620.1 were cloned into pET21a(+) and transformed into BL21(DE3) for *E. coli* expression as described (18). For site-specific incorporation of biotin, the *C. elegans*, *B. malayi*, and *E. coli* iPGM were constructed to have the GLNDIFEAQKIEWHE sequence (26) located between the C terminus and His tag as described in Supporting Information.

### PGM expression and purification

Cultures were taken at a 600 nm absorbance reading between 0.4 and 0.5, cooled to 16 °C for 15 min, and then induced with 0.4 mM IPTG overnight at 16 °C, shaking at 200 rpm. Cell lysate was purified over a HisTrap FF prepacked column eluted with a linear gradient of 50 to 500 mM imidazole, followed by centrifugal concentration of iPGM-containing fractions. Concentrated protein was applied to a HiLoad Sephadex 16/60 prepacked column and eluted with PGM storage buffer (150 mM Tris HCl, 25 mM Mg_2_SO_4_, and 100 mM KCl, pH 8.0). Fractions containing a single, correct molecular weight band were pooled and concentrated. Protein concentration was determined using NanoDrop. Glycerol was added to 20% for cryoprotection, and samples, flash-frozen in liquid nitrogen and stored at −80 °C. Additional details are available in Supporting Information and Protocol S1.

### PGM enzyme assays and IC_50_ determinations

PGM activity was measured as an end-point output assay as described previously (18). Briefly, 4 μl of enzymes was dispensed into white solid-bottom 1536-well plates in a pH 8.0 assay buffer (30 mM Tris HCl, 5 mM Mg_2_SO_4_, and 20 mM KCl). Macrocyclic peptides (5 mM in dimethyl sulfoxide [DMSO]) were added 23 nl/well and incubated for >20 min at ambient temperature, protected from light. 3-Phosphoglycerate in an enolase–pyruvate kinase–coupled enzyme assay buffer (2 μl) was added to enzyme/peptide mixtures and incubated for 5 min at an ambient temperature for all enzymes except *E. coli* iPGM where reactions were incubated at 37^o^C for 15 min, followed by addition of 4 μl Kinase-Glo Plus reagent. Plates were incubated at an ambient temperature for 10 min and then measured by a ViewLux plate reader (PerkinElmer). Concentration response curves were fit, and IC_50_ values, calculated using four-parameter nonlinear regression. For full details, see Supporting Information and Protocol S2.

### *In vitro* biotinylation

iPGM biotinylation reactions were carried out at 100-μM scale in PBS buffer. Free biotin was removed by loading the reaction mixture onto a HiLoad Sephadex 16/60 prepacked column and eluted at 0.5 ml/min with PGM storage buffer. Biotinylation efficiency was measured using the streptavidin conjugation assay (43). Additional details are available in Supporting Information and Protocol S3.

### SPR experiments

SPR experiments, detailed in Protocol S4, were performed using a Biacore S200 instrument at a flow rate between 20 and 40 μl/min, 25 °C using 1X PBP-P as a running buffer. Biotinylated iPGMs were immobilized on a Biotin CAPture (CAP) chip. High-performance kinetic experiments were performed by injecting a series of macrocyclic peptide concentrations (within 10-fold of *K*_D_). Kinetic and affinity data were analyzed using Biacore S200 Evaluation Software. All sensorgrams were fit, after background correction to a 1:1 binding model using the BIAevaluation software as described in Supporting Information.

### RaPID scanning mutagenesis

#### *In vitro* translation system

Flexizyme eFx and tRNAs (${\text{tRNA}}_{CAU}^{fMet}$ and ${\text{tRNA}}_{CAU}^{EnGlu}$) were prepared as described previously (44, 45). CME-activated nonproteogenic amino acids (46) were synthesized as described previously. The *in vitro* translation system has been described elsewhere (27).

#### NNK mutational DNA/mRNA library

Briefly, for the mutational scanning experiment, a single round of RaPID selection was conducted with a single mutant library using NNK codons to introduce amino acid mutations. The constructed DNA library was transcribed into RNA, ligated to a puromycin DNA splint, translated, reverse transcribed, hemagglutinin epitope tag purified, and then subjected to a single round of affinity selection with the target protein *C. elegans* iPGM immobilized on Dynabeads His-Tag (Novex, Thermo Fisher). Isolation, PCR amplification, and sequencing of the cDNA tag of binders allowed the quantitative comparison of single mutant peptides with WT peptides before and after affinity selection as an indication of relative binding affinities.

#### Genetic code reprogramming

The ligated RNA library was translated using an *in vitro* Met-deficient translation system (5 μl scale) with genetic code reprogramming as described previously (47). ^N-ClAc^*D*-Tyr was assigned to the initiation “AUG” codon by adding aminoacylated ^N-ClAc^*D*-Tyr ${\text{tRNA}}_{CAU}^{fMet}$. 4F-Phe was assigned to the elongation “AUG” codon by adding aminoacylated 4F-Phe ${\text{tRNA}}_{CAU}^{EnGlu}$.

#### cDNA–mRNA–peptide fusion library purification

The library was purified using a C-terminal HA tag so that only fully translated sequences entered the affinity selection step and to remove translation system components and unligated RNA. The blocked cDNA-mRNA-peptide fusion library was incubated with 10 μl anti-HA magnetic beads by rotating at 4 °C for 1 h. After washing, bound peptides were eluted twice with 20 μl elution buffer (0.1% acetylated BSA, 0.05% Tween-20, 2 mg/ml HA peptide in tris-buffered saline). The cDNA–mRNA–peptide fusion library obtained after HA purification was used as the “input library” in the following analysis.

#### Affinity selection

*C. elegans* iPGM was immobilized on Dynabeads His-Tag, washed three times with tris-buffered saline (TBS) and Tween 20 (3 μl) on ice, and then incubated with the “input library” by rotating at 4 °C for 30 min. The supernatant containing unbound library members was discarded, and the beads were washed three times (6 h, 12 h, and 6 h) at 4 °C with rotation with 200 μl tris-buffered saline-T. Beads were resuspended in 20 μl 0.1% Triton-X and transferred to a new tube. The sample was heated at 95 °C for 5 min to denature iPGM and the RNA/DNA duplex. The supernatant was recovered to obtain the “output” cDNA of bound library members.

#### Real-Time PCR

The concentration of “input” and “output” DNA was assessed by real-time PCR (LightCycler 96, Roche) using primers listed in Supporting Information. HA purification efficiency and total library recovery for mutational scanning conditions are reported in Table S6.

#### Illumina MiSeq sequencing and enrichment score (E) calculation

Input and output samples were directly amplified by PCR with high-fidelity Phusion polymerase (NEB) using MiSeq primers containing indices required for Illumina sequencing. The first PCR used primers Rd1T7g10M.F70 and HA_Rd2R49c. The second PCR used primers P5XXXXRd1.F57 and Rd2XXXXP7_R52 in which XXXX refers to the specific indices used for each separate sample (see Supporting Information for primer sequences). The MiSeq samples were purified by NucleoSpin Gel and PCR Clean-up (Macherey-Nagel) to remove PCR primers, and exact concentrations were determined using the Qubit dsDNA BR kit (Thermo Fisher). Input and Output library samples were run on the MiSeq (Illumina) platform (single 151 cycle read mode, v3 chip). An enrichment score (E) for each peptide in the library was calculated as published previously (27). Complete details for RaPID scanning mutagenesis are described in Supporting Information.

### Synthesis of ipglycermides

Precyclized peptides were synthesized by standard Fmoc automated SPPS on either a 25 or 100 μmol scale (18, 48). C-terminal acid, amide, or hydroxyl amine peptides were synthesized on Rink Acid, Amide, or Hydroxylamine Wang resin, respectively. Fmoc-Cys(Mmt) was used for differential cysteine deprotection for peptides containing Cys14. Peptide N-termini were N-chloroacetylated using a 0.2 M solution of N-(chloroacetoxy) succinimide (ClAc-NHS, 8 eq. in DMF) by incubating with agitation for 1 h at room temperature (RT). Peptides for Ce-2 hydroxylamine and Ce-2d analogs were cleaved off the resin and deprotected with TFA cocktail upon gentle stirring for 3 h at RT. The reaction mixture was filtered, and the resin was washed with TFA (2× 1 ml). The filtrate containing the peptide was concentrated *in vacuo*, precipitated with ice-cold diethyl ether, dried *in vacuo* and then redissolved in DMSO, and adjusted to the basic pH with addition of triethylamine for thioether macrocyclization. Cys14-containing ipglycermide analogs were cyclized on-resin after selective Mmt deprotection by 4 to 7 washes (5 min each) using a dichloromethane solution containing 5% TFA, 2.5% triisopropylsilane. On-resin thioether macrocyclization was then conducted through the addition of 5% N,N-diisopropylethylamine in DMF at RT overnight. Completion of cyclization was assessed using Ellman’s reagent (5,5'-dithiobis-(2-nitrobenzoic acid)) to test for the presence of free uncyclized thiol groups. Cyclic peptides were cleaved from resin as described previously (18), followed by precipitation and redissolving in DMSO. Peptides were purified by reverse-phase HPLC and identity confirmed by MS (Table S7). Additional experimental details are provided in Supporting Information.

### Crystallography

#### Crystallization and data analysis

Purified apo–*C. elegans* iPGM–10xHis at a concentration of 17.2 mg/ml (0.3 mM) in 150 mM NaCl, 30 mM Tris pH 8.0 was complexed with ipglycermide analogs, Ce-2 Tyr7Phe and Ce-1 C14NHOH (20 mM in DMSO) in a 1:1.5 (protein:peptide) molar ratio mixture, and incubated on ice for 30 min before screening. All crystallization experiments were set up using an NT8 drop-setting robot (Formulatrix Inc) and UVXPO MRC (Molecular Dimensions) sitting-drop vapor diffusion plates at 18 °C. Protein (100 nl) and crystallization solution (100 nl) were dispensed and equilibrated against 50 μl of the latter to yield crystals displaying a plate morphology after ∼7 days from the Index Screen HT (Hampton Research) condition D7 for the iPGM–Ce-1 C14NHOH complex and condition D8 containing 3% (w/v) trimethylamine N-oxide for the iPGM–Ce-2 Tyr7Phe complex. Cryoprotectant-preserved samples were harvested directly from the drop and stored in liquid nitrogen. X-ray diffraction data for iPGM–Ce-1 C14NHOH were collected at the Advanced Photon Source IMCA-CAT beamline 17-ID using a Dectris Pilatus 6M detector. Diffraction data for iPGM–Ce-2 Tyr7Phe were collected at the National Synchrotron Light Source II beamline 17-ID-1 (AMX) using a Dectris Eiger 9M detector. Intensities were integrated using XDS (49, 50) *via* autoPROC (51), and the Laue class analysis and data scaling were performed with Aimless (52), which indicated that the highest probability Laue class was 2/*m*. Diffraction data from two crystals were scaled together for iPGM–Ce-1 C14NHOH to improve the multiplicity. Structure solution was conducted by molecular replacement using a previously determined isomorphous structure (PDB ID: 5KGN) as the search model (18). Structure refinement and manual model building were conducted with Phenix (53) and Coot (54), respectively. Disordered side chains were truncated to the point for which electron density could be observed. Structure validation was conducted with MolProbity (55). Relevant crystallographic data are provided in Table S2. X-ray crystal structures were prepared using the protein preparation wizard of Maestro (Schrödinger, LLC) by adding hydrogen atoms and any missing side chains. A rigid energy minimization of only the hydrogens atoms was performed using the OPLS3e force field (56). Polder omit maps were calculated with Phenix (57). We modeled alternate side chains at positions Pro4 and Tyr7 of ipglycermide using the 5KGN structure and restrained energy minimization (58) and rendered using either CCP4MG or Maestro’s electrostatic potential surface. Superposition of apo and bound structures were superimposed over the phosphatase region (A/96-196 and A/169-338). The distances varying between the remaining amino acids were measured using CCP4MG. For additional details, see Supplementary Methods. Coordinates and structure factors were deposited to the Worldwide Protein Data Bank with the accession codes 7KNF (iPGM⋅Ce-1 NHOH) and 7KNG (iPGM⋅Ce-2 Tyr7Phe).

## Data availability

The mass spectrometry proteomics data have been deposited to the ProteomeXchange Consortium (http://proteomecentral.proteomexchange.org) *via* the PRIDE partner repository (59) with the dataset identifier PXD024074 and DOI 10.6019/PXD024074.

## Supporting information

Data supporting footnote 2 are found in Supplementary Proteomics Tables 1–6. Primer sequences used in the PCR assembly of the DNA library are listed in Table S8. This article contains Supporting Information (60, 61, 62, 63).

## Conflict of interest

The authors declare that they have no conflicts of interest with the contents of this article.

## Supporting information

Supplementary Methods, Figures S1–S6, Tables S1–S8 and Protocols S1–S4

Supplementary Proteomics Tables 1–6

## Footnotes

† In the text, the single-letter amino acid convention is used to denote protein residues and sequences, whereas the three-letter code is used in descriptions of the peptide macrocycles.

§ We initially concluded *Escherichia coli* iPGM was potently inhibited by Ce-2d because of a mislabeled enzyme lot. The lot of enzyme labeled as “Ec iPGM” for *E. coli* iPGM was actually “Ce iPGM” (*i.e.*, *Caenorhabditis elegans* iPGM), as later determined when *E. coli* iPGM prepared in our laboratory (J.I.) was not significantly inhibited by Ce-2d. MS-based sequence analysis subsequently confirmed the original sample was indeed *C. elegans* iPGM, not *E. coli* iPGM (Fig. S1). Repeated attempts to correct this in the published article (18) were unsuccessful.
