# Supplementary material for: Structure–activity relationship of ipglycermide binding to phosphoglycerate mutases
Source: J Biol Chem. 2021 Apr 1;296:100628. doi: 10.1016/j.jbc.2021.100628 (PMC8113725; doi:10.1016/j.jbc.2021.100628)
Supplement: Supplementary Methods, Figures S1–S6, Tables S1–S8 and Protocols S1–S4 [file mmc1.pdf]

Structure-activity relationship of ipglycermide binding to phosphoglycerate mutases

Mareike Wiedmann[1†], Patricia K. Dranchak[2†], Mahesh Aitha[2], Brian Queme[2], Christopher D. Collmus[2], Maithri M. Kashipathy[4], Liza Kanter[2], Laurence Lamy[2], Joseph M. Rogers[1], Dingyin Tao[2], Kevin P. Battaile[3], Ganesha Rai[2], Scott Lovell[4], Hiroaki Suga[1]*, James Inglese[2,5]*

[1]Department of Chemistry, Graduate School of Sciences, The University of Tokyo, 7-3-1 Hongo, Bunkyo-ku, Tokyo 113-0033, Japan
[2]National Center for Advancing Translational Sciences, National Institutes of Health, Rockville, MD, USA
[3]IMCA-CAT Advanced Photon Source, Argonne National Laboratory, Argonne, IL, USA
[4]Protein Structure Laboratory, Structural Biology Center, University of Kansas, Lawrence, KS, USA
[5]National Human Genome Research Institute, National Institutes of Health, Bethesda, MD, USA

§Current Addresses: M.W., Bayer Pharmaceuticals, R&D Division, Wuppertal, Germany; L.K., University of Florida College of Dentistry, Gainesville, FL 32610, US; J.M.R., Department of Drug Design and Pharmacology, University of Copenhagen, Copenhagen 2100, Denmark; K.P.B, New York Structural Biology Center, Beamline (NYX) at Brookhaven National Laboratory NSLS-II Synchrotron Brookhaven Avenue, Upton, NY 11973, US

†Contributed equally to this work
*Corresponding authors; J.I. (jinglese@mail.nih.gov) and H.S. (hsuga@chem.s.u-tokyo.ac.jp)

Table of contents

Supplementary experimental procedures pgs. S1−S12
Supplementary figures pgs. S13−S19
Supplementary tables pgs. S20−S30
Supplementary protocol tables pgs. S31-S37

Supplementary experimental procedures

Preparation of PGM enzyme constructs

PGM enzymes *C. elegans* iPGM, long form (10xHis C terminal tag), NP_491896.1; *B. malayi* iPGM (10xHis C terminal tag) AAQ97626.1; O. *volvulus* iPGM (10xHis C–terminal tag), AAV33247.1; *Dirofilaria immitis* iPGM, (10xHis C–terminal tag) AEA91534.1; *Homo sapiens* dPGM (10xHis C–terminal tag), NP_002620.1; *E. coli* iPGM (10xHis C terminal tag), P37689.1; and *E. coli* dPGM (10xHis C–terminal tag), P62707.2 were described (1). For site-specific incorporation of biotin, the *C. elegans, B. malayi*, and *E. coli* iPGM were constructed to have the GLNDIFEAQ**K**IEWHE sequence (2) located between the C-terminus and His tag. The sequence encoding the biotinylation substrate sequence (underlined) followed by a thrombin cleavage site (bold) for optional His-tag removal is as follows: GGTCTGAACGACATCTTCGAGGCTCAGAAAATCGAATGGCACGAA**TTAGTGCCTCGCGGAAG**CGCTGCAGGG. Hind3 or Xho1 overhangs were added on either side to allow cloning into *C. elegans* or *B. malayi* and *E. coli* iPGM constructs, respectively. The primers used to generate these constructs, where the biotinylation (bio) sequence is underlined, the thrombin cleavage site sequence is in bold, and the restriction sites overhangs are italicized are as follow:

Ce-iPGM-bio forward:	*AGCTT*GGTCTGAACGACATCTTCGAGGCTCAGAAAATCGAATGGCACGAA**TTAGTGCCTCGCGGAAGC**GCTGCAGGG*A*
Ce-iPGM-bio reverse:	*AGCTT*CCCTGCAG**CGCTTCCGCGAGGCACTAA**TTCGTGCCATTCGATTTTCTGAGCCTCGAAGATGTCGTTCAGACC*A*
Bm- and Ec-iPGM-bio forward:	*TCGAG*GGTCTGAACGACATCTTCGAGGCTCAGAAAATCGAATGGCACGAA**TTAGTGCCTCGCGGAAGCG**CTGCAGGG*C*
Bm- and Ec-iPGM-bio reverse:	*TCGAG*CCCTGCAG**CGCTTCCGCGAGGCACTAA**TTCGTGCCATTCGATTTTCTGAGCCTCGAAGATGTCGTTCAGACC*C*

PGM Expression and Purification

Expression pET21a plasmids containing the desired 10XHis-tagged iPGM were transformed into BL21(DE3) *E. coli* competent cells and 50 ul of cell mixture was plated on LB-agar plate containing 50 µg/ml ampicillin. Starter culture (50 ml LB/AMP50) was inoculated with a streak of colony and the culture was allowed shake overnight at 37°C. Four 1 L LB/AMP50 in 2 L 2800 ml flasks were inoculated using 10 ml overnight starter culture and shaken at 30°C, 200 rpm until OD_{600} = 0.4-0.5. Cultures were cooled to 16°C for 15 min, then induced with 0.4 mM IPTG and allowed to shake overnight at 16°C, 200 rpm for protein production. Cells were harvested by centrifugation at 4°C for 10 min at 7000 rpm, the cell pellet was transferred to a plastic bag and stored at -80°C until purification.

The cell pellet was resuspended in 30 ml buffer A (20 mM Na_2HPO_4, 300 mM NaCl, 10 mM imidazole, pH 7.4) and one protease inhibitor cocktail tablet EDTA free (Pierce) was added. The cells were lysed by sonicating six cycles, 45 sec on/ 60 sec off. The resulting mixture containing lysed cells was centrifuged at 4°C for 20 min at 20,000 rpm to separate the insoluble cell debris from soluble proteins. The supernatant solution was loaded on to a pre-equilibrated His Trap FF prepacked column (with 5 ml bed volume) (Cytiva) at a flow rate of 1.5 ml/min. The resin-bound protein was washed with 5 column volumes of buffer A and 5 column volumes of 92 % buffer A and 8 % buffer B (20 mM Na_2HPO_4, 300 mM NaCl, 500 mM imidazole, pH 7.4) to elute weakly bound or non-specifically adhering proteins off the column. Bound proteins were eluted with a linear gradient of 8-100 % buffer B (50-500 mM imidazole). Fractions containing the PGM were identified in the chromatograph (based on A_{280}), concentration estimated using a NanoDrop 1000 Spectrophotometer (NanoDrop, ThermoFisher) and purity determined using denaturing 4-15% SDS-PAGE. Fractions containing PGM were pooled and concentrated to less than 2 ml using 10 kD cut-off Amicon centrifugal concentrators (Millipore Sigma). The concentrated protein was loaded at a 1 ml/min flow rate onto a Hi-Load Sephadex 16/60 prepacked column (bed volume of 120 ml) (Cytiva), and proteins were eluted at a flow rate of 0.5 ml/min with PGM storage buffer (150 mM Tris-HCl, 25 mM Mg_2SO_4 and 100 mM KCl, pH 8.0). Chromatography fractions containing iPGM were identified from A_{280} absorbance and purity determined using non-reducing denaturing 4-15 % SDS-PAGE. Fractions containing a single band at the correct molecular weight were pooled, concentrated using an Amicon centrifugal concentrator, protein concentration determined using NanoDrop (MW 60,595 Da and extinction coefficient (ε) 50,840 $cm^{-1}M^{-1}$ for *C. elegans* iPGM, MW 58,689 D and ε= 38,990 $cm^{-1}M^{-1}$ for *B. malayi* iPGM and MW 57,564 D and ε=36,510 $cm^{-1}M^{-1}$ for *E. coli* iPGM). Glycerol was added (to 20 %) for cryoprotection, aliquoted into 20 µL fractions, flash frozen in liquid nitrogen and stored at -80°C. Also see, **Supplementary Protocol 1**.

Protein Digestion and HPLC-MS analysis

Approximately 25 µg of protein from each sample was used for in-solution digestion using RapidGest (Waters Corporation, Milford, MA) to enhance enzymatic digestion of protein, followed by denature, reduction, alkylation and incubated with trypsin (1:50, trypsin/protein) at 37 ^{0}C for around 12 hours. Acidified tryptic peptides were desalted using an HPLC C18 column on an Agilent 1200 HPLC system (Agilent Technologies, Santa Clara, CA), lyophilized and then re-suspended in 3% ACN, 97.9% water and 0.1% formic acid (FA) buffer for LC-MS/MS analysis. Around 300 ng of the digested samples was loaded into Agilent LC-MS system comprised of a 1200 LC system coupled to a 6550 QTOF via an HPLC Chip Cube interface. Agilent Polaris-HR-Chip-3C18 chip (360 nL, 180 Å C18 trap with a 75 µm i.d., 150 mm length, 180 Å C18 analytical column) was used for the peptide capture and separation prior to MS analysis. Elution of peptides from the analytical column was performed using a gradient starting at 97% A (A: 99.9% water, 0.1% FA) at 300 nL/min. The mobile phase was 3–10% B for 2 min, 10–35% B for 16 min, 35–99% for 1 min, and maintained at 99% B (B: 90% ACN, 9.9% water, 0.1% FA) for 4 min, followed by re-equilibration of column with 3% B for 6 min. Data dependent (autoMS2) MS acquisition was performed by an Agilent 6550 QTOF at 2 GHz. Precursor MS spectra were acquired from m/z 315 to 1700 and the top 6 peaks were selected for MS/MS analysis. Product scans were acquired from m/z 50 to 1700 at a scan rate of 3 spectra per second. A medium isolation width (~4 amu) was used, and a collision energy of slope 3.6 V/100 Da with a 2.9 V offset was applied for fragmentation. A dynamic exclusion list was applied with precursors excluded for 0.50 min after two MS/MS spectrum was acquired.

Database searching

All the LC-MS/MS raw data were converted to Mascot generic format (.mgf) by Agilent MassHunter Qualitative Analysis B.07.00. Mascot version 2.6.2 was used to search against either the Swiss-Prot database (2020.06) which consists of 563,972 entries, or NCBI protein database (2021.01) which consists of 345,670,150 entries for peptide sequence assignments using the following parameters: precursor ion mass tolerance of 50 ppm and a fragment ion mass tolerance of 0.2 Da. Peptides were searched using fully tryptic cleavage constraints, and up to two internal cleavage sites were allowed for tryptic digestion. Fixed modifications consisted of carbamidomethylation of cysteine. Variable modifications that were considered were oxidation of methionine residues. Protein identification cutoff was set with protein Mascot score >100 along with manual confirmation (**Supplementary Proteomics Tables 1-6**). The mass spectrometric raw data files and associated search results of the six samples have been deposited to the ProteomeXchange Consortium via the PRIDE partner repository(3) with the dataset identifier PXD024074.

PGM enzymatic assays and IC_{50} determination

Inhibition of phosphoglycerate mutase activity in the presence of macrocyclic peptides was measured as an end-point output assay as previously described (1). Briefly, 4 µl enzymes were dispensed into white solid-bottom medium bind 1536-well plates (Greiner Bio-One) in a pH 8.0 assay buffer (30 mM Tris-HCl, 5 mM Mg_2SO_4 and 20 mM KCl) with the BioRaptor FRD (Beckman Coulter). Assay buffer with no enzyme was added to column 1 of each plate as a control. Macrocyclic peptides were prepared at 5 mM in DMSO when material allowed or prepared at the highest concentration for a minimum of 15 ul volume and added to enzymes at 23 nl per well transferred by a Pin tool (Wako Inc.) in a 16-pt, 1:3 titration in duplicate spanning a concentration range of 1.3 pM to 19.2 µM along with DMSO and a titration of Ce2 and Ce2d

positive controls from top concentration 3.8 μM to 0.27 pM in columns 2-4 of each plate. Enzymes were incubated with peptide for >20 min at ambient temperature, protected from light. 2 μl of 3-phosphoglycerate in a coupled enzyme assay buffer (includes enolase and pyruvate kinase) was added to enzyme/peptide mixtures with BioRaptor FRD and reactions were incubated for 5 min at ambient temperature, protected from light for all enzymes except *E. coli* iPGM where reactions were incubated at 37°C for 15 min, followed by addition of 4 μl Kinase-Glo Plus reagent (Promega Corporation). Plates were incubated at ambient temperature for 10 min then measured by a ViewLux plate reader (PerkinElmer). See also **Supplementary Protocol 2**. Concentration response curves were fit and IC_{50} values calculated using nonlinear regression log(inhibitor) vs. response -- Variable slope (four parameters) in Prism (GraphPad Software).

In vitro Biotinylation

iPGM biotinylation reactions were carried out on 100 μM scale in PBS buffer (4). Accordingly, 5 μL of 1M $MgCl_2$, 20 μL 50 μM *E.coli* BirA (35,312 Da; purified using an expression plasmid provided by Dr. Tom Miller, (5), 20 μL 100 mM ATP and 3 μL 50 mM D-biotin were added to the 100 μM iPGM containing a C-terminal biotinylation sequence in 952 μL of PBS. The reaction mixture was incubated at 37°C or 30°C for *C. elegans* iPGM with rocking for 1 hr followed by the addition of a second aliquot of 20 μL 50 μM *E. coli* BirA and 3 μL 50 mM D-biotin and incubated overnight at 16 °C. The reaction mixture was loaded at 1 ml/min onto a Hi Load Sephadex 16/60 prepacked column (Cytiva) (bed volume of 120 ml), and proteins were eluted at 0.5 ml/min with PGM storage buffer (150 mM Tris-HCl, 25 mM Mg_2SO_4 and 100 mM KCl, pH 8.0). Chromatography fractions containing iPGM were identified from A_{280} absorbance and purity determined using denaturing 4-15 % SDS-PAGE. Fractions containing iPGM were pooled together and concentrated using an Amicon centrifugal concentrator. Protein concentration was determined from the appropriate ε using a NanoDrop as described above. For cryo-protection 20 % glycerol was added, aliquoted in to 20 μL fractions, flash frozen in liquid nitrogen and stored at -80°C.

Efficiency of biotinylation was measured using the streptavidin conjugation assay. To achieve this, biotinylated enzyme (50 μM in 5 μL) was incubated with 10 μL of 16.67 μM streptavidin Alexa Flour 488 dye conjugate for 10 mins. Five μL 4x SDS-loading dye was added to the mixture and 1 μL per gel was analyzed on two 4-15 % SDS-PAGE at 180 V for 35 mins. One gel was stained with Coomassie blue and the second was transferred to PVDF membrane, imaged using a with Typhoon using the Alexa 488 dye. For further details see, **Supplementary Protocol 3**.

SPR experiments

SPR experiments, detailed in **Supplementary Protocol 4**, were performed using a Biacore S200 (GE Healthcare) instrument at a flow rate between 20-40 μL/min, 25°C using 1X PBS-P (PBS + 0.5% Surfactant P20 (Tween 20)) as a running buffer. Biotinylated iPGMs were immobilized on a Biotin CAPture (CAP) chip (Cytiva) equilibrated with running buffer for at least 2h subsequent to the attachment of ssDNA-streptavidin to the ssDNA oligomer surface by injection onto the CAP chip at a 2 μL/min flow rate for 300 sec (~3500 RU). Next, the biotinylated iPGMs (30-40 μg/ml) were immobilized onto the surface at a 10 μL/min flow rate for 1-2 mins (~1000 RU) while buffer alone was flowed through the reference channels. High performance kinetic experiments were performed by injecting a series of macrocyclic peptide concentrations (within 10-fold of K_D). The CAP chip was regenerated before the next experiment by

injecting 6M guanidine HCl and 0.25 M NaOH (according to the product manual). Injection start and end points were removed in the Evaluation Software to eliminate the injection spikes. Kinetic and affinity data was analyzed using the Biacore S200 Evaluation Software (GE Healthcare). Figures were generated using the GraphPad Prism7 plotting software. All sensorgrams were fitted, after background correction to a 1:1 binding model using the BIAevaluation based on equations 1 and 2, where A = [ipglycermide], B[0]= R_{max}, and AB[0] = 0.

$$\frac{dAB}{dt} = k_a AB - k_d AB \qquad \textbf{(eq. 1)}$$

$$\frac{dAB}{dt} = -(k_a AB - k_d AB) \qquad \textbf{(eq. 2)}$$

In cases where steady-state binding was measurable, the equilibrium binding affinity response value (R_{eq}) was plotted versus the ipglycermide concentration and fitted with a hyperbolic single-state binding **equation 3**, where R_{max} equals the sensorgram response at saturating ipglycermide concentrations.

$$R_{eq} = R_{max} \left\{ [ipglycermide] \Big/ ([ipglycermide] + K_D) \right\} \qquad \textbf{(eq. 3)}$$

Affinity Selection

Preparation of flexizyme eFx, tRNAs, activated amino acids and the in vitro translation system. Flexizyme eFx and tRNAs ($tRNA^{fMet}_{CAU}$ and $tRNA^{EnGlu}_{CAU}$) were prepared as described previously (6,7). CME-activated non-proteogenic amino acids (8) were synthesized as described previously. The *in vitro* translation system has been described elsewhere (7,9).

Testing the fidelity of the incorporation of two non-proteogenic amino acids during in vitro translation using a model peptide and Maldi-TOF-MS detection: To test for correct incorporation and translation of initiator (N-ClAc-D-Tyr)[9] and elongator (4F-Phe) non-proteogenic amino acids aminoacylated with $tRNA^{fMet}_{CAU}$ and $tRNA^{EnGlu}_{CAU}$ respectively, a DNA model sequence was translated using the previously described in vitro translation system on a 2.5 μL translation scale with aminoacylated non-proteogenic amino acids (50 μM final concentration). Due to the presence of a UAG stop codon in the DNA sequence, release factor 1 (RF1) was added to the translation reaction to release the translated peptide from the ribosome after translation. The translation was conducted in platinum tubes for 30 min at 37 °C. The translated mixture was then purified by C18 C-tip (Wako), eluted with 80% MeCN and 0.5% AcOH and then spotted onto a Maldi plate with a 50% saturated solution of α-cyano-4-hydrocinnamic acid. Translation and incorporation efficiency were evaluated by Maldi-TOF-MS analysis on a positive reflector mode (UltraFlex, Bruker).

DNA model sequence: ATGTGGCGCTTTTGCCCGTATTGCGCGAGCATGGGCAGCGGCAGCGGCAGCTAG

Construction of the NNK mutational DNA/mRNA library. Briefly, for the mutational scanning experiment a single round of RaPID selection was conducted with a single mutant library using NNK codons to introduce amino acid mutations. The constructed DNA library was transcribed into RNA, ligated to a puromycin DNA splint, translated, reverse transcribed, HA tag purified and then subjected to a single round of affinity selection with the target protein Ce iPGM immobilized on Dynabeads His-Tag (Novex, Thermo Fisher). Isolation, PCR amplification and sequencing of the cDNA tag of binders allowed the

quantitative comparison of single mutant peptides with wild-type peptides before and after affinity selection as an indication of relative binding affinities.

The DNA library (5' to 3') that was assembled by PCR (see **Supplementary Table 8** for primer sequences) contained a T7 polymerase binding site, Shine-Dalgarno ribosome binding sequence, the Ce-2 peptide coding region (1), single NNK mutational scanning region beginning with ATG, SerGlySer linker, HA tag (YPYDVPDYA), $(GlySer)_3$ linker and a sequence complementary to the DNA splint used for puromycin ligation. The ATG codon in the Met-deficient translation system was reprogrammed to result in incorporation of $^{N\text{-ClAc}}$*D*-Tyr at the initiator ATG site and incorporation of 4F-Phe at ATG elongator sites. The HA tag was used to purify the library after translation and before affinity selection. ClAc refers to a chloroacetyl group introduced at the N-terminus of the peptide.

Ce2_library_DNA: TAATACGACTCACTATAGGGTTAACTTTAAGAAGGAGATATACATATGGATTATCCTGGTG
ATTATTGTTATCTGTATGGGACTTGTGGTAGCGGCAGCTACCCATACGACGTGCCCGACTAT
GCAGGTTCTGGTTCTGGTTCTTAGGACGGGGGGCGGAAA
Ce-2_library_peptide: ($^{N\text{-ClAc}}$*D*-Tyr)DYPGDYCYLYGTCGSGSYPYDVPDYAGSGSGS (with Met reprogrammed to $^{N\text{-ClAc}}$*D*-Tyr)
Ce-2_DNA: ATGGATTATCCTGGTGATTATTGTTATCTGTATGGGACTTGTGGT
Ce-2_peptide: ($^{N\text{-ClAc}}$*D*-Tyr)DYPGDYCYLYGTCG

A site-saturation mutagenesis library was assembled using Phusion polymerase (NEB) by primer extension and PCR which allowed substitution of 19 canonical amino acids (apart from Met) as well as one non-canonical amino acid (4F-Phe) all at once. It allowed the fast characterization of structure-binding affinity relationships of the peptide sequence. Each DNA primer contained a single "NNK" codon at different locations in the Ce-2 peptide. The library was designed in such a way that it contained DNA sequences featuring an NNK single mutation at every position along the Ce-2 coding sequence. Upon translation, single mutations for all 19+1 amino acids were obtained.

All amino acid positions apart from the "ATG" initiation amino acid position (required for cyclization) were randomized and hence 14 different DNA constructs were generated featuring one NNK mutation at each position. Since both elongator Cys residues were randomized, mutation of each Cys (required for cyclization) resulted in the respective smaller or larger macrocycle.

The library was assembled using Phusion polymerase (NEB) and purified by phenol: chloroform: isoamyl alcohol (PCI) extraction and ethanol precipitation. The assembled Ce-2 mutant DNA library was subsequently transcribed into RNA using T7 RNA polymerase for 3 h at 37°C. The RNA library was purified by PCI extraction and isopropanol precipitation before diluting the library to 10 μM in dH_2O. A puromycin-PEG-DNA splint was ligated to the RNA library using T4 RNA ligase for 30 min at 25°C. The ligated library was purified by PCI extraction and ethanol precipitation before diluting the puromycin-ligated RNA library to 5 μM in dH_2O.

In vitro translation with genetic code reprogramming and reverse transcription. The ligated RNA library was translated using an in vitro Met-deficient translation system (5 µL scale) with genetic code reprogramming as described previously (9). $^{N\text{-ClAc}}$*D*-Tyr was assigned to the initiation "AUG" codon by adding aminoacylated $^{N\text{-ClAc}}$*D*-Tyr $tRNA^{fMet}_{CAU}$. 4F-Phe was assigned to the elongation "AUG" codon by adding aminoacylated 4F-Phe $tRNA^{EnGlu}_{CAU}$. The translation mixture was incubated at 37°C for 30 min and then at 25°C for 12 min. 100mM EDTA pH 8.0 (1 µL) was added and the translated library was dissociated from

the ribosome at 37°C for 30 min while promoting peptide cyclization (reaction of elongation Cys with initiation $^{N\text{-ClAc}}D$-Tyr).

The translated library was reverse transcribed with M-MLV RTase H(-) at 42°C for 1 h using primer (PCR2_rv)_Oligo 20 (see Supplementary Table 8) to add a cDNA tag onto the mRNA barcode to suppress secondary structure formation of the single stranded mRNA, increase barcode stability and to allow facile barcode recovery after affinity selection by dissociation. The result was a cDNA-mRNA-peptide fusion library which was diluted two-fold with blocking solution to give final concentrations of 0.1 % acylated BSA (acBSA) and 0.05 % Tween-20.

HA purification of cDNA-mRNA-peptide fusion library. The library was purified using a C-terminal HA tag so that only fully translated sequences entered the affinity selection step and to remove translation system components and unligated RNA. The blocked cDNA-mRNA-peptide fusion library was incubated with 10 µL anti-HA magnetic beads (Pierce, Thermo Fisher) by rotating at 4°C for 1 h. The beads were washed three times with 10 µL ice-cold TBS-T (0.05% Tween-20 in TBS) and the tube was changed on the last wash. Bound peptides were eluted twice with 20 µL elution buffer (0.1 % acBSA, 0.05 % Tween-20, 2 mg/ml HA peptide (Pierce, Thermo Fisher) in TBS) by rotating at 37°C for 15min and removing the supernatant. The cDNA-mRNA-peptide fusion library obtained after HA purification was used as the "input library" in the following analysis.

Binding to Ce iPGM protein and affinity selection. The target protein Ce iPGM was immobilized on Dynabeads His-Tag for affinity selection. Dynabeads His-Tag (1 µL) were washed three times with TBS-T (3 µL) on ice. Ce iPGM protein was added to the beads and was immobilized by rotating the sample at 4°C for 30 min. The amount of added protein was chosen so that the protein concentration during the later washes with 200 µL buffer was 50 nM. The beads were washed three times (3 µL each). The "input library" was incubated with Ce iPGM immobilized on Dynabeads His-Tag by rotating at 4°C for 30 min. The supernatant containing unbound library members was discarded and the beads were washed three times (6h, 12h, 6h) at 4°C with rotation with 200 µL TBS-T. Beads were resuspended in 20 µL 0.1 % Triton-X and transferred to a new tube. The sample was heated at 95°C for 5 min to denature Ce iPGM and the RNA/DNA duplex. The supernatant was recovered to obtain the "output" cDNA of bound library members.

Real-Time PCR. The concentration of "input" and "output" DNA was assessed by real-time PCR (lightCycler 96, Roche) using primers (PCR2_fw_F48_primer)_Oligo 19 and (PCR2_rv)_Oligo 20 (see Supplementary Table 8). HA purification efficiency and total library recovery for mutational scanning conditions are reported in **Supplementary Table 1**.

(PCR2_fw_F48_primer)_Oligo19: TAATACGACTCACTATAGGGTTAACTTTAAGAAGGAGATATACATATG
(PCR2_rv)_Oligo 20: TTTCCGCCCCCCGTCCTAAGAACCAGAACCAGAACC

Illumina Miseq sequencing and enrichment score (E) calculation. Input and Output samples were directly amplified by PCR with high fidelity Phusion polymerase (NEB) using MiSeq primers containing indices required for Illumina sequencing. The first PCR reaction used primers Rd1T7g10M.F70 and HA_Rd2R49c. The second PCR reactions used primers P5XXXXRd1.F57 and Rd2XXXXP7_R52 in which XXXX refers to the specific indices used for each separate sample.

Rd1T7g10M.F70: CACTCTTTCCCTACACGACGCTCTTCCGATCTTAATACGACTCACTATAGGGTTAACTTTAAGAAGGAGA
HA_Rd2R49c: GACTGGAGTTCAGACGTGTGCTCTTCCGATCTGCACGTCGTATGGGTAGCTGCC
P5XXXXRd1_F57: AATGATACGGCGACCACCGAGATCTACACNNNNNNNNACACTCTTTCCCTACACGAC
Rd2XXXXP7_R52: CAAGCAGAAGACGGCATACGAGATNNNNNNNNGTGACTGGAGTTCAGACGTG

The Miseq samples were purified by NucleoSpin Gel and PCR Clean-up (Macherey-Nagel) to remove PCR primers and exact concentrations were determined using the Qubit dsDBA BR kit (Thermo Fisher). Input and Output library samples were run on the Miseq (Illumina) platform (single 151 cycle read mode, v3 chip). An enrichment score (E) for each peptide in the library was calculated as previously published (10).

Automated solid peptide synthesis

Solid Phase Peptide Synthesis. Fmoc amino acids, resins, and coupling reagents were obtained from commercial sources (CEM Corp., Matthews, North Carolina; Chem-Impex, Wood Dale, Illinois; Sigma-Aldrich; and ChemPep, Wellington, Florida), and used without further purification. N-(chloroacetyl) succinimide (ClAc-NHS) was obtained from A1 BioChemlab (Wilmington, North Carolina) or prepared as described (11).

Macrocyclic peptide precursors were chemically synthesized by standard Fmoc solid phase peptide synthesis (SPPS) on either a 25 or 100 μmol scale using a Syro-I peptide synthesizer (Biotage, Uppsala, Sweden) or Liberty Blue HT12 automated peptide synthesizer (CEM Corp.) using previously published (1,11) and standard manufacturer's procedures (12). C-terminal carboxylate or amide peptides were synthesized on NovaPEG Rink acid or amide resin LL, respectively and C-terminal hydroxamic acid peptides were synthesized from hydroxylamine, polymer-bound on Wang Resin using appropriately protected Fmoc amino acids (6 eq.). Amide couplings on the Syro-I peptide synthesizer were conducted using hexafluorophosphate benzo-triazole tetramethyl uronium (HBTU) and hydroxy-benzotriazole (HOBt) in N,N-dimethylformamide (DMF) and with N,N-diisopropylethylamine (DIPEA) in N-methyl-2-pyrrolidone (NMP), and on the Liberty Blue HT12 using N,N'-diisopropylcarbodiimide (DIC) and ethyl cyanohydroxy iminoacetate (Oxyma) in DMF. Fmoc-protecting groups were removed using 40% piperidine in DMF.

After the final deprotection of the N-terminus, peptides were manually N-chloroacetylated using a 0.2 M solution of ClAc-NHS (8 eq. in DMF) by incubating with agitation for 1 h at room temperature. Peptide resins were then washed with DMF (3x 1.5 ml), dichloromethane (DCM) (3x 1.5 ml) and dried *in vacuo* for 30 min.

Off resin thioether macrocyclization synthesis for peptides containing one Cys residue. The N-chloroacetylated peptides were cleaved off the resin and deprotected using a cleavage cocktail containing 92.5% trifluoracetic acid (TFA), 2.5% triisopropylsilane (TIS), 2.5% 3,6-dioxa-1,8-octanedithiol (DODT), 2.5% H_2O using 1.5 ml per peptide (25 μmol scale). Peptides were incubated for 3h with agitation at room temperature, filtered and the resin was washed with TFA (2x 1 ml). Peptide solutions were concentrated in a Genevac centrifugal evaporator for 1h on the low BP setting at 40°C. Peptides were then precipitated by the addition of ice-cold diethyl ether (5 x 10 ml) and dried in vacuo using a Genevac centrifugal evaporator (low BP setting, room temperature) for 5 min. Peptides were re-dissolved in DMSO (4 ml) and the pH was raised to > pH 8 using triethylamine. Thioether macrocyclization took place over the course of 1 h with agitation. Completion of macrocyclization was assessed by MS analysis. The DMSO-peptide solution was neutralized by addition of TFA and cyclic peptides were subsequently purified by a HPLC system using the preparative methods described below. Final peptides were lyophilized then taken up in DMSO to concentration between 5-20 mM for subsequent characterization and study.

On resin thioether macrocyclization synthesis for C-terminal hydroxamic acids and peptides containing multiple Cys residues. The monomethoxytrityl (Mmt) protecting group on Cys8 was selectively

removed from the resin-bound N-chloroacetylated peptides by 4-7 washes (5 min each) using a DCM solution containing 5 % TFA and, 2.5% TIS. Mmt deprotection was complete when the solutions went from orange to colorless. Peptides were washed with DCM (3x 1.5ml) and DMF (3x 1.5 ml). On-resin thioether macrocyclization was subsequently initiated by addition of 5% DIPEA in DMF (1.2 ml per peptide) at room temperature overnight. Completion of cyclization was assessed using Ellman's reagent to test for the presence of free uncyclized thiol groups. Cyclic peptides were then cleaved off the resin as described previously, followed by precipitation and re-dissolving in 4 ml DMSO.

Peptides were purified by reverse-phase HPLC using one of the following methods: A Shimadzu prominence LC-20AP system with Merck Chromolith Prep column (100mm x 25mm) running a 7 min. gradient of 10-70% aqueous ACN (0.1 % TFA) with maximum injection volume of 4 mL per sample in a single injection from a 10 ml injection loop operating at a flow rate of 60 ml/min., or a Waters semipreparative HPLC with a Phenomenex Luna C18 (5 µm, 30mm × 75mm) using gradient of 10-70% aqueous ACN (0.1 % TFA) with maximum injection volume of 1.5 mL per sample in a single injection from a 5 ml injection loop operating at a flow rate of 45 ml/min. Fraction collection was triggered by UV detection (220 nm). Peptides were injected as filtered solutions in DMSO with a maximum volume of 4 ml per sample in a single injection using a 10 ml injection loop.

Purity and HRMS analysis. Method 1. Peptide purity was assessed using a Nexera X2 UHPLC system (Shimadzu) fitted with a C18 reverse phase column. A gradient of 10-70 % aqueous ACN (0.1 % TFA) was used to separate peptides while monitoring absorbance at 280 nm. Peptide structures were confirmed based on high resolution mass spectrometry (HRMS) determined on a MALDI-TOF-MS (Autoflex-12S, Bruker Daltonics). Peptide sample dilutions (1 μL peptide sample in 500 μL ACN) were spotted on a MALDI plate using 50 % saturated α-cyano-4-hydrocinnamic acid in 80 % ACN, 0.5 % AcOH. Peptides were investigated using both positive and negative reflector modes between 1-3 kDa.

Method 2. Peptide purity was assessed using 1260 Infinity II HPLC system (Agilent) fitted with a C18 reversed phase column. A gradient of 20-70 % aqueous ACN (0.1 % TFA) was used to separate the peptides while monitoring absorbance at 214/280 nm. Peptide structures and accurate mass were confirmed based on HRMS determined on a HPLC-TOF system (Agilent 6210 TOF system equipped with an Agilent 1200 HPLC system). Peptide sample (10 to 100 µM) were resolved on a reversed phase analytical column (Extend-C18, 3.5 µm, Agilent Technologies) eluted using 4-100% aqueous ACN (0.1% formic acid). MS spectra were acquired from m/z 350 to 1700 or 400 to 3000 at a scan rate of 1 spectrum per second with Profile format. The electrospray ionization (ESI) source parameters were used as follows: gas temperature, 340 °C; gas flow, 10 L/min; nebulizer, 50 psi; fragmentor, 175 V.

Crystallography and modeling

Crystallization and Data Collection. Purified *apo* iPGM from *C. elegans* (iPGM) spanning residues M19 to I539 and harboring a C-terminal hexahistidine tag was concentrated to 17.2 mg/ml (0.3 mM) in 150 mM NaCl, 30 mM Tris pH 8.0. To prepare the peptide complexes a 20 mM peptide stock solution was prepared in DMSO, mixed in a 1:1.5 (protein:peptide) molar ratio and incubated on ice for 30 minutes prior to screening. All crystallization experiments were set up using an NT8 drop-setting robot (Formulatrix Inc.) and UVXPO MRC (Molecular Dimensions) sitting drop vapor diffusion plates at 18 °C. 100 nl of protein and 100 nl crystallization solution were dispensed and equilibrated against 50 µl of the latter. Crystals were obtained from the following conditions. iPGM•Ce-1 NHOH complex (iPGM•Ce-1 NHOH): crystals displaying a plate morphology were observed after approximately 7 days from the Index Screen HT

(Hampton Research) condition D7 (25% (w/v) PEG 3350, 100 mM Bis-Tris pH 6.5). iPGM•Ce-2 Tyr7Phe complex (iPGM•Ce-2 Tyr7Phe): (25% (w/v) PEG 3350, 100 mM HEPES 7.5, 3% (w/v) Trimethylamine N-oxide). A cryoprotectant solution composed of 80% (v/v) crystallant and 20% (v/v) PEG 200 was prepared and 2 µL was layered onto the drops. Samples were harvested directly from the drop and stored in liquid nitrogen for X-ray diffraction data collection.

Structure Solution and Refinement. X-ray diffraction data for iPGM•Ce-1 NHOH were collected at the Advanced Photon Source IMCA-CAT beamline 17-ID using a Dectris Pilatus 6M detector. Diffraction data for iPGM•Ce-2 Tyr7Phe were collected at the National Synchrotron Light Source II (NSLS2) beamline 17-ID-1 (AMX) using a Dectris Eiger2 X 9M detector. Intensities were integrated using XDS (13,14) via Autoproc (15) and the Laue class analysis and data scaling were performed with Aimless (16) which indicated that the highest probability Laue class was 2/*m*. Diffraction data from two crystals were scaled together for iPGM•Ce-1 NHOH to improve the multiplicity. Structure solution was conducted by molecular replacement using a previously determined isomorphous structure (PDB 5KGN (1)) as the search model. Structure refinement and manual model building were conducted with Phenix (17) and Coot (18) respectively. Disordered side chains were truncated to the point for which electron density could be observed. Structure validation was conducted with Molprobity (19). Polder (20) omit maps were calculated with Phenix. Relevant crystallographic data are provided in **Supplementary Table 2.**

Structure rendering and modelling informatics. iPGM•ipglycermide complexes were loaded into Maestro Software (Schrödinger Release 2019-1). The crystal structures were prepared using the protein preparation wizard to assign bond orders, add hydrogens, create zero-order bonds to metals, fill in missing side chains, and generate het states. Default parameters were used for H-bond optimization (sampling of water orientations and use of pH 7.0). Restrained energy minimization was applied to hydrogens only using the OPLS3e force field (21). For 2D ligand interaction maps, waters with fewer than two hydrogen bonds to non-waters were removed. The ligand interaction diagram was exported and graphed with a 2D projection (**Fig. 1f**). We modeled alternate side chains at position Pro4 of ipglycermide using the 5KGN structure; for valine using restrained energy minimization (22). Waters defined in the crystal structure were kept and H-bonds near the mutated residue were reassigned after the restrained energy minimization and graphed using CCP4MG object display surface. For the Tyr7Trp model, restrained energy minimization and H-bond reassignment was graphed using Maestro's electrostatic potential surface. Superposition of Apo and bound structures were superimposed over the phosphatase region (A/96-196 & A/198-338). The distances varying between the remaining amino acids were measured using CCP4MG (23) and graphed in GraphPad Prism.

References

1. Yu, H., Dranchak, P., Li, Z., MacArthur, R., Munson, M. S., Mehzabeen, N., Baird, N. J., Battalie, K. P., Ross, D., Lovell, S., Carlow, C. K., Suga, H., and Inglese, J. (2017) Macrocycle peptides delineate locked-open inhibition mechanism for microorganism phosphoglycerate mutases. *Nat Commun* **8**, 14932
2. Beckett, D., Kovaleva, E., and Schatz, P. J. (1999) A minimal peptide substrate in biotin holoenzyme synthetase-catalyzed biotinylation. *Protein Sci* **8**, 921-929
3. Vizcaino, J. A., Csordas, A., Del-Toro, N., Dianes, J. A., Griss, J., Lavidas, I., Mayer, G., Perez-Riverol, Y., Reisinger, F., Ternent, T., Xu, Q. W., Wang, R., and Hermjakob, H. (2016) 2016 update of the PRIDE database and its related tools. *Nucleic Acids Res* **44**, 11033

4. Fairhead, M., and Howarth, M. (2015) Site-specific biotinylation of purified proteins using BirA. *Methods Mol Biol* **1266**, 171-184
5. Miller, T. W., Amason, J. D., Garcin, E. D., Lamy, L., Dranchak, P. K., Macarthur, R., Braisted, J., Rubin, J. S., Burgess, T. L., Farrell, C. L., Roberts, D. D., and Inglese, J. (2019) Quantitative high-throughput screening assays for the discovery and development of SIRPalpha-CD47 interaction inhibitors. *PLoS One* **14**, e0218897
6. Murakami, H., Ohta, A., Ashigai, H., and Suga, H. (2006) A highly flexible tRNA acylation method for non-natural polypeptide synthesis. *Nat Methods* **3**, 357-359
7. Goto, Y., Katoh, T., and Suga, H. (2011) Flexizymes for genetic code reprogramming. *Nat Protoc* **6**, 779-790
8. Katoh, T., and Suga, H. (2019) Flexizyme-catalyzed synthesis of 3'-aminoacyl-NH-tRNAs. *Nucleic Acids Res* **47**, e54
9. Goto, Y., Ohta, A., Sako, Y., Yamagishi, Y., Murakami, H., and Suga, H. (2008) Reprogramming the translation initiation for the synthesis of physiologically stable cyclic peptides. *ACS Chem Biol* **3**, 120-129
10. Rogers, J. M., Passioura, T., and Suga, H. (2018) Nonproteinogenic deep mutational scanning of linear and cyclic peptides. *Proc Natl Acad Sci U S A* **115**, 10959-10964
11. Kawamura, A., Munzel, M., Kojima, T., Yapp, C., Bhushan, B., Goto, Y., Tumber, A., Katoh, T., King, O. N., Passioura, T., Walport, L. J., Hatch, S. B., Madden, S., Muller, S., Brennan, P. E., Chowdhury, R., Hopkinson, R. J., Suga, H., and Schofield, C. J. (2017) Highly selective inhibition of histone demethylases by de novo macrocyclic peptides. *Nat Commun* **8**, 14773
12. Collins, J. M., Porter, K. A., Singh, S. K., and Vanier, G. S. (2014) High-efficiency solid phase peptide synthesis (HE-SPPS). *Org Lett* **16**, 940-943
13. Kabsch, W. (1988) Automatic-Indexing of Rotation Diffraction Patterns. *J Appl Crystallogr* **21**, 67-71
14. Kabsch, W. (2010) Xds. *Acta Crystallogr D* **66**, 125-132
15. Vonrhein, C., Flensburg, C., Keller, P., Sharff, A., Smart, O., Paciorek, W., Womack, T., and Bricogne, G. (2011) Data processing and analysis with the autoPROC toolbox. *Acta Crystallogr D* **67**, 293-302
16. Evans, P. R. (2011) An introduction to data reduction: space-group determination, scaling and intensity statistics. *Acta Crystallogr D* **67**, 282-292
17. Adams, P. D., Afonine, P. V., Bunkoczi, G., Chen, V. B., Davis, I. W., Echols, N., Headd, J. J., Hung, L. W., Kapral, G. J., Grosse-Kunstleve, R. W., McCoy, A. J., Moriarty, N. W., Oeffner, R., Read, R. J., Richardson, D. C., Richardson, J. S., Terwilliger, T. C., and Zwart, P. H. (2010) PHENIX: a comprehensive Python-based system for macromolecular structure solution. *Acta Crystallogr D* **66**, 213-221
18. Emsley, P., Lohkamp, B., Scott, W. G., and Cowtan, K. (2010) Features and development of Coot. *Acta Crystallogr D* **66**, 486-501
19. Chen, V. B., Arendall, W. B., Headd, J. J., Keedy, D. A., Immormino, R. M., Kapral, G. J., Murray, L. W., Richardson, J. S., and Richardson, D. C. (2010) MolProbity: all-atom structure validation for macromolecular crystallography. *Acta Crystallogr D* **66**, 12-21
20. Liebschner, D., Afonine, P. V., Moriarty, N. W., Poon, B. K., Sobolev, O. V., Terwilliger, T. C., and Adams, P. D. (2017) Polder maps: improving OMIT maps by excluding bulk solvent. *Acta Crystallogr D Struct Biol* **73**, 148-157
21. Harder, E., Damm, W., Maple, J., Wu, C., Reboul, M., Xiang, J. Y., Wang, L., Lupyan, D., Dahlgren, M. K., Knight, J. L., Kaus, J. W., Cerutti, D. S., Krilov, G., Jorgensen, W. L., Abel, R., and Friesner, R. A. (2016) OPLS3: A Force Field Providing Broad Coverage of Drug-like Small Molecules and Proteins. *J Chem Theory Comput* **12**, 281-296

22. Miao, Z., and Cao, Y. (2016) Quantifying side-chain conformational variations in protein structure. *Sci Rep* **6**, 37024
23. Potterton, L., McNicholas, S., Krissinel, E., Gruber, J., Cowtan, K., Emsley, P., Murshudov, G. N., Cohen, S., Perrakis, A., and Noble, M. (2004) Developments in the CCP4 molecular-graphics project. *Acta Crystallogr D* **60**, 2288-2294

Supplementary Figures

- **Supplementary Figure 1.** Proteomics analysis of PGMs
- **Supplementary Figure 2**. Election density maps of Ce-1 NHOH and Ce-2 Y7F
- **Supplementary Figure 3**. Binding modes of Ce-1 NHOH and Ce-2 Y7F
- **Supplementary Figure 4**. Superposition of the *C. elegans* iPGM•Ce-1 NHOH and *C. elegans* iPGM•Ce-2 Y7F structures
- **Supplementary Figure 5.** Concentration response curves for Ce-2 Y7, Y9 combination, Ce-2 D2 and Ce-2 T13 analogs
- **Supplementary Figure 6.** Transferase-ipglycermide water network

Protein sequence coverage: 38% to *B. malayi* iPGM based on GPMI_BRUMA 2,3-bisphosphoglycerate-independent phosphoglycerate mutase **OS=Brugia malayi** GN=ipgm-1 Sample 1 label: **Bm_iPGM** 7.8 mg/ml 345 units/mg

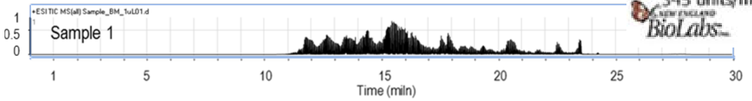

1	MAEAKNR**VCL**	**VVIDGWGISN**	**ETK**GNAILNA	KTPVMDELCV	MNSHPIQAHG
51	LHVGLPE**GLM**	**GNSEVGHLNI**	**GAGR**VVYQDI	VRINLAVKNK	TLVENKHLKE
101	AAERAIKGNG	RMHLCGLVSD	GGVHSHIDHL	FALITALKQL	KVPK**LYIQFF**
151	**GDGR**DTSPTS	GVGFLQQLID	FVNKEQYGEI	STIVGRYYAM	DRDKRWERIR
201	**VCYDALIGGV**	**GEKTTIDKAI**	**DVIK**GR**YAKD**	**ETDEFLKPII**	**LSDEGR**TKDG
251	DTLIFFDYRA	DRMREITECM	GMERYKDLNS	NIKHPKNMQV	IGMTQYK**AEF**
301	**TFPALFPPES**	**HKNVLAEWLS**	**VNGLTQFHCA**	**ETEKYAHVTF**	**FFNGGVEK**QF
351	ANEERCLVVS	PK**VATYDLEP**	**PMSSAAVADK**	VIEQLHMKKH	PFVMCNFAPP
401	DMVGHTGVYE	AAVK**AVEATD**	**IAIGR**IYEAC	KK**NDYILMVT**	**ADHGNAEK**MM
451	APDGSKHTAH	TCNLVPFTCS	SMKYKFMDKL	PDR**EMALCDV**	**APTVLK**VMGV
501	PLPSEMTGQP	LVNEA			

Protein sequence coverage: 12% to *D. immitis* iPGM based on Protein View, AEA91534.1 Sample 2 label: **Di_iPGM** 10 mg/ml 351 units/mg

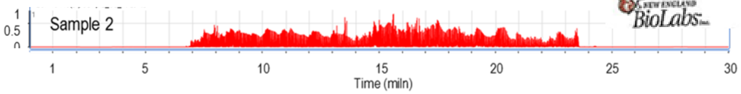

1	MAEAKNRVCL	VVIDGWGISN	ESKGNAILNA	KTPIMDELCA	LNSHPIEAHG
51	LHVGLPEGLM	GNSEVGHLNI	GAGRVVYQDI	VRINLAVKNK	TLVENKHLKE
101	AAERAIKGNG	RIHLCGLVSD	GGVHSHIDHL	FALVTALKQL	KVPQLFIHFF
151	GDGRDTSPTS	GVGFLEQLID	FVNKEQYGVI	ATIVGRYYAM	DRDKRWERIR
201	**VCYDALIAGV**	**GEK**ATIDKAV	DVIKSRYAKD	ETDEFLKPII	LSDEGRTKDG
251	DTLIFFDYRA	DRMREITECM	GMERYKDLKS	DIKHPKNMQV	IGMTQYKAEF
301	TFPALFPPES	HK**NVLAEWLS**	**VNGVTQFHCA**	**ETEK**YAHVTF	FFNGGVEKQF
351	ENEERCLVAS	PK**VATYDLDP**	**PMSSAGVADK**	MIEQLDRKAH	AFVMCNFAPP
401	DMVGHTGVYE	AAVKAVEATD	IAIGRIYEAC	KKNDYILMVT	ADHGNAEKMM
451	APDGSKHTAH	TCNLVPFTCS	SMKFKFMDKL	PDR**EMALCDV**	**APTVLK**VMGL
501	PLPPEMTGKP	VVIEV			

Protein sequence coverage: 38% to *C. elegans* iPGM based on Protein View, GPMI_CAEEL Sample 3 label: **Ec_iPGM** 3.5 mg/ml 44 units/mg

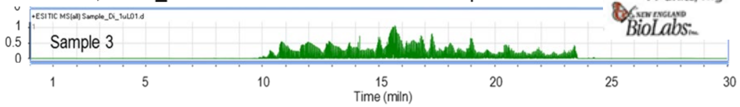

1	MFVALGAQIY	RQYFGRRGMA	MANNSSVANK	**VCLIVIDGWG**	**VSEDPYGNAI**
51	**LNAQTPVMDK**	LCSGNWAQIE	AHGLHVGLPE	GLMGNSEVGH	LNIGAGR**VIY**
101	**QDIVR**INLAV	**KNNKFVTNES**	**LVDACDR**AKN	GNGRLHLAGL	VSDGGVHSHI
151	DHMFALVKAI	KELGVPELYL	HFYGDGR**DTS**	**PNSGVGFLEQ**	**TLEFLEK**TTG
201	YGKLATVVGR	YYAMDRDNRW	ER**INVAYEAM**	**IGGVGETSDE**	**AGVVEVVR**KR
251	**YAADETDEFL**	**KPIILQGEK**G	RVQNDDTIIF	**FDYR**ADRMRE	**ISAAMGMDR**Y
301	KDCNSKLAHP	SNLQVYGMTQ	YKAEFPFKSL	FPPASNK**NVL**	**AEWLAEQKVS**
351	QFHCAETEKY	AHVTFFFNGG	LEKQFEGEER	CLVPSPK**VAT**	**YDLQPEMSAA**
401	**GVADK**MIEQL	EAGTHPFIMC	NFAPPDMVGH	TGVYEAAVK**A**	**CEATDIAIGR**
451	IYEATQK**HGY**	**SLMVTADHGN**	**AEK**MKAPDGG	KHTAHTCYRV	**PLTLSHPGFK**
501	FVDPADRHPA	LCDVAPTVLA	IMGLPQPAEM	TGVSIVQKI	

Protein sequence coverage: 54% to *C. elegans* iPGM based on Protein View, GPMI_CAEEL Sample 4 label: **Ce_iPGM** 5.5 mg/ml 227 units/mg

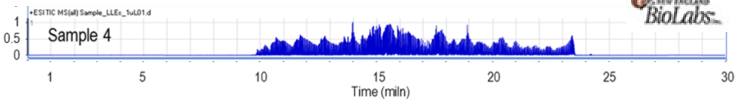

1	MFVALGAQIY	RQYFGR**RGMA**	**MANNSSVANK**	**VCLIVIDGWG**	**VSEDPYGNAI**
51	**LNAQTPVMDK**	LCSGNWAQIE	AHGLHVGLPE	GLMGNSEVGH	LNIGAGRVIY
101	QDIVR**INLAV**	**KNNKFVTNES**	**LVDACDR**AKN	GNGRLHLAGL	VSDGGVHSHI
151	DHMFALVKAI	KELGVPELYL	HFYGDGR**DTS**	**PNSGVGFLEQ**	**TLEFLEK**TTG
201	YGKLATVVGR	YYAMDRDNRW	ER**INVAYEAM**	**IGGVGETSDE**	**AGVVEVVR**KR
251	**YAADETDEFL**	**KPIILQGEK**G	**RVQNDDTIIF**	**FDYR**ADRMRE	**ISAAMGMDR**Y
301	KDCNSK**LAHP**	**SNLQVYGMTQ**	**YKAEFPFKSL**	**FPPASNKNVL**	**AEWLAEQKVS**
351	**QFHCAETEKY**	AHVTFFFNGG	LEKQFEGEER	CLVPSPK**VAT**	**YDLQPEMSAA**
401	**GVADK**MIEQL	EAGTHPFIMC	NFAPPDMVGH	TGVYEAAVK**A**	**CEATDIAIGR**
451	IYEATQK**HGY**	**SLMVTADHGN**	**AEK**MKAPDGG	KHTAHTCYR**V**	**PLTLSHPGFK**
501	**FVDPADRHPA**	**LCDVAPTVLA**	**IMGLPQPAEM**	**TGVSIVQK**I	

Protein sequence coverage: 35% to *E. coli* iPGM based on Protein View, GPMI_ECOHS Sample 5 label: L. Lamy *E. coli* prep

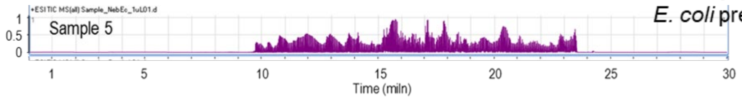

1	MLVSKKPMVL	VILDGYGYRE	EQQDNAIFSA	KTPVMDALWA	NRPHT**LIDAS**
51	**GLEVGLPDRQ**	**MGNSEVGHVN**	**LGAGR**IVYQD	LTRLDVEIKD	R**AFFANPVLT**
101	**GAVDK**AKNAG	KAVHIMGLLS	AGGVHSHEDH	IMAMVELAAE	RGAEKIYLHA
151	FLDGRDTPPR	SAESSLKKFE	EKFAALGKGR	VASIIGRYYA	MDRDNRWDRV
201	EK**AYDLLTLA**	**QGEFQADTAV**	**AGLQAAYAR**D	ENDEFVKATV	IR**AEGQPDAA**
251	**MEDGDALIFM**	**NFR**ADRAREI	TR**AFVNADFD**	**GFAR**KKVVNV	DFVMLTEYAA
301	DIKTAVAYPP	ASLVNTFGEW	MAKNDKTQLR	ISETEKYAHV	TFFFNGGVEE
351	SFKGEDRILI	NSPK**VATYDL**	**QPEMSSAELT**	**EK**LVAAIKSG	K**YDTIICNYP**
401	**NGDMVGHTGV**	**MEAAVKAVEA**	**LDHCVEEVAK**	AVESVGGQLL	ITADHGNAEQ
451	MR**DPATGQAH**	**TAHTNLPVPL**	**IYVGDK**NVKA	VEGGKLSDIA	PTMLSLMGME
501	IPQEMTGKPL	FIVE			

Protein sequence coverage: 41% to *O. volvulus* iPGM based on Protein View, GPMI_ONCVO Sample 6 label: **Ov_iPGM** 3.2 mg/ml 360 units/mg

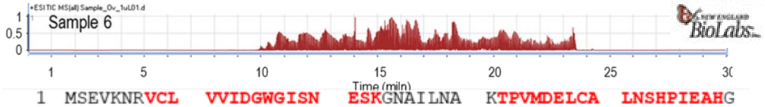

1	MSEVKNR**VCL**	**VVIDGWGISN**	**ESK**GNAILNA	K**TPVMDELCA**	**LNSHPIEAH**G
51	LHVGLPEGLM	GNSEVGHLNI	GAGRVVYQDI	VRINLAVKNK	TLVENKHLKE
101	AAERAIKGNG	RIHLCGLVSD	GGVHSHIDHL	FALITALKQL	KVPQLYIHFF
151	GDGRDTSPTS	GVGFLQQLID	FVNKEQYGEI	ATIVGRYYAM	DRDKRWER**IR**
201	**VCYDALIAGV**	**GEKTTIDKAI**	**DVIK**GR**YAKD**	**ETDEFLKPII**	**LSDK**GRTKDG
251	DTLIFFDYRA	DRMR**EITECM**	**GMER**YKDLKS	DIKHPK**DMQV**	**IGMTQYKAEF**
301	**TFPALFPPES**	**HKNVLAEWLS**	**VK**GLTQFHCA	ETEK**YAHVTF**	**FFNGGVEK**QF
351	ENEERCLVPS	PK**VATYDLEP**	**AMSSAGVADK**	MIEQLNRKAH	AFIMCNFAPP
401	DMVGHTGVYE	AAVK**AVEATD**	**IAIGR**IYEAC	K**KNDYVLMVT**	**ADHGNAEK**MI
451	APDGGK**HTAH**	**TCNLVPFTCS**	**SLK**FKFMDKL	PDR**EMALCDV**	**APTVLK**VLGL
501	PLPSEMTGKP	VVIEV			

Supplementary Figure 1. Proteomics analysis of PGMs. (**top**) Total Ion chromatogram of the sample run by HPLC-Chipcube QTOF. ~300 ng sample was injected for Agilent Polaris-HR-Chip-3C18 chip separation prior to MS/MS analysis. (**bottom**) sequence match (red) to protein database.

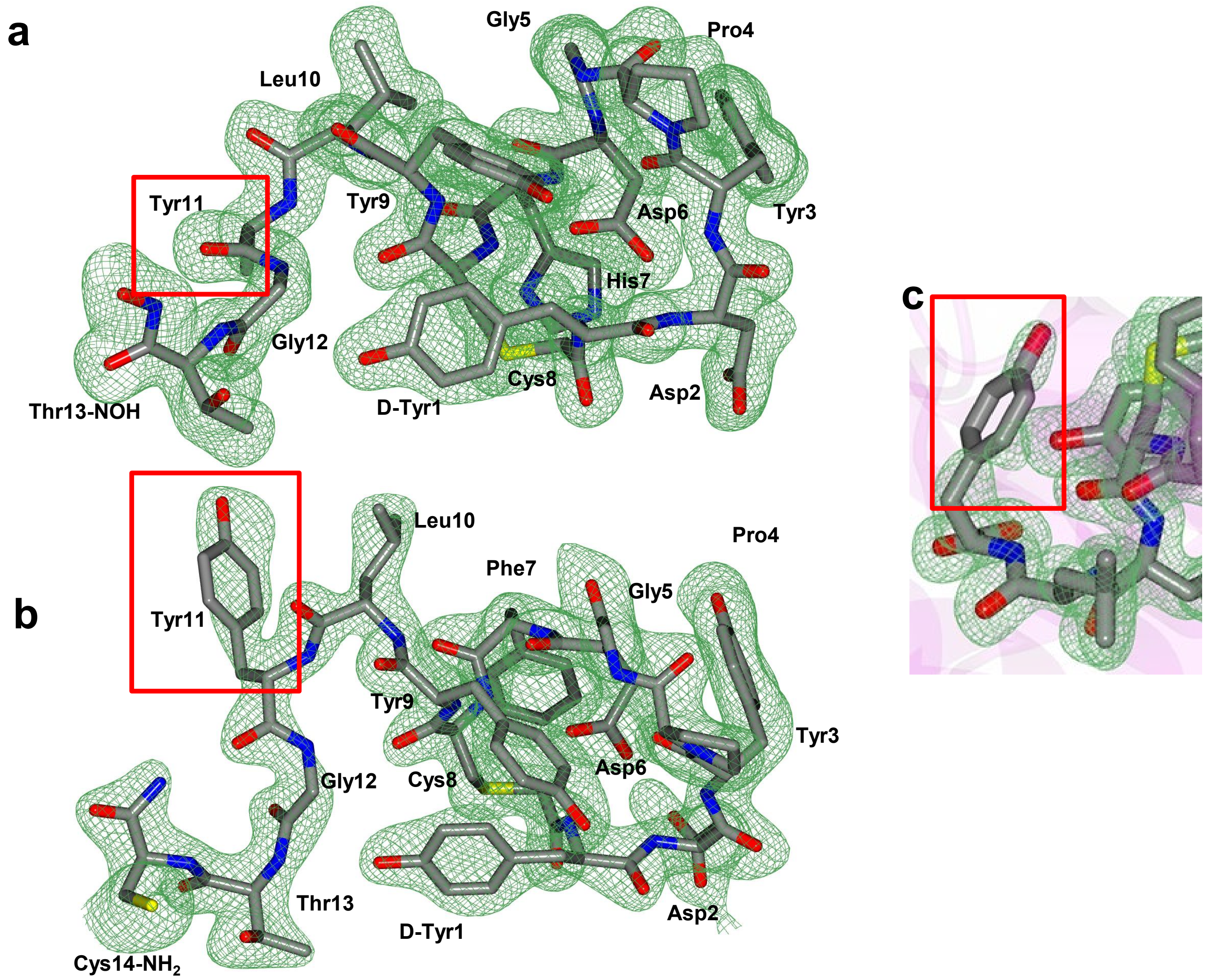

Supplementary Figure 2. Election density maps of Ce-1 NHOH and Ce-2 Y7F. Fo-Fc Polder omit electron density map of Ce-2 Y7F (**a**) and Ce-2 Y7F (**b**) contoured at 3σ. (c) Election density observed at the Tyr11 side chain from the Ce-2d • *C. elegans* iPGM co-crystal structure. Red boxes show lack of election density at Tyr11 in (a), poorly defined election density at Tyr11 in (b) and limited election density at the Tyr11 side chain in (c).

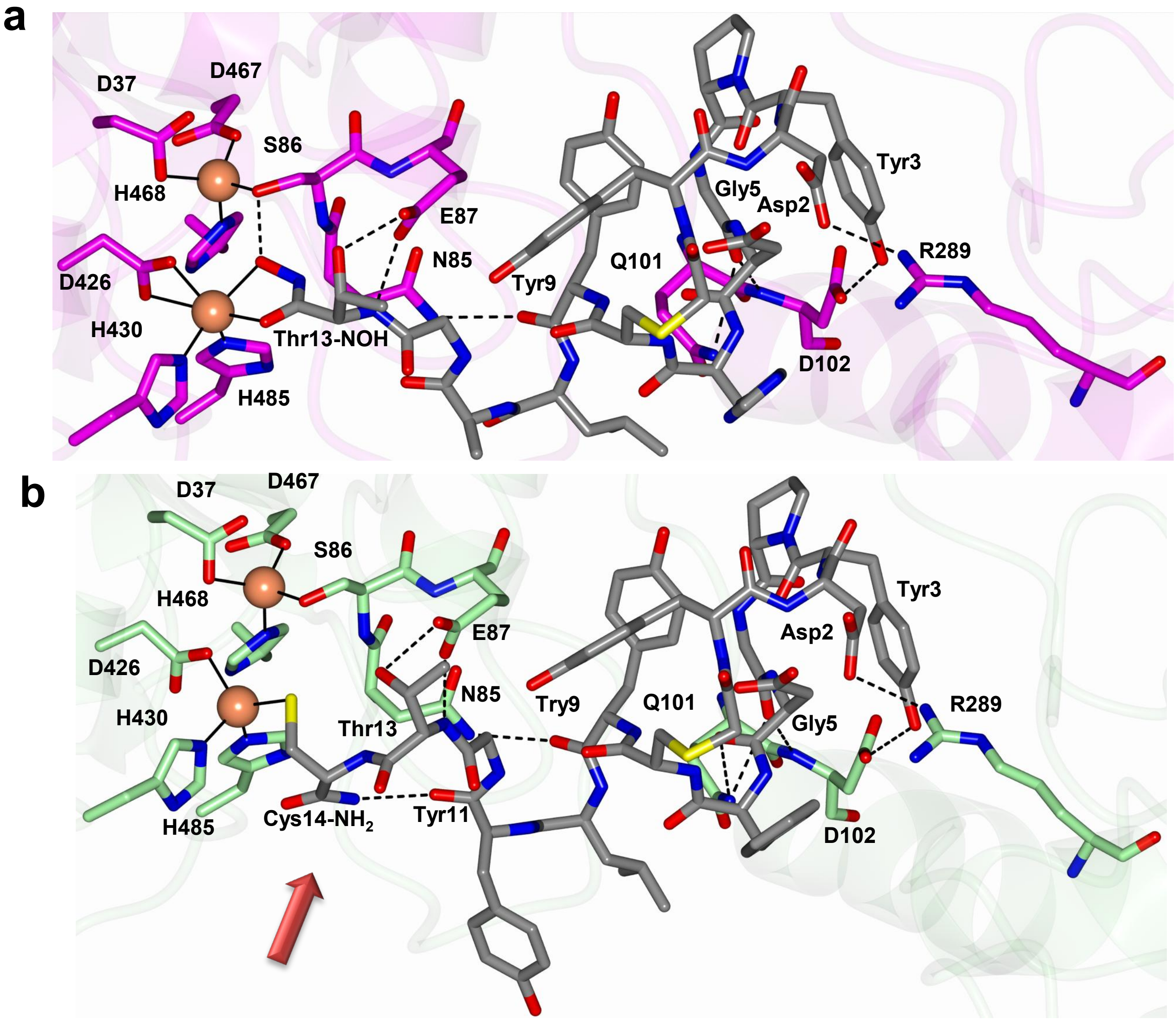

Supplementary Figure 3. Binding modes of Ce-1 NHOH and Ce-2 Y7F. (**a**) Interactions between Ce-1 NHOH (gray cylinders) and *C. elegans* iPGM-Ce (magenta). The T13-hydroxamate residue coordinates one of the Zn ions (orange) and is 3.38 Å from the second Zn ion. (**b**) Interactions between Ce-2 Y7F (gray cylinders) and *C. elegans* iPGM-Ce (green). The sulfur atom of the C14 residue coordinates one of the Zn ions (orange) and the NH_2 group forms an intramolecular hydrogen bond with Y11. Red arrow indicates space from which linker attached encoding RNA would be located. For clarity three-letter amino acid code is used for ipglycermide ligand and single letter code for protein amino acids.

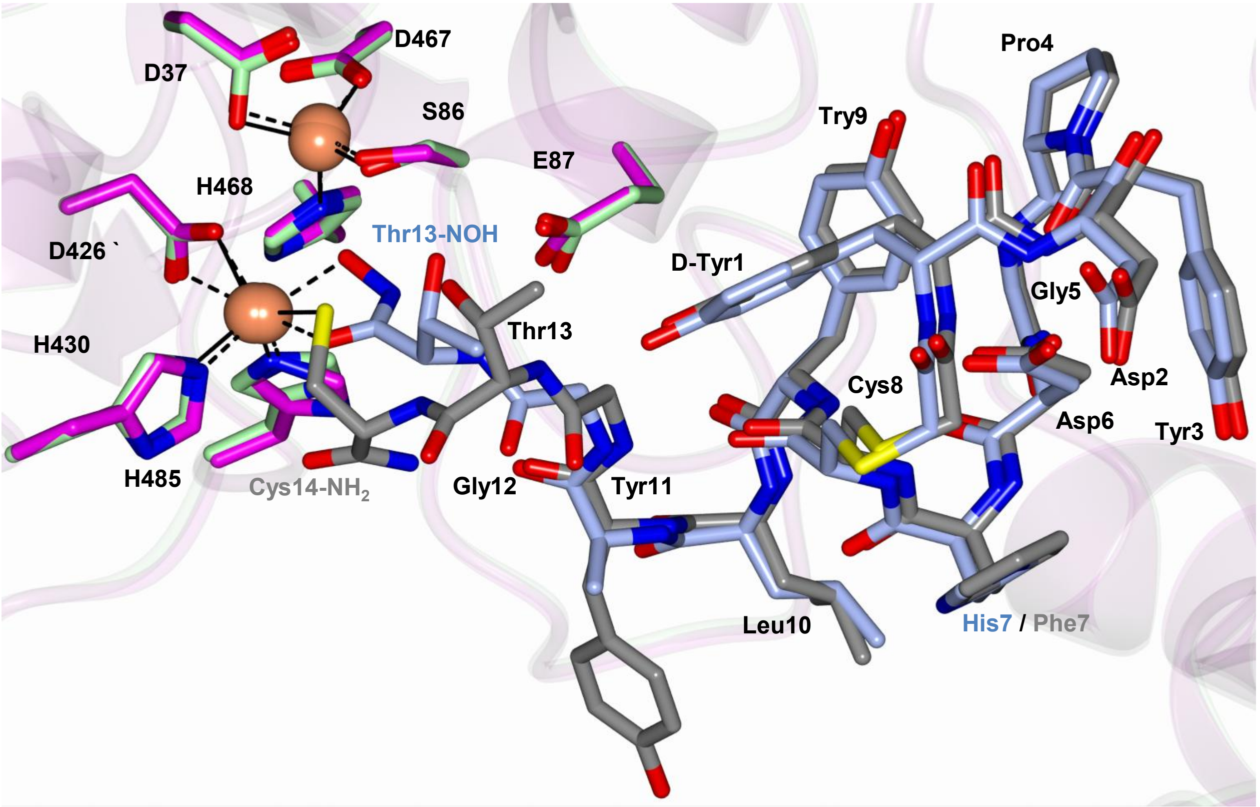

Supplementary Figure 4. Superposition of the *C. elegans* iPGM•Ce-1 NHOH (magenta/light blue) and *C. elegans* iPGM•Ce-2 Y7F (green/gray) structures. Coordination of *C. elegans* iPGM•Ce-1 NHOH and *C. elegans* iPGM•Ce-2 Y7F residues to the Zn ions (orange spheres) is indicated by the dotted and solid lines, respectively. For clarity three-letter amino acid code is used for ipglycermide ligand and single letter code for protein amino acids.

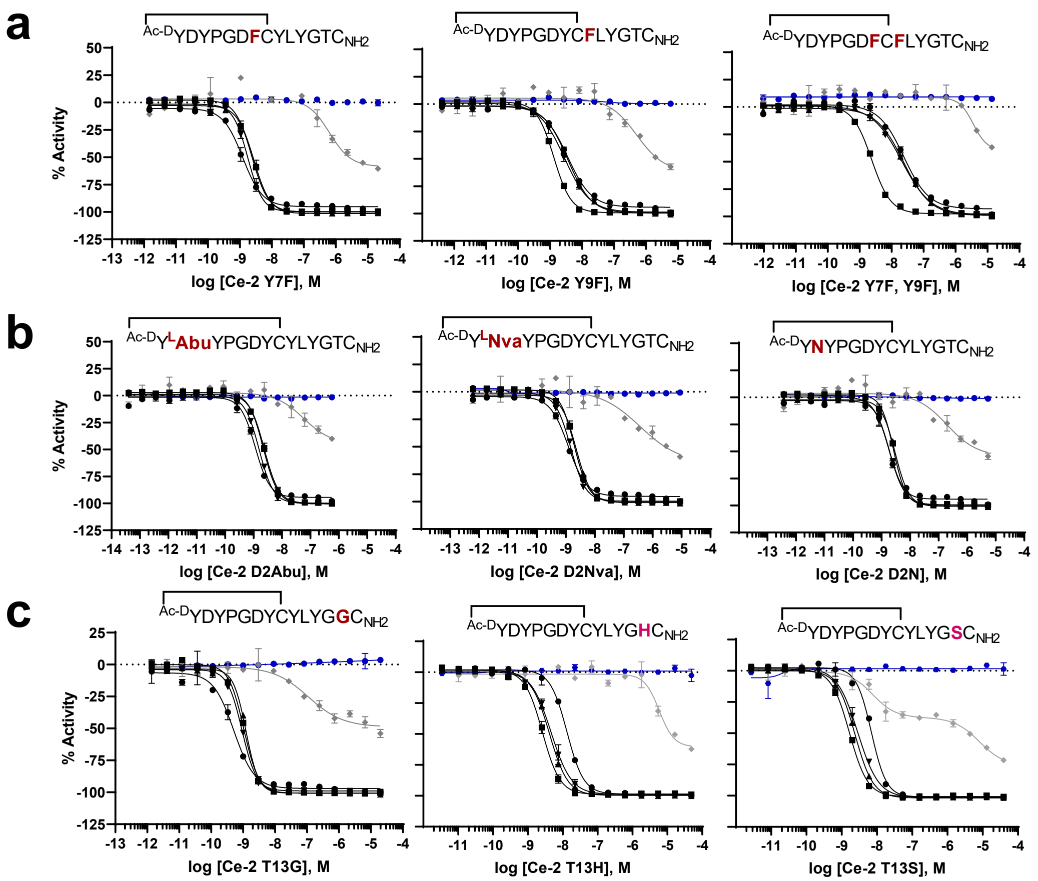

		Y7F	Y9F	Y7F,Y9F	D2Abu	D2Nva	D2N	T13G	T13H	T13S
Ce iPGM	% inhibtion	-99.6	-99.4	-99.3	-97.8	-99.7	-99.1	-100.7	-99.6	-100.4
	IC_{50} (M)	2.34E-09	1.31E-09	2.37E-09	1.15E-09	1.22E-09	1.86E-09	1.05E-09	2.07E-09	1.43E-09
Bm iPGM	% inhibtion	-99.5	-99.1	-98.2	-100.3	-99.8	-100	-97.1	-100.1	-100.7
	IC_{50} (M)	1.23E-09	3.53E-09	2.41E-08	2.20E-09	1.96E-09	2.64E-09	5.13E-10	8.63E-09	5.43E-09
Ov iPGM	% inhibtion	-101.1	-100.7	-99.9	-99.9	-101.6	-101.3	-100.6	-101.2	-101.2
	IC_{50} (M)	2.71E-09	3.57E-09	2.35E-08	2.38E-09	2.28E-09	3.03E-09	1.2E-09	3.61E-09	2.47E-09
Di iPGM	% inhibtion	-99.9	-99.4	-98.7	-100	-100.1	-100.1	-98.9	-100.2	-100.5
	IC_{50} (M)	1.78E-09	2.79E-09	2.03E-08	1.57E-09	1.46E-09	1.87E-09	9.2E-10	3.77E-09	2.56E-09
Ec iPGM	% inhibtion	-60.5	-57.2	-36.8	-40.1	-59.1	-54.5	-48.8	-66.2	-74.1
	IC_{50} (M)	6.38E-07	5.14E-07	3.58E-06	5.83E-08	3.69E-07	1.97E-07	1.10E-07	4.78E-06	9.93E-09
Hs dPGM	% inhibtion	-0.2	0.4	7.3	-1.5	-0.6	-1.3	4.4	-2.6	1.5
	IC_{50} (M)	NA	NA	NA	NA	NA	NA	NA	NA	NA

Supplemental Figure 5. Concentration response curves for (**a**) Ce-2 Y7, Y9 combination, (**b**) Ce-2 D2 and (**c**) Ce-2 T13 analogs

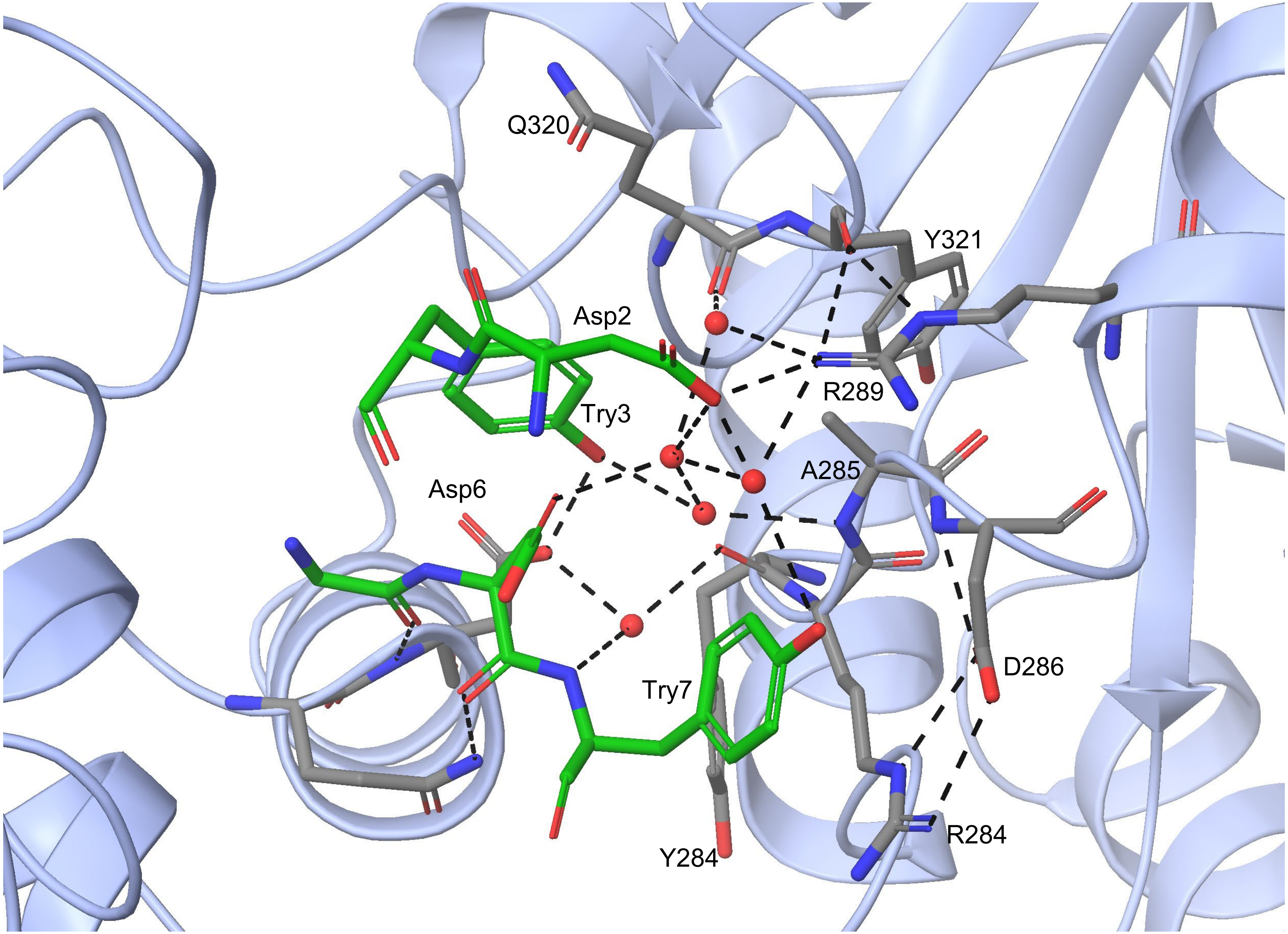

Supplementary Figure 6. Transferase-ipglycermide water network. Interaction of ipglycermide sidechains and mainchain atoms involved in water-mediated hydrogen bonding with iPGM transferase domain. Water molecules are red spheres, ipglycermide residues are in green, and iPGM residues are in grey. For clarity we use the three-letter amino acid code for ipglycermide ligand and single letter code for protein amino acids. Coordinates shown are from *C. elegans* iPGM•Ce-2d structure.

Supplementary Tables

- **Supplementary Table 1**. Intra-molecular ipglycermide hydrogen bonds
- **Supplementary Table 2**. X-ray data collection and refinement statistics
- **Supplementary Table 3**. Direct and water-mediated hydrogen bonds between ipglycermides and *C. elegans* iPGM
- **Supplementary Table 4a**. Ortholog iPGM panel pIC50 values for ipglycermide Ce-2 analogs
- **Supplementary Table 4b**. Ortholog iPGM panel pIC50 values for ipglycermide Ce-2d analogs
- **Supplementary Table 5**. SPR data for Ce-2 analogs
- **Supplementary Table 6**. Mutational Scanning conditions, HA purification efficiency and total library recovery
- **Supplementary Table 7.** HPLC and HRMS data for Ce-2 and Ce-2d analogs
- **Supplementary Table 8.** Primers used for the assembly of the NNK mutational library

Supplementary Table 1. Intra-molecular Ipglycermide hydrogen and aromatic bonds

Bond	Fig. ref.	Residue	Atom	Residue	Atom	Length (Å)		
						Ce-2d	Ce-1 NHOH	Ce-2 Y7F
a	a,b,c	1 (DTY)	N	6 (ASP)	OD2	2.98	3.02	2.99
b	a,b,c	1 (DTY)	N	6 (ASP)	OD1	3.03	3.10	2.8
c	a,b,c	2 (ASP)	N	6 (ASP)	OD1	2.72	2.92	2.86
d	a,b,c	3 (TYR)	N	6 (ASP)	OD1	3.28	3.47	3.41
e	a,b,c	3 (TYR)	N	3 (TYR)	O	2.71	2.71	2.74
f	a,b,c	6 (ASP)	N	3 (TYR)	O	3.14	3.12	3.03
g	a,b,c	8 (CYS)	N	6 (ASP)	OD2	3.23	3.26	3.11
h	a,b,c	9 (TYR)	N	6 (ASP)	O	3.07	2.98	3.05
i	a,b,c	10 (LEU)	N	7 (TYR)	O	3.02	3.08	3.27
j	a, b	11 (TYR)	N	8 (CYS)	O	3.05	3.54	NA
k	a	C-term amide	N	8 (CYS)	O	2.90	NA	NA
k	b, c	12 (GLY)	N	8 (CYS)	O	NA	3.02	2.99
l	c	C-term amide	N	11 (TYR)	O	NA	NA	2.76
x	a, b, c	1 (DTY)	Ar ring	9 (TYR)	Ar ring	4.91	5.03	4.89

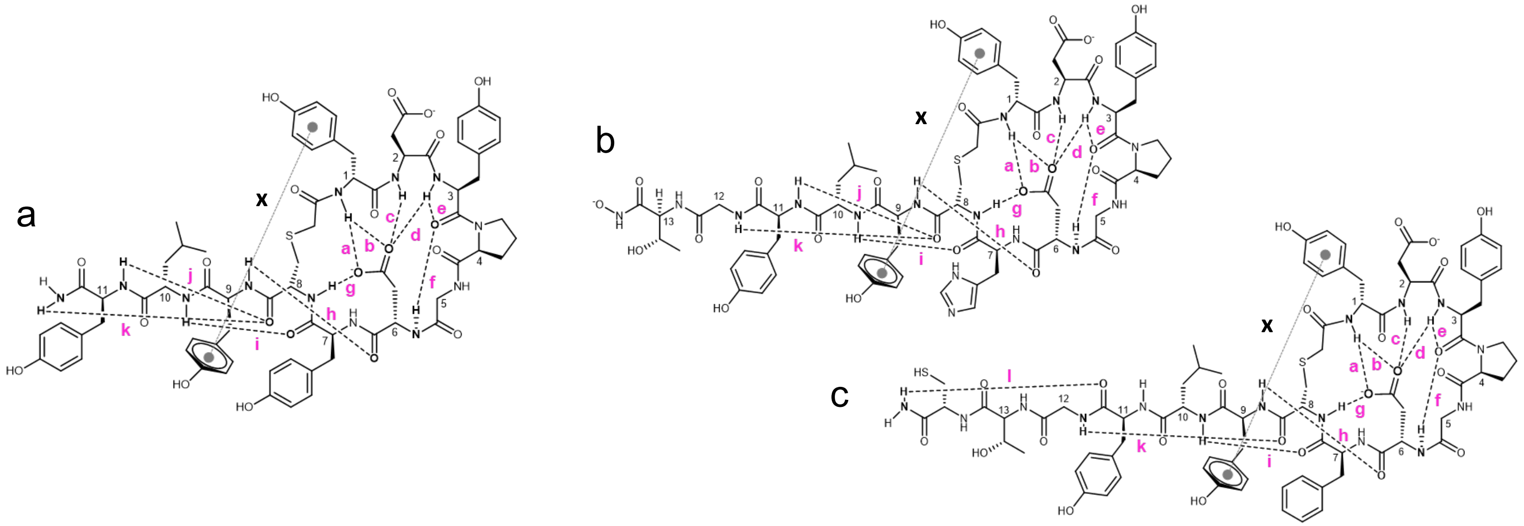

Supplementary Table 2. X-ray data collection and refinement statistics

	C. elegans iPGM•Ce-1 NHOH	*C. elegans* iPGM•Ce-2 Tyr7Phe
**Data Collection**		
Unit-cell parameters (Ă, °)	*a*=73.67, *b*=75.85, *c*=100.42, *β*=98.3	*a*=73.86, *b*=75.47, *c*=100.36, *β*=99.1
Space group	*P*2	*P*2
Resolution (Ă)[1]	48.60-1.80 (1.83-1.80)	44.69-2.10 (2.15-2.10)
Wavelength (Å)	1.0000	0.9198
Temperature (K)	100	100
Observed reflections	767,614	219,493
Unique reflections	101,405	64,166
$\langle I/\sigma(I)\rangle$[1]	11.2 (1.8)	8.9 (2.1)
Completeness (%)[1]	100 (99.8)	99.7 (99.9)
Multiplicity[1]	7.6 (7.5)	3.4 (3.4)
R_{merge} (%)[1, 2]	13.0 (121.7)	10.5 (58.1)
R_{meas} (%)[1, 4]	13.9 (130.6)	12.5 (68.8)
R_{pim} (%)[1, 4]	5.0 (47.1)	6.7 (36.5)
$CC_{1/2}$ [1, 5]	0.998 (0.735)	0.995 (0.783)
**Refinement**		
Resolution (Ă) [1]	44.13-1.80	44.69-2.10
Reflections (working/test)[1]	96,340/5,040	61,047/3,104
R_{factor} / R_{free} (%)[1,3]	15.2/19.4	16.7/22.0
No. of atoms (Protein/ Peptide/Water)	7,695/216/749	7807/242/399
**Model Quality**		
R.m.s deviations		
Bond lengths (Å)	0.009	0.011
Bond angles (°)	1.012	1.031
Mean *B*-factor ($Å^2$)		
All Atoms	24.1	30.9
Protein	23.4	30.7
Peptide	23.4	30.5
Water	32.2	34.2
Coordinate error (maximum likelihood) (Å)	0.18	0.23
Ramachandran Plot		
Most favored (%)	98.1	97.5
Additionally allowed (%)	1.6	2.3

Values in parenthesis are for the highest resolution shell. $R_{merge} = \Sigma_{hkl}\Sigma_i |I_i(hkl) - \langle I(hkl)\rangle| / \Sigma_{hkl}\Sigma_i I_i(hkl)$, where $I_i(hkl)$ is the intensity measured for the *i*th reflection and $\langle I(hkl)\rangle$ is the average intensity of all reflections with indices hkl. $R_{factor} = \Sigma_{hkl} ||F_{obs}(hkl)| - |F_{calc}(hkl)|| / \Sigma_{hkl} |F_{obs}(hkl)|$; Rfree is calculated in an identical manner using 5% of randomly selected reflections that were not included in the refinement. R_{meas} = redundancy-independent (multiplicity-weighted) R_{merge}[1,2]. R_{pim} = precision-indicating (multiplicity-weighted) R_{merge}[3,4]. $CC_{1/2}$ is the correlation coefficient of the mean intensities between two random half-sets of data [5,6]. 1. Evans, P.R., *An introduction to data reduction: space-group determination, scaling and intensity statistics.* Acta Crystallogr D Biol Crystallogr, 2011. 67(Pt 4): p. 282-92. 2. Evans, P., *Scaling and assessment of data quality.* Acta Crystallogr D Biol Crystallogr, 2006. 62(Pt 1): p. 72-82. 3. Diederichs, K. and P.A. Karplus, *Improved R-factors for diffraction data analysis in macromolecular crystallography.* Nat Struct Biol, 1997. 4(4): p. 269-75. 4. Weiss, M.S., *Global indicators of X-ray data quality.* Journal of Applied Crystallography, 2001. 34: p. 130-135. 5. Karplus, P.A. and K. Diederichs, *Linking crystallographic model and data quality.* Science, 2012. 336(6084): p. 1030-3. 6. Evans, P., *Biochemistry. Resolving some old problems in protein crystallography.* Science, 2012. 336(6084): p. 986-7.

Supplementary Table 3. Direct and water-mediated hydrogen bonds between ipglycermides and *C. elegans* iPGM

Ipglycermide residues		iPGM residue	H_2O mediated		Ce-2d	Ce-1 NHOH	Ce-2 Tyr7Phe
1(DTY)	O	G370	•	O	2.95, 2.85	3.12, 2.91	3.04, 2.74
2(ASP)	OD2	R289		NH2	2.62	2.79	2.85
	O	G370	•	O	2.82, 3.00	3.01, 3.10	NA
3(TYR)	OH	D102		OD2	2.55	2.52	2.7
	OH	A285	•	N	3.18, 2.92	3.29,2.91	2.66, 3.23
4(PRO)	O	A334	•	N	2.59, 2.91	2.59, 2.87	2.68, 2.84
5(GLY)	O	D102		N	2.93	2.9	2.99
6(ASP)	O	Q101		NE2	3.37	3.39	3.16
7(TYR)	N	D102	•	OD2	3.08, 2.60	3.19, 2.55	3.00, 2.54
	N	Y283	•	O	3.08, 2.80	3.19, 2.65	3.00, 2.94
	OH	R289	•	NH2	3.29, 2.88	NA	NA
(HIS)	ND1	A285	•	N	NA	3.35, 2.91	NA
9(TYR)	O	N85		ND2	2.89	2.94	2.85
	OH	G370	•	O	2.69, 2.85	2.65, 2.91	2.64, 2.74
10(LEU)	O	N85	•	ND2	2.84, 3.03	2.77, 2.96	2.75, 3.04
	O	M83	•	O	2.84, 2.73	2.77, 2.81	2.75, 2.97
11(TYR)	O	E87	•	OE1	3.13, 2.53	NA	NA
	O	H485	•	ND1	3.35, 3.18	2.88, 3.02	NA
	O	S86	•	N	3.13, 3.47	NA	NA
12(GLY)	N	E87		OE1	2.98	NA	NA
13(THR)	N	E87		OE1	NA	2.69	3.11
	N	E87		OE2	NA	NA	3.32
	OG1	E87		OE2	NA	2.58	2.81
	OG1	LYN359	•	NZ	NA	2.68, 2.72	2.94, 2.79
14(HA)	O	S86		N	NA	3.16	NA
	O	S86		OG	NA	2.28	NA
(CYS)	N	S86	•	N	NA	NA	3.11, 3.13
	N	E87	•	OE1	NA	NA	3.11, 2.78
	O	H485	•	N	NA	NA	2.80, 3.07

Notes: Atom type: OH, hydroxyl; O, carbonyl; C, carbon; N, nitrogen. Atom position: B, beta; G, gamma; D, delta; E, epsilon; Z, zeta; H, eta. Side 1: right side of the ring (example) Side 2: left side of the ring (example) LYN, neutral zeta amino group on lysine

Supplementary Table 4a. Ortholog iPGM panel IC_{50} values for ipglycermide Ce-2 analogs.

	Ce iPGM		Bm iPGM		Ov iPGM		Di iPGM		Ec iPGM	
**Ce-2 Analog**	Ave IC_{50} (M)	SEM	Ave IC_{50} (M)	SEM	Ave IC_{50} (M)	SEM	Ave IC_{50} (M)	SEM	Ave IC_{50} (M)	SEM
Ce-2	8.89E-10	2.18E-10	7.72E-10	4.65E-10	1.24E-09	2.63E-10	8.27E-10	1.65E-10	1.29E-07	6.64E-08
Ce-2d	2.58E-09	3.85E-10	3.96E-08	1.84E-08	8.80E-08	1.08E-08	7.52E-08	7.75E-09	NA	-
Ce-2 D2Abu	1.85E-09	4.38E-10	1.37E-09	7.23E-10	2.24E-09	6.25E-10	1.52E-09	3.79E-10	5.83E-08	NA
Ce-2 D2Nva	1.69E-09	4.26E-10	1.37E-09	6.10E-10	2.20E-09	6.28E-10	1.51E-09	4.07E-10	8.73E-06	1.44E-05
Ce-2 D2N	2.27E-09	5.37E-10	2.13E-09	7.39E-10	2.90E-09	6.92E-10	1.99E-09	5.74E-10	1.70E-07	3.85E-08
Ce-2 D2H	1.86E-09	4.11E-10	1.46E-09	6.49E-10	2.54E-09	7.50E-10	1.78E-09	4.56E-10	2.05E-06	3.20E-06
Ce-2 Y3F	1.60E-09	3.61E-10	3.69E-09	2.02E-10	3.22E-09	5.98E-10	2.42E-09	3.03E-10	>1E06	-
Ce-2 Y3(4F)F	1.56E-09	4.02E-10	1.40E-09	6.05E-10	2.10E-09	4.50E-10	1.52E-09	3.45E-10	4.18E-06	1.03E-06
Ce-2 P4V	2.17E-09	4.79E-10	1.89E-09	9.35E-10	2.96E-09	5.98E-10	2.14E-09	4.48E-10	8.96E-06	1.54E-05
Ce-2 Y7F	1.90E-09	4.38E-10	1.46E-09	8.42E-10	2.45E-09	6.09E-10	1.73E-09	3.67E-10	1.17E-06	9.24E-07
Ce-2 Y7(4F)F	1.79E-09	4.41E-10	1.42E-09	7.75E-10	2.28E-09	4.55E-10	1.67E-09	4.01E-10	1.28E-05	2.14E-05
Ce-2 Y9F	1.07E-09	2.51E-10	3.53E-09	1.93E-10	3.36E-09	3.88E-10	2.69E-09	2.53E-10	8.15E-07	2.83E-07
Ce-2 Y9(4F)F	1.79E-09	3.49E-10	3.31E-09	3.51E-10	3.84E-09	5.94E-10	2.80E-09	4.13E-10	6.93E-06	1.02E-05
Ce-2 [Y1-->C14]	2.00E-06	3.77E-07	1.59E-05	1.97E-05	5.20E-06	1.20E-06	6.52E-06	3.84E-06	NA	-
$^{Ac-}$YGTC$_{NH2}$	9.99E-04	-	NA	-	>1E-03	-	>1E-03	-	NA	-
Ce-2 Y3(4F)F, Y7F	9.03E-10	1.70E-10	2.12E-09	9.56E-11	1.87E-09	2.99E-10	1.41E-09	1.79E-10	>1E06	-
Ce-2 Y7F, Y9F	2.02E-09	3.89E-10	2.49E-08	6.63E-09	2.36E-08	1.16E-09	2.07E-08	8.81E-10	4.46E-06	8.36E-07
Ce-2 T13G	1.49E-09	6.30E-10	8.11E-10	4.22E-10	1.61E-09	5.42E-10	1.08E-09	2.24E-10	9.94E-08	1.51E-08
Ce-2 T13S	1.43E-09	3.46E-10	5.43E-09	2.20E-09	2.47E-09	1.27E-10	2.56E-09	4.45E-10	9.93E-09	3.36E-09
Ce-2 T13H	2.07E-09	7.78E-10	8.63E-09	6.46E-09	3.61E-09	7.64E-10	3.77E-09	1.20E-09	4.78E-06	1.55E-06
Ce-2 [12-13]GABA	1.49E-09	3.50E-10	3.12E-09	7.31E-10	4.02E-09	7.56E-10	2.85E-09	4.16E-10	2.98E-06	1.95E-06
Ce-2 C14NHOH	2.55E-09	5.20E-10	6.70E-09	3.87E-09	3.33E-09	8.41E-10	2.55E-09	6.31E-10	1.40E-05	2.04E-05
Ce-2 T13G, C14H	6.84E-09	3.88E-09	6.96E-08	2.90E-08	1.17E-07	1.49E-08	1.12E-07	9.99E-09	1.27E-05	3.71E-06
Ce-2 Y7H, C14NHOH	2.05E-09	4.35E-10	4.73E-09	3.41E-09	3.97E-09	8.12E-10	2.92E-09	5.78E-10	8.97E-06	1.12E-06
Ce-2 T13G, C14NHOH	4.96E-09	8.68E-10	1.29E-07	1.46E-08	2.02E-07	1.53E-08	1.84E-07	1.45E-08	NA	-

Notes: Abbreviations used in table are as follows: Abu, L-2-aminobutyric acid; Nva, L-norvaline; (4F)F, 4-fluorophenylalanine; GABA, γ-aminobutyric acid; [ ], amino acids replaced by indicated moiety. IC_{50} concentration are in molar. SEM calculated from the N=2-3 independent experiments.

Supplementary Table 4b. Ortholog iPGM panel IC_{50} values for ipglycermide Ce-2d analogs.

	Ce iPGM		Bm iPGM		Ov iPGM		Di iPGM		Ec iPGM	
**Ce-2d Analog**	Ave IC_{50} (M)	SEM	Ave IC_{50} (M)	SEM	Ave IC_{50} (M)	SEM	Ave IC_{50} (M)	SEM	Ave IC_{50} (M)	SEM
Ce-2	8.89E-10	2.18E-10	7.72E-10	4.65E-10	1.24E-09	2.63E-10	8.27E-10	1.65E-10	1.29E-07	6.64E-08
Ce-2d	2.58E-09	3.85E-10	3.96E-08	1.84E-08	8.80E-08	1.08E-08	7.52E-08	7.75E-09	NA	-
Ce-2d Y1(4F)F	2.49E-09	2.09E-09	3.39E-08	2.71E-08	1.26E-07	4.85E-08	5.35E-08	4.97E-08	NA	-
Ce-2d $Y1^{D}$(4F)F	2.90E-09	2.05E-09	2.37E-08	2.26E-08	6.39E-08	1.30E-08	3.70E-08	3.17E-08	NA	-
Ce-2d Y1(4OMe)F	4.48E-09	1.78E-09	1.58E-08	9.49E-10	3.87E-08	2.11E-09	1.87E-08	1.75E-08	2.00E-05	-
Ce-2d $Y1^{D}$(4OMe)F	1.35E-08	3.20E-09	3.95E-08	1.41E-08	1.53E-07	2.95E-08	6.18E-08	3.78E-08	1.87E-05	-
Ce-2d D2E	9.62E-09	3.14E-09	7.03E-08	7.12E-09	2.79E-07	5.26E-08	9.99E-08	6.58E-08	NA	-
Ce-2d D2Q	2.27E-08	5.56E-09	2.77E-07	8.13E-08	8.14E-07	1.40E-07	3.31E-07	1.79E-07	8.04E-06	-
Ce-2d D2G	5.66E-08	3.03E-08	9.90E-07	2.58E-07	3.86E-06	7.80E-07	1.32E-06	5.60E-07	NA	-
Ce-2d D2V	6.61E-08	1.48E-08	4.55E-07	9.86E-08	1.29E-06	2.72E-07	5.77E-07	3.31E-07	NA	-
Ce-2d Y3(4F)F	3.76E-08	3.93E-08	9.19E-08	8.32E-08	3.76E-07	1.26E-07	1.48E-07	9.68E-08	NA	-
Ce-2d Y3F	4.42E-07	1.09E-07	3.80E-06	3.91E-06	1.22E-05	9.64E-06	3.20E-06	6.82E-07	NA	-
Ce-2d Y3W	7.59E-07	5.24E-07	2.68E-06	6.15E-07	1.12E-05	1.32E-05	7.09E-06	6.21E-06	NA	-
Ce-2d P4V	4.01E-09	3.32E-09	2.41E-08	1.12E-08	1.21E-07	1.60E-08	5.01E-08	3.87E-08	2.67E-06	1.68E-07
Ce-2d P4I	6.05E-09	2.91E-09	1.19E-07	2.58E-08	5.21E-07	5.66E-08	2.21E-07	1.61E-07	2.19E-05	1.69E-05
Ce-2d P4A	6.76E-09	2.04E-09	3.66E-08	1.71E-08	1.85E-07	4.60E-09	7.94E-08	5.99E-08	NA	-
Ce-2d P4H	6.96E-08	7.66E-08	NA	-	NA	-	NA	-	NA	-
Ce-2d G5L	2.23E-08	3.18E-09	1.47E-06	3.72E-07	3.89E-06	1.78E-06	7.28E-05	1.23E-04	1.32E-05	-
Ce-2d G5Q	1.19E-07	3.30E-08	6.62E-06	5.39E-06	1.30E-05	3.55E-06	5.99E-06	1.65E-06	NA	-
Ce-2d D6N	3.81E-07	2.54E-07	3.72E-06	2.45E-06	9.41E-06	6.28E-06	6.18E-06	2.57E-06	NA	-
Ce-2d D6G	4.25E-06	2.52E-06	NA	-	NA	-	NA	-	NA	-
Ce-2d Y7W	3.15E-09	2.19E-09	4.09E-08	3.02E-08	1.05E-07	2.07E-08	6.10E-08	5.91E-08	NA	-
Ce-2d Y7(4OMe)F	5.69E-09	3.21E-09	5.15E-08	3.49E-09	1.61E-07	2.01E-08	6.79E-08	3.37E-08		-
Ce-2d Y7(4F)F	6.36E-09	5.33E-09	1.09E-07	8.21E-08	3.02E-07	7.30E-08	1.70E-07	1.45E-07	NA	-
Ce-2d Y7H	1.36E-08	5.89E-09	2.05E-07	1.36E-08	9.95E-07	2.10E-07	3.26E-07	2.63E-07	NA	-
Ce-2d Y7F	1.47E-08	5.79E-10	2.00E-07	7.56E-08	7.51E-07	1.43E-07	2.50E-07	1.96E-07		-
Ce-2d Y7S	1.62E-08	1.32E-09	9.50E-08	2.64E-08	4.21E-07	5.56E-08	1.78E-07	1.56E-07	NA	-
Ce-2d Y7A	5.47E-08	3.66E-08	2.78E-07	7.60E-08	9.10E-07	1.12E-07	3.98E-07	1.51E-07	NA	-
Ce-2d Y9(4OMe)F	3.79E-08	1.26E-08	1.20E-06	1.29E-07	3.27E-06	2.64E-06	2.04E-06	1.81E-06	NA	-
Ce-2d Y9(4F)F	3.80E-08	3.70E-08	2.33E-07	1.88E-07	7.64E-07	2.20E-07	3.33E-07	2.98E-07	NA	-
Ce-2d Y9F	1.25E-07	6.83E-08	1.52E-06	3.86E-07	4.19E-05	3.94E-05	1.43E-05	9.78E-06	NA	-
Ce-2d L10I	2.67E-08	1.10E-08	3.74E-07	9.91E-08	1.32E-06	4.62E-07	5.03E-07	4.14E-07	NA	-
Ce-2d L10V	1.37E-07	1.88E-08	1.31E-06	2.19E-07	6.66E-06	5.57E-06	1.93E-06	1.28E-06	NA	-
Ce-2d Y11(4F)F	3.69E-09	1.98E-09	4.743E-08	2.8E-08	1.40E-07	3.09E-08	7.11E-08	5.89E-08	9.29E-05	-
Ce-2d Y11(4OMe)F	6.31E-09	2.33E-09	6.972E-08	1.98E-08	1.55E-07	4.45E-08	6.05E-08	3.18E-08	1.28E-05	-
Ce-2d Y11F	8.00E-09	3.93E-09	5.5303E-08	1.66E-08	2.14E-07	1.03E-08	9.89E-08	9.52E-08	NA	-
Ce-2d Y11L	1.23E-08	7.15E-10	1.8917E-07	6.53E-08	7.21E-07	1.38E-07	2.43E-07	7.51E-08	NA	-
Ce-2d Y11COOH	4.64E-08	3.15E-08	5.12E-07	1.26E-07	6.98E-07	4.38E-08	7.75E-07	3.49E-07	3.65E-05	-

Supplementary Table 5. SPR data for ipglycermide analogs

ipglycermide	iPGM	k_{on} ($M^{-1}s^{-1}$)	SEM	%error	k_{off} (s^{-1})	SEM	%error	K_D	SEM	%error	$t_{1/2}$	SEM	%error
Ce-2	*C.e.*	1.11E+06	5.36E+05	48.1	3.23E-05	9.02E-06	28.0	3.82E-11	2.29E-11	60.1	6.7 h	1.7	26.0
Ce-2d	*C.e.*	2.08E+06	1.59E+06	76.7	2.13E-03	5.36E-04	25.2	2.39E-09	7.38E-10	30.8	6 min	2.7	45.2
Ce-1 NHOH	*C.e.*	1.86E+06	4.87E+05	26.2	1.71E-04	8.10E-05	47.3	1.09E-10	6.89E-11	63.4	1.5 h	0.7	47.6
Ce-2 T13G NHOH	*C.e.*	1.97E+05	3.25E+04	16.5	2.51E-03	2.35E-04	9.4	1.63E-08	2.50E-10	1.5	4.3 min	0.4	9.5
Ce-2 P4V	*C.e.*	1.73E+05	3.90E+04	22.5	1.15E-05	2.47E-06	21.4	6.68E-11	9.00E-13	1.3	17.5 h	3.7	21.4
Ce-2 D2Abu	*C.e.*	3.04E+06	7.45E+05	24.5	4.55E-05	3.45E-06	7.6	1.57E-11	2.75E-12	17.6	4.3 h	0.3	7.6
Ce-2 D2Nva	*C.e.*	1.94E+06	3.15E+05	16.3	4.98E-05	9.15E-06	18.4	2.57E-11	5.50E-13	2.1	4.0 h	0.7	18.4
Ce-2 T13G	*C.e.*	1.32E+06	5.90E+05	44.6	5.77E-05	2.77E-05	48.1	4.33E-11	4.02E-12	9.3	4.3 h	1.9	45.5
Ce-2 T13S	*C.e.*	2.86E+06	6.60E+05	23.1	7.83E-05	2.05E-06	2.6	2.87E-11	5.90E-12	20.6	2.5 h	0.1	2.6
Ce-2 T13H	*C.e.*	7.52E+05	3.45E+04	4.6	1.98E-05	7.00E-07	3.5	2.64E-11	3.00E-13	1.1	9.7 h	0.3	3.4
Ce-2	*B.m.*	4.82E+05	6.02E+04	12.5	2.24E-04	1.01E-05	4.5	4.70E-10	4.89E-11	10.4	52 min	2.4	4.6
Ce-2d	*B.m.*	5.31E+05	4.82E+05	90.7	1.90E-02	6.30E-03	33.2	5.81E-08	1.89E-08	32.5	70 s	14	34.6
Ce-1 NHOH	*B.m.*	2.03E+05	8.79E+04	43.2	1.48E-03	1.48E-04	10.0	1.15E-08	1.71E-09	14.9	7.9 min	0.9	10.7
Ce-2 T13G NHOH	*B.m.*	1.10E+05	3.50E+03	3.2	2.84E-02	4.00E-04	1.4	2.45E-07	5.00E-10	0.2	23 s	0.4	1.6
Ce-2 P4V	*B.m.*	1.94E+05	3.69E+04	19.1	7.52E-05	6.41E-06	8.5	4.07E-10	1.02E-10	2.6	21.2 h	25.7	8.1
Ce-2 D2Abu	*B.m.*	5.61E+05	1.50E+03	0.3	1.25E-04	6.00E-06	4.8	2.23E-10	1.20E-11	5.4	1.5 h	0.1	5.0
Ce-2 D2Nva	*B.m.*	3.76E+05	1.50E+04	4.0	1.43E-04	7.50E-06	5.3	3.78E-10	5.00E-12	1.3	1.4 h	0.1	5.3
Ce-2 T13G	*B.m.*	1.19E+06	2.04E+05	17.1	6.11E-05	2.66E-05	43.5	4.93E-11	1.59E-11	32.3	4.1 h	2.2	53.8
Ce-2 T13S	*B.m.*	1.47E+06	7.84E+05	53.3	7.84E-05	2.19E-05	28.0	8.53E-11	6.00E-11	70.4	2.7 h	0.7	28.0
Ce-2 T13H	*B.m.*	1.06E+06	2.00E+04	1.9	5.87E-04	2.00E-05	3.4	5.55E-10	2.95E-11	5.3	20 min	0.7	3.4
Ce-2	*E.c.*	1.02E+05	3.07E+04	30.0	4.84E-03	4.55E-04	9.4	6.92E-08	2.40E-08	34.6	2.4 min	0.3	10.3
Ce-2d	*E.c.*	>LOQ	na	na	>LOQ	na	na	1.37E-05	5.25E-06	38.3	<1 s	na	na
Ce-1 NHOH	*E.c.*	1.81E+05	1.95E+05	107.9	9.14E-01	8.42E-01	92.2	5.14E-06	2.17E-06	42.3	1.1 s	0.4	39.2
Ce-2 T13G NHOH	*E.c.*	>LOQ	na	na	>LOQ	na	na	ND	na	na	<1 s	na	na
Ce-2 P4V	*E.c.*	3.73E+04	1.00E+02	0.3	2.72E-04	1.25E-05	4.6	7.29E-09	3.05E-10	4.2	42.6 min	2.0	4.5
Ce-2 D2Abu	*E.c.*	2.70E+05	8.45E+04	31.4	2.02E-03	4.65E-04	23.1	2.54E-08	8.50E-10	3.4	6.1 min	1.4	23.2
Ce-2 D2Nva	*E.c.*	1.32E+05	3.00E+03	2.3	1.95E-03	1.00E-04	5.1	4.71E-08	4.60E-09	9.8	6.0 min	0.3	5.2
Ce-2 T13G	*E.c.*	2.99E+05	7.50E+03	2.5	1.33E-03	2.00E-05	1.5	1.08E-08	5.00E-11	0.5	8.7 min	0.1	1.6
Ce-2 T13S	*E.c.*	2.36E+05	7.40E+04	31.3	3.33E-04	1.91E-04	57.4	1.31E-09	5.87E-10	44.7	44 min	18.8	42.5
Ce-2 T13H	*E.c.*	1.03E+05	3.09E+04	30.0	1.44E-02	1.57E-03	10.9	6.27E-08	2.11E-08	33.7	49 s	0.1	11.6

Notes: LoQ, limit of quantitation; na, not applicable; ND, not determined

Supplementary Table 6. Mutational Scanning conditions, HA purification efficiency and total library recovery

bead washing conditions	washing volume	protein conc. during washes	fold recovery (target. vs no target. selection)	HA purification recovery %	recovery % (output vs input)
6h, 12h, 6h	200 µL	50 nM	186	10%	15%

Supplementary Table 7a. HPLC and HRMS data for Ce-2 analogs

Sample ID	Sequence	Analysis method	HPLC t_r (min)	Purity (%)	Calc'd exact mass	Obs ion	Obs ion mass
Ce-2	(Ac)*Y* DYPGDYCYLYGTC-NH2	1	5.76	>97	1733.64	$[M+Na]^+$	1756.12
Ce-2d	(Ac)*Y* DYPGDYCYLY-NH2	2a	7.81	>95	1473.56	[M-H]-	1472.50
Ce-2 D2Abu	(Ac)*Y* AbuYPGDYCYLYGTC-NH2	1	6.38	>80	1726.65	$[M+Na]^+$	1726.95
Ce-2 D2Nva	(Ac)*Y* NvaYPGDYCYLYGTC-NH2	1	6.78	>89	1740.67	$[M+Na]^+$	1741.22
Ce-2 D2N	(Ac)*Y* NYPGDYCYLYGTC-NH2	2a	7.57	>73	1732.65	$[M+H]^+$	1733.66
Ce-2 Y3F	(Ac)*Y* DFPGDYCYLYGTC-NH2	1	6.66	>89	1717.64	$[M+Na]^+$	1740.23
Ce-2 Y3(4F)	(Ac)*Y* D(4F)FPGDYCYLYGTC-NH2	1	6.92	>87	1735.63	$[M+Na]^+$	1758.62
Ce-2 P4V	(Ac)*Y* DYVGDYCYLYGTC-NH2	2a	9.36	>80	1735.65	$[M+H]^+$	1736.67
Ce-2 Y7F	(Ac)*Y* DYPGDFCYLYGTC-NH2	1	6.92	>92	1717.64	$[M+Na]^+$	1740.33
Ce-2 Y7(4F)	(Ac)*Y* DYPGD(4F)FCYLYGTC-NH2	1	7.09	>92	1735.63	$[M+Na]^+$	1758.45
Ce-2 Y9F	(Ac)*Y* DYPGDYCFLYGTC-NH2	1	6.72	>94	1717.64	$[M+Na]^+$	1740.63
Ce-2 Y9(4F)	(Ac)*Y* DYPGDYC(4F)FLYGTC-NH2	1	6.89	>93	1735.63	$[M+Na]^+$	1758.69
Ce-2 [Y1-->C14]	(Ac)*Y* DYPGDYCYLYGTC-NH2	1	6.04	>90	1733.64	$[M+Na]^+$	1756.15
$^{Ac-}YGTC_{NH2}$	(Ac)YGTC-NH2	2b	9.47	>86	483.54	$[M+H]^+$	484.20
Ce-2 Y3(4F), Y7F	(Ac)*Y* D(4F)FPGDFCYLYGTC-NH2	1	8.00	>93	1719.64	$[M+Na]^+$	1742.40
Ce-2 Y7F, Y9F	(Ac)*Y* DYPGDFCFLYGTC-NH2	1	7.78	>92	1701.65	$[M+Na]^+$	1724.72
Ce-2 T13S	(Ac)*Y* DYPGDYCYLYGSC-NH2	2a	7.72	>90	1720.85	$[M+H]^+$	1721.40
Ce-2 T13H	(Ac)*Y* DYPGDYCYLYGHC-NH2	2a	7.51	>85	1770.91	$[M+H]^+$	1771.66
Ce-2 T13G	(Ac)*Y* DYPGDYCYLYGGC-NH2	1	5.86	>91	1689.61	$[M+Na]^+$	1712.22
Ce-2 [12-13]GABA	(Ac)*Y* DYPGDYCYLY[GABA]C-NH2	2a	8.34	>90	1660.62	$[M+H]^+$	1661.62
Ce-2 C14NHOH	(Ac)*Y* DYPGDYCYLYGT-NHOH	1	5.28	>98	1646.62	[M-H]-	1645.58
Ce-2 T13G, C14H	(Ac)*Y* DYPGDYCYLYGGH-NH2	2a	6.60	>94	1724.82	$[M+H]^+$	1725.50
Ce-2 Y7H, C14NHOH	(Ac)*Y* DYPGDHCYLYGT-NHOH	1	4.24	>91	1620.62	$[M+Na]^+$	1643.19
Ce-2 T13G, C14NHOH	(Ac)*Y* DYPGDYCYLYGG-NHOH	2a	6.84	>90	1603.68	$[M+H]^+$	1604.61

Notes: Underline indicates points of cyclization. Blue residues indicates substitutions relative to Ce-2 or Ce-2d. Italics indicated D-amino acid. Analysis method are detailed in Supplemental Methods.

Supplementary Table 7b. HPLC and HRMS data for Ce-2d analogs

Sample ID	Sequence	Analysis method	HPLC t_r (min)	Purity (%)	Calc'd exact mass	Obs ion	Obs ion mass
Ce-2d Y1(4F)F	(Ac)-(4F)FDYPGDYCYLY-NH2	2a	11.15	>96	1474.56	[M+H]$^+$	1475.57
Ce-2d Y1^{D}(4F)F	(Ac)-*(4F)F* DYPGDYCYLY-NH2	2a	11.30	>94	1474.56	[M+H]$^+$	1475.57
Ce-2d Y1(4OMe)F	(Ac)(4OMe)FDYPGDYCYLY-NH2	2a	10.70	>91	1486.58	[M+H]$^+$	1487.59
Ce-2d Y1^{D}(4OMe)F	(Ac)*(4OMe)F* DYPGDYCYLY-NH2	2a	10.54	>92	1486.58	[M+H]$^+$	1487.59
Ce-2d D2E	(Ac)*Y* EYPGDYCYLY-NH2	2a	7.93	>95	1486.58	[M+H]$^+$	1487.58
Ce-2d D2Q	(Ac)*Y* QYPGDYCYLY-NH2	2a	7.33	>79	1485.59	[M+H]$^+$	1486.60
Ce-2d D2G	(Ac)*Y* GYPGDYCYLY-NH2	2a	8.28	>92	1414.55	[M+H]$^+$	1415.56
Ce-2d D2V	(Ac)*Y* VYPGDYCYLY-NH2	2a	11.12	>86	1456.60	[M+H]$^+$	1457.61
Ce-2d Y3(4F)F	(Ac)*Y* D(4F)FPGDYCYLY-NH2	2a	11.44	>99	1474.56	[M+H]$^+$	1475.57
Ce-2d Y3F	(Ac)*Y* DFPGDYCYLY-NH2	2a	10.63	>94	1456.56	[M+H]$^+$	1457.58
Ce-2d Y3W	(Ac)*Y* DWPGDYCYLY-NH2	2a	11.19	>89	1495.58	[M+H]$^+$	1496.59
Ce-2d P4V	(Ac)*Y* DYVGDYCYLY-NH2	2a	9.12	>88	1474.58	[M+H]$^+$	1475.59
Ce-2d P4I	(Ac)*Y* DYIGDYCYLY-NH2	2a	10.60	>86	1488.59	[M+H]$^+$	1489.60
Ce-2d P4A	(Ac)*Y* DYAGDYCYLY-NH2	2a	7.60	>85	1446.54	[M+H]$^+$	1447.55
Ce-2d P4H	(Ac)*Y* DYHGDYCYLY-NH2	2a	6.78	>95	1512.57	[M+H]$^+$	1513.57
Ce-2d G5L	(Ac)*Y* DYPLDYCYLY-NH2	2a	13.17	>93	1528.62	[M+H]$^+$	1529.63
Ce-2d G5Q	(Ac)*Y* DYPQDYCYLY-NH2	2a	7.43	>83	1543.60	[M+H]$^+$	1544.61
Ce-2d D6N	(Ac)*Y* DYPGNYCYLY-NH2	2a	7.29	>89	1471.58	[M+H]$^+$	1472.59
Ce-2d D6G	(Ac)*Y* DYPGGYCYLY-NH2	2a	7.88	>88	1414.55	[M+H]$^+$	1415.56
Ce-2d Y7W	(Ac)*Y* DYPGDWCYLY-NH2	2a	11.62	>91	1495.58	[M+H]$^+$	1496.59
Ce-2d Y7(4OMe)F	(Ac)*Y* DYPGD(4OMe)FCYLY-NH2	2a	11.10	>93	1486.58	[M+H]$^+$	1487.59
Ce-2d Y7(4F)F	(Ac)*Y* DYPGD(4F)FCYLY-NH2	2a	11.95	>88	1474.56	[M+H]$^+$	1475.57
Ce-2d Y7H	(Ac)*Y* DYPGDHCYLY-NH2	2a	6.01	>95	1446.56	[M+H]$^+$	1447.57
Ce-2d Y7F	(Ac)*Y* DYPGDFCYLY-NH2	2a	11.12	>89	1456.56	[M+H]$^+$	1457.57
Ce-2d Y7S	(Ac)*Y* DYPGDSCYLY-NH2	2a	6.20	>80	1396.53	[M+H]$^+$	1397.54
Ce-2d Y7A	(Ac)*Y* DYPGDACYLY-NH2	2a	6.88	>91	1381.54	[M+H]$^+$	1382.55
Ce-2d Y9(4OMe)F	(Ac)*Y* DYPGDYC(4OMe)FLY-NH2	2a	10.90	>93	1486.58	[M+H]$^+$	1487.59
Ce-2d Y9(4F)F	(Ac)*Y* DYPGDYC(4F)FLY-NH2	2a	11.53	>99	1474.56	[M+H]$^+$	1475.56
Ce-2d Y9F	(Ac)*Y* DYPGDYCFLY-NH2	2a	10.77	>90	1456.56	[M+H]$^+$	1457.58
Ce-2d L10I	(Ac)*Y* DYPGDYCYIY-NH2	2a	7.44	>92	1472.56	[M+H]$^+$	1473.57
Ce-2d L10V	(Ac)*Y* DYPGDYCYVY-NH2	2a	6.54	>89	1458.54	[M+H]$^+$	1459.56
Ce-2d Y11(4F)F	(Ac)*Y* DYPGDYCYL(4F)F-NH2	2a	12.87	>86	1474.56	[M+H]$^+$	1475.57
Ce-2d Y11(4OMe)F	(Ac)*Y* DYPGDYCYL(4OMe)F-NH2	2a	11.70	>93	1486.58	[M+H]$^+$	1487.59
Ce-2d Y11F	(Ac)*Y* DYPGDYCYLF-NH2	2a	12.15	>90	1456.56	[M+H]$^+$	1457.58
Ce-2d Y11L	(Ac)*Y* DYPGDYCYLL-NH2	2a	10.80	>86	1422.58	[M+H]$^+$	1423.59
Ce-2d COOH	(Ac)*Y* DYPGDYCYLY-COOH	2a	8.87	>99	1473.54	[M+H]$^+$	1474.56

Notes: Underline indicates points of cyclization. Blue residues indicates substitutions relative to Ce-2 or Ce-2d. Italics indicated D-amino acid. Analysis method are detailed in Supplemental Methods.

Supplementary Table 8. Primers used for the assembly of the NNK mutational library

Primer	Sequence
(Prim_ext_1st_fw_NNK)_Oligo1	CTATAGGGTTAACTTTAAGAAGGAGATATACATATGNNKTATCCTGGTGATTATTGTTATCTGTAT
(Prim_ext_1st_fw_NNK)_Oligo2	CTATAGGGTTAACTTTAAGAAGGAGATATACATATGGATNNKCCTGGTGATTATTGTTATCTGTAT
(Prim_ext_1st_fw_NNK)_Oligo3	CTATAGGGTTAACTTTAAGAAGGAGATATACATATGGATTATNNKGGTGATTATTGTTATCTGTAT
(Prim_ext_1st_fw_NNK)_Oligo4	CTATAGGGTTAACTTTAAGAAGGAGATATACATATGGATTATCCTNNKGATTATTGTTATCTGTAT
(Prim_ext_1st_rv)_Oligo 5	TATGGGTAGCTGCCGCTACCACAAGTCCCATACAGATAACAATAATC
(Prim_ext_2nd_fw)_Oligo 6	CTATAGGGTTAACTTTAAGAAGGAGATATACATATGGATTATCCTGGT
(Prim_ext_2nd_rv_NNK)_Oligo 7	TATGGGTAGCTGCCGCTACCACAAGTCCCATACAGATAACAATAMNNACCAGGATAATCCATAT
(Prim_ext_2nd_rv_NNK)_Oligo 8	TATGGGTAGCTGCCGCTACCACAAGTCCCATACAGATAACAMNNATCACCAGGATAATCCATAT
(Prim_ext_2nd_rv_NNK)_Oligo 9	TATGGGTAGCTGCCGCTACCACAAGTCCCATACAGATAMNNATAATCACCAGGATAATCCATAT
(Prim_ext_2nd_rv_NNK)_Oligo 10	TATGGGTAGCTGCCGCTACCACAAGTCCCATACAGMNNACAATAATCACCAGGATAATCCATAT
(Prim_ext_2nd_rv_NNK)_Oligo 11	TATGGGTAGCTGCCGCTACCACAAGTCCCATAMNNATAACAATAATCACCAGGATAATCCATAT
(Prim_ext_2nd_rv_NNK)_Oligo 12	TATGGGTAGCTGCCGCTACCACAAGTCCCMNNCAGATAACAATAATCACCAGGATAATCCATAT
(Prim_ext_2nd_rv_NNK)_Oligo 13	TATGGGTAGCTGCCGCTACCACAAGTMNNATACAGATAACAATAATCACCAGGATAATCCATAT
(Prim_ext_2nd_rv_NNK)_Oligo 14	TATGGGTAGCTGCCGCTACCACAMNNCCCATACAGATAACAATAATCACCAGGATAATCCATAT
(Prim_ext_2nd_rv_NNK)_Oligo 15	TATGGGTAGCTGCCGCTACCMNNAGTCCCATACAGATAACAATAATCACCAGGATAATCCATAT
(Prim_ext_2nd_rv_NNK)_Oligo 16	TATGGGTAGCTGCCGCTMNNACAAGTCCCATACAGATAACAATAATCACCAGGATAATCCATAT
(PCR1_fw_F48_primer)_Oligo 17	TAATACGACTCACTATAGGGTTAACTTTAAGAAGGAGATATACATATG
(PCR1_rv)_Oligo 18	TTTCCGCCCCCCGTCCTAAGAACCAGAACCAGAACCTGCATAGTCGGGCACGTCGTATGGGTAGCTGCCGCT
(PCR2_fw_F48_primer)_Oligo 19	TAATACGACTCACTATAGGGTTAACTTTAAGAAGGAGATATACATATG
(PCR2_rv)_Oligo 20	TTTCCGCCCCCCGTCCTAAGAACCAGAACCAGAACC

Supplementary Protocols

- **Supplemental Protocol 1.** Phosphoglycerate mutase (PGM) protein expression and purification
- **Supplemental Protocol 2**. Phosphoglycerate mutase ortholog functional assay panel
- **Supplemental Protocol 3**. *In vitro* biotinylation
- **Supplemental Protocol 4**. Biacore S200 general experimental

Supplemental Protocol 1. Phosphoglycerate mutase (PGM) protein expression and purification

**Phosphoglycerate mutase (PGM) Protein Expression and Purification**			
**Sequence**	**Parameter**	**Value**	**Description**
1	Transformation	5-100 ng	1 µl of plasmid for transformation of BL21DE3 cells
2	Expression	1-2 days	Inoculation of growth media then induction with IPTG
3	Harvest	10 min	Centrifugation at 7000 RPM for 10 min. at 4 °C
4	Lysis	6 cycles	45s on/60s off 6 cycles of Sonication on ice
5	Chromatography	Affinity	Purification using 5 ml Nickel column to trap the ploy His tag
6	Purity	SDS-PAGE	Assess purity using 4-15% SDS-PAGE
7	Chromatography	Size exclusion	Further purification using gel filtration column to remove aggregation
8	Purity and concentration	SDS-PAGE	Assess purity using 4-15% SDS-PAGE, quantification using nano drop
9	Storage	-80 ^{o}C	Aliquot to 10/20 µl, flash freezing in LN2 for long term storage
10	Activity assay	ViewLux	See **Supplemental Protocol 2**

**Step**	**Notes**
1	cDNA was synthsized by Biobasics with the desired 5'- 3' restriction sites, ligated into the pET21a expression vector. Construct integrity confirmed by sequencing. Add 1 µl (5-100 ng) plasmid to the half tube of BL21DE3 cells (thawed on ice). Let sit on ice for 30 min. Heat shock the cells at 42 ^{o}C for 45 s, then immediately place on ice for 2 min. Add 250 µl of pre-warmed SOC medium. Shake for 1 h at 37 °C. Plate and spread 100 -150 µl on to pre-warmed LB/AMP 50 plate. Incubate the plate for overnight at 37 °C.
2	Inocuate 50 ml of autoclaved LB/AMP50 µg/ml media with a streak of freshly transfomred BL21DE3 cells, shake overnight at 37 °C (200 RPM). Inoculate 4 x 1 L LB/AMP50 flasks with 10 ml overnight starter cultures, grow at 37 °C to OD_{600} 0.5. Reduce the temperature to 16 ˚C, induce the cells with 0.4 mM IPTG (save 1 ml pre-induced sample for pre-expression control). Induce the cells overnight at 16 °C (200 RPM)
3	Remove 1 ml for confirmation of expression. Harvest 4 x 1 L cells at 7000 RPM (using pre-chilled 6 L rotor at 4°C). Gently scrape the pellet from centrifuge bottles using a spatula and transfer to zip lock bag. Spread paste evenly in the bag using hand, weigh, store @-80 °C until need for purification.
4	Add broken frozen pellet to a 100 ml glass beaker, add 30-40 ml of lysis buffer (20 mM sodium phosphate pH 7.4, 300 mM NaCl, 10 mM Imidazole-Buffer A). Resuspend the pellet in lysis buffer by stirring. Add 1 tablet (crushed) Roche protease inhibitor (EDTA free) to sample while resuspending. Lyse by sonication (6 cycles of 45s on/60 s off at 50 % capacity) on ice. Centrifuge at 20000 RPM for 25 min. to separate the cell debris from soluble protein (at 4 ˚C). Transfer supernatant in to a 50 ml tube keep on ice until purification.

Supplemental Protocol 1. continued

Step	Notes
5	30 to 50 ml of soluble lysate applied to equilibrated 5 ml His-Trap column at a flow rate of 1.5 ml/min (column equilibrate with 25 ml Buffer A). Wash unbound proteins from column with 30 ml Buffer A, and weakly bound proteins off with Buffer A with 50 mM Imidazole (8% Buffer B- Buffer A + 500 mM Imidazole). Elute His-tagged protein with a 25 ml gradient increasing Imidazole (50 to 500 mM- 8 to 100 % Buffer B), collect in 1 ml fractions. Fractions containing protein were determined from the 280 nm absorbance peaks on the chromatogram.
6	Prepare pre- and post-induction samples for electrophoresis. Spin the cells to collect pellet at 15000 RPM for 5 min. Resuspend the pellet in 500 µl nano pure water to reduce the salt concentration. Spin again at 15000 for 10 min. to collect the pellet. Add 20 µl 4x SDS loading dye and 60 µl nano pure water to the pellet. Resuspend the pellet and heat for 5 min at 95 °C. Prepare the eluted protein samples for electrophoresis. Add 5 µl 1x SDS running buffer, 2.5 µl 4x SDS loading dye and 2.5 µl eluted protein. Spin briefly to mix, heat at 95 °C for 5 min. 4-15 % SDS-PAGE BioRad. Load 2.5 µl protein ladder, 5 µl of pre- and post-induced samples, and 1 µl of eluted protein samples. Run at 180 V (constant) for 35 mins. Stain for 10 to 15 min. in Coomassie blue staining solution (can be reused for long time). Wash 3 times with d. water, followed by de-staining solution for 1 h to overnight (until the gel background is clear). Wash the gel with water for 10 min., image recorded using a gel image system (Bio-Rad)
7	Size exclusion column (Superdex 16/60 75 pg, 120 ml bed volume). Combine nickel column eluted fractions containing pure protein, concentrate using centicon-15 10K cut off, spin columns at 7500 RPM for 20−30 mins (4 °C). Load 1 to 2 ml concentrated protein onto equilibrated sizing column @ 1 ml/min flow rate (150 mM Tris HCl pH 7.4, 25 mM $MgSO_4$, and 300 KCl- PGM Buffer). Elute with 1 column volume PGM buffer at 0.5 ml/min. Identify the aggregated protein versus monomer on the chromatogram.
8	Prepare the eluted protein samples for electrophoresis. Add 5 µl 1x SDS running buffer, 2.5 µl 4x SDS loading dye and 2.5 µl eluted protein. Spin briefly to mix, heat at 95 ˚C for 5 min. Load 2.5 µl protein ladder, 5 µl of pre-and post-induced samples, and 1 µl of eluted protein samples onto a 4-15 % SDS-PAGE (Bio-Rad). Run at 180 V (constant) for 35 min. Stain for 10 to 15 min. in Coomassie blue staining solution (reusable). Wash 3x with d. water, followed by de-staining solution for 1 h to overnight (until the gel background is clear). Rinse the gel with water for 10 min., obtain a gel image (Bio-Rad). Use nano drop to measure the A_{280}, calculate the concentration using iPGM MW and ε_{280}
9	Add 20 % glycerol for long term storage, aliquot in to 10 to 20 µl volume and flash freeze in liquid nitrogen and store at -80 ˚C

Supplemental Protocol 2. Phosphoglycerate mutase ortholog functional assay panel

Phosphoglycerate mutase ortholog functional assay panel				
**Step**	**Parameter**	**Value**	**Target**	**Description**
1a	Reagent	4 μL	Bm iPGM	No Enzyme control and Enzyme solutions (5 nM Bm iPGM, 5 nM Ce iPGM, 5 nM Ov iPGM, 5 nM Di_ PGM, 50-500 nM Ec iPGM, and 5 nM Hs dPGM final concentrations; white/solid bottom high base plates (Greiner).
1b	Reagent	4 μL	Ce iPGM	
1c	Reagent	4 μL	Ov iPGM	
1d	Reagent	4 μL	Di iPGM	
1e	Reagent	4 μL	Ec iPGM	
1f	Reagent	4 μL	Hs dPGM	
2	Cyclic Peptides and Controls	23 nL		Cyclic Peptides [5 mM – 348 pM: 16-pt 1:3 Titration Series in duplicate]; and Ce2 and Ce-2d control peptides[1 mM – 69.6 pM: 16-pt 1:3 Titration Series in duplicate]; or vehicle (DMSO); Peptides transfer by Pintool
3	Incubation	>20 min		Peptide interaction with enzyme
4	Reagent	2 μL		3-phosphoglycerate (3PG)
5	Incubation	5-15 min		Ambient temperature; dark (all enzymes incubated for 5 min except for Ec iPGM incubated for 15 min at 37 ^{o}C)
6	4 μL	4 μL		Kinase-Glo Plus reagent
7	Incubation	10 min		Ambient temperature; dark
8	Measurement	ViewLux		Luminescence mode, 1 s expos; gain=med.; speed=slow; binning=2X

Step	Notes
1	Assay buffer: 30mM Tris-HCl, pH 8.0, 5 mM $MgSO_4$, 20 mM KCl, 0.12% BSA + 7.5 – 750 nM PGM enzyme.<br>• 5X Assay Buffer: 150 mM Tris-HCl pH 8, 25 mM $MgSO_4$, 100 mM KCl, 0.6% BSA
4	Substrate buffer for PGM enzymes: 30mM Tris-HCl, pH 8.0, 5 mM $MgSO_4$, 20 mM KCl, 9 mM ADP, and 0.15 units/ul each of enolase and pyruvate kinase (from rabbit muscle, prepared by Sigma) + 1.2 mM 3PG<br>5X Assay Buffer: 150 mM Tris-HCl pH 8, 25 mM $MgSO_4$, 100 mM KCl<br>• Final PGM assay buffer concentrations: 30mM Tris-HCl, pH 8.0, 5 mM $MgSO_4$, 20 mM KCl, 3 mM ADP, and 0.3 units each of enolase and pyruvate kinase + 5-500 nM PGM enzyme and 0.4 mM 3PG

Supplemental Protocol 3. *In vitro* biotinylation

***In vitro* biotinylation**			
**Sequence**	**Parameter**	**Value**	**Description**
1	Enzyme	100 µM	Purified protein with biotinylation sequence in PBS
2	Incubate	1 h at 37°C	Add $MgCl_2$, ATP, D-biotin and *E.coli* BirA to protein and incubate 1 h at 37°C
3	Incubation	1 h at 37°C	Add fresh D-biotin and *E.coli* BirA to the reaction and incubate 1 h at 37°C
4	Purify	Superdex 16/60 75 pg	Purify the reaction mixture
5	Efficiency	SDS-PAGE	Use streptavidin Alexa Fluor 488 conjugate to determine the biotinylation efficiency

**Step**	**Notes**
1	100 µM purified protein/enzyme with biotinylation sequence in 952 µl PBS in a 2 ml centrifuge tube
2	Prepare<br>1 mM $MgCl_2$ (203 mg $MgCl_2$ hexahydrate in 1 ml MilliQ Water)<br>100 mM ATP (55.1 mg of ATP in 1 ml MilliQ Water)<br>50 mM D-biotin (12.2 mg of D-biotin in 1 ml MilliQ Water)<br>50 µM *E.coli* BirA. (Purified protein with NTA affinity and size exclusion chromatography – see **Supplementary Protocol 1**)<br><br>Add 5 µl 1M $MgCl_2$, 20 µl 50 µM BirA, 20 µl 100 mM ATP and 3 µl 50 mM D-biotin to the 100 µM biotinylation sequence-fused protein in 952 µl of PBS. Incubate at 37°C for 1 h or (16°C overnight for thermally unstable proteins) with gentle mixing.
3	Add the same amount of fresh biotin and *E.coli* BirA and incubate for additional h at 37°C.
4	Load the biotinylated reaction mixture @ 1 ml/min flow rate, elute with PGM buffer @ 0.5 ml/min flow rate. Determine the fractions containing biotinylated protein using SDS-PAGE, then pool appropriate fractions. Use Amicon centrifugal concentrators to concentrate the protein and add glycerol to make 20 % final concentration. Determine the concentration and snap freeze aliquots in LN2.
5	Add 50 µM biotinylated protein (5 µl) and 10 µl of 16.67 µM streptavidin Alexa Fluor 488 conjugate together and incubate for 10 min. at ambient temperature. Add 5µl 4X SDS-loading dye and analyze 1 µl on a 4-15 % SDS-PAGE with protein ladder and negative controls. Stain one gel with Coomassie Brilliant Blue. Transfer one gel to PVDF membrane and image with Typhoon using Alexa 488 filter. Blot the PVDF membrane using anti Ce-iPGM Domain B antibody. Analyze the gel images to determine the efficiency of biotinylation.

Supplemental Protocol 4. Biacore S200 general experimental

Biacore S200 general experimental

Sequence	Parameter	Value	Description
1	Chip	Biotin CAP	S-series chip docked, primed 2X, regenerated surface and loaded with streptavidin
2	Enzyme	30-40 µg	Biotinylated enzymes loaded on to the surface
3	Clean	120 sec	Surface cleaned with one Ce-2d 200 nM 120 sec. injection
4	Kinetics	Multicycle	Kinetic data collected using several analyte concentrations 10-fold below and above KD
5	Regeneration	GE evaluation	Surface regenerated for next run
6	Data analysis	GE evaluation	Data processed using GE Biacore Evolution software

Step	Notes
1	• Eject the chip (usually maintenance chip) from the instrument: **Biacore Control Software>Tools>Eject chip**<br>• Dock the CAP chip on to the instrument: insert the chip then **Biacore Control Software>Tools>Dock Chip** (Buffer A should be in running buffer PBS-P 1X from Cytiva)<br>• Prime 2 times with running buffer (will take 10 to 15 min for each prime) **Biacore Control Software>Tools>Prime**<br>• Regenerate the surface with 3 parts regeneration A and 1 part regeneration B (8M guanidine HCl and 1 M NaOH) for 120 s with 10 µl/min flow rate manual mode for all four channels **Biacore Control Software>Run>Manual Run (save the file) Commands-Flow (10), Flow path (1234), Inject (select tube position and 120sec)**<br>• Load the surface with streptavidin (supplied in the kit from GE) for 300 s with 2 µl/min flow rate manual mode (~3500 RU for streptavidin loading, if not load for another 60 s) for all four channels **Commands-Flow (2), Inject (select tube position and 300 s)**<br>$mobilized\ ligand\ (RU) = \frac{ligand\ MW}{analyte\ MW}\ x\ analyte\ bindnig\ capcity\ (RU)$<br>$analyte\ bindnig\ capacity\ (RU) = \frac{analyte\ MW}{ligand\ MW}\ x\ immobilized\ ligand\ level\ (RU)$
2	• Channel 1, or channel 1 and 3 keep for reference, load ch.2 to 4 with same enzyme or different biotinylated enzymes (@40 µg/ml concentration) for 60 s manual mode with 10 µl/min flow rate and look for around 1000 increase in the RU, if needed load for another 15 s **Commands-Flow (10), Flow path (2/3/4), Inject (select tube position and 60 s)**<br>• Use below formula to estimate the RU enzyme loading, if working with other than iPGM and macrocyclic peptide
3	Use 200 nM Ce-2d for washing non-bound protein (potentially non-biotinylated or aggregated protein) off the chip with 120 s manual injection and run buffer over chip for several hours prior to kinetic experiments **Commands-Flow (10), Flow path (1234), Inject (select tube position and 120 s)**

4	Can use single cycle or multi cycle mode to set up the kinetic experiment. If we have enough reagents and time multi cycle experiment gives the best kinetic results (K_a, K_{off} and K_D)<br>• Aim for 10-fold below and 10-fold above K_D for lowest and highest analyte concentrations (not more than 100-fold higher)<br>• With K_D estimated from other technique use 6–7 concentrations of 3-fold dilutions, if K_D is unknown use 4–5 concentrations of 5-fold dilution to cover broad range in a short amount of time<br>• Build the method in **Commands-Template-Browse-Load method (modify from here or build new method)**<br>For only one sample **Start up-Sample (only two assay steps),** For additional samples add new assay step for each sample<br>Click on each assay step **Base Settings> Give name (start up or sample name)-Purpose(start up or sample)-Connect to cycle type (start up or sample); set the number of replicates** (3 for startup and 1 for sample); **Set Temperature (25)**<br>Click **Cycle Types>Start up> select High performance, Contact time (120 for startup), Dissociation time (120 for startup), flow rate (40/30), Flow path (1,2,3,4)**<br>Click **Sample> select High performance, Contact time (300 to 600 based on sample), Dissociation time (300 to 1200 based on sample), flow rate (40/30 based on injection time), Flow path (comes from assay in the later steps); check the Conc and MW in the method variables**<br>Check **Verification** for no errors<br>Click **Set up Run> select flow path (if ch.2 to 4 loaded with enzyme then 2-1,3-1,4-1, but only ch.2 and 4 loaded then 2-1,4-3), then click Next give sample name, conc (nM), MW, then click Next until you see Rack Positions, select the auto pooling from Menu (to cut down tubes number)**<br>• Prepare all the samples, load the tray with samples, and load tray into the instrument<br>Click Next to run the experiment (save the results file)
5	Once the experiment is completed regenerate the surface with 3 parts regeneration A and 1-part regeneration B (8M guanidine HCl and 1 M NaOH) for 120 s with 10 µl/min flow rate manual mode for all four channels **Biacore Control Software>Run>Manual Run (save the file) Commands-Flow (10), Flow path (1234), Inject (select tube position and 120 s)** Or set up a new experiment if the enzyme on the chip continues to perform well
6	• Once the experiment completes Evaluation software will open with the data<br>• Check the **sensorgrams (Inspection)**, check reference cells for any unusual patterns in the data indicating surface issues. With acceptable sensorgrams move to the Evaluation<br>• Click on the **Kinetics>**Select the curve type **reference subtracted,** Click the curves for processing, Click **Next**<br>• Use the left-hand side **Tools tab to crop off injection spikes (before and after injection),** If there are any additional spikes in the middle, or reach the base line earlier, use the right-hand side **Tools to remove the data**<br>• Use the **Settings>Fit Settings>Parameters> (change the parameters based on the situation)> Apply to selected or selected and cleared (based on the situation)> OK**<br>• Click **Fit (check selected or selected and Cleared, based on the situation)>OK**, gives you the fit with Kon, Koff and K_D<br>• Use the **Affinity (in the Evaluation)** to fit the steady state K_D; **check the curves (choose fit)>Next** Use the left-hand side **Tools tab to crop injection spikes (before and after injection),** If there are any more spikes in the middle or reach the base line earlier Use the right-hand side **Tools to remove the data**<br>• Use the **Settings>Fit Settings>Parameters> (change the parameters based on the situation)> Apply to selected or selected and cleared (based on the situation)> OK**<br>Click **Fit (check selected or selected and Cleared, based on the situation)>OK**, provides the K_D (for runs reaching saturation at each analyte concentration, otherwise use the K_D from the Kinetics Plot)
